# Comprehensive analysis of full genome sequence and *Bd*-milRNA/target mRNAs to discover the mechanism of hypovirulence in *Botryosphaeria dothidea* strains on pear infection with BdCV1 and BdPV1

**DOI:** 10.1186/s43008-019-0008-4

**Published:** 2019-06-07

**Authors:** Wangcheng Hu, Hui Luo, Yuekun Yang, Qiong Wang, Ni Hong, Guoping Wang, Aiming Wang, Liping Wang

**Affiliations:** 1 State Key Laboratory of Agricultural Microbiology, Wuhan, Hubei 430070 People’s Republic of China; 2 College of Plant Science and Technology, Huazhong Agricultural University, Wuhan, Hubei 430070 People’s Republic of China; 3 Key Lab of Plant Pathology of Hubei Province, Wuhan, Hubei 430070 People’s Republic of China; 4 London Research and Development Centre, Agriculture and Agri-Food Canada, London, ON N5V 4T3 Canada

**Keywords:** Pear ring rot, *Botryosphaeria dothidea*, Mycovirus, *Botryosphaeria dothidea* chrysovirus 1 (BdCV1), *Botryosphaeria dothidea* partitivirus 1 (BdPV1), microRNA, RNA-silencing

## Abstract

Pear ring rot disease, mainly caused by *Botryosphaeria dothidea*, is widespread in most pear and apple-growing regions. Mycoviruses are used for biocontrol, especially in fruit tree disease. BdCV1 (*Botryosphaeria dothidea* chrysovirus 1) and BdPV1 (*Botryosphaeria dothidea* partitivirus 1) influence the biological characteristics of *B. dothidea* strains. BdCV1 is a potential candidate for the control of fungal disease. Therefore, it is vital to explore interactions between *B. dothidea* and mycovirus to clarify the pathogenic mechanisms of *B. dothidea* and hypovirulence of *B. dothidea* in pear. A high-quality full-length genome sequence of the *B. dothidea* LW-Hubei isolate was obtained using Single Molecule Real-Time sequencing. It has high repeat sequence with 9.3% and DNA methylation existence in the genome. The 46.34 Mb genomes contained 14,091 predicted genes, which of 13,135 were annotated. *B. dothidea* was predicted to express 3833 secreted proteins. In bioinformatics analysis, 351 CAZy members, 552 transporters, 128 kinases, and 1096 proteins associated with plant-host interaction (PHI) were identified. RNA-silencing components including two endoribonuclease Dicer, four argonaute (Ago) and three RNA-dependent RNA polymerase (RdRp) molecules were identified and expressed in response to mycovirus infection*.* Horizontal transfer of the LW-C and LW-P strains indicated that BdCV1 induced host gene silencing in LW-C to suppress BdPV1 transmission. To investigate the role of RNA-silencing in *B. dothidea* defense, we constructed four small RNA libraries and sequenced *B. dothidea* micro-like RNAs (*Bd*-milRNAs) produced in response to BdCV1 and BdPV1 infection. Among these, 167 conserved and 68 candidate novel *Bd*-milRNAs were identified, of which 161 conserved and 20 novel *Bd*-milRNA were differentially expressed. WEGO analysis revealed involvement of the differentially expressed *Bd*-milRNA-targeted genes in metabolic process, catalytic activity, cell process and response to stress or stimulus. BdCV1 had a greater effect on the phenotype, virulence, conidiomata, vertical and horizontal transmission ability, and mycelia cellular structure biological characteristics of *B. dothidea* strains than BdPV1 and virus-free strains. The results obtained in this study indicate that mycovirus regulates biological processes in *B. dothidea* through the combined interaction of antiviral defense mediated by RNA-silencing and milRNA-mediated regulation of target gene mRNA expression.

**Electronic supplementary material:**

The online version of this article (10.1186/s43008-019-0008-4) contains supplementary material, which is available to authorized users.

## INTRODUCTION

Pear (*Pyrus*) is an important fruit crop, with rich pear germplasm resources including thousands of cultivars belonging to five domesticated species of *Pyrus pyrifoila*, *Pyrus communis*, *Pyrus bretschneideri*, *Pyrus ussuriensis* and Kuerle pear, which are grown all over China (Wu et al. 2018). However, pear stem wart and stem canker diseases induced by *Botryosphaeria* species are widespread across all pear producing areas, inducing rotten fruit, branch warts, branch dieback, stem canker, and pear tree growth recession. *B. paeva*, *B. rhodina*, *B. obstuse* and *B. dothidea* are the dominant species that seriously affect pear crop yield and quality (Slippers et al. 2004; Slippers and Wingfield 2007; Garibaldi et al. 2012; Yan et al. 2013; Zhai et al. 2014; Wang et al. 2017b; Yang et al. 2017b). *Botryosphaeria* spp., belonging to *Botryosphaeriaceae* in the class of *Dothideomycetes*, destroy many types of fruit trees such as apple, pear, grapevine, peach, plum, apricot, begonia, chestnut and jujube (Slippers and Wingfield 2007; Garibaldi et al. 2012; Tang et al. 2012; Yan et al. 2013; Zhai et al. 2014). *B. dothidea* is the main destructive pathogen and dominant strain responsible for pear ring rot disease in pear-growing areas (Zhai et al. 2014). Despite the importance of *B. dothidea*, little is known about the genetics and pathogenic mechanism of this pathogen (Wang et al. 2018a, 2018b). To date, the main strategy used to reveal the mechanism of phytopathogenic infection has involved sequencing of the complete genome and annotation of gene function in fungal diseases such as rice sheath blight, rice blast, and wheat stripe rust pathogen fungus, *Cenococcum geophilum* and *Cercospora sojina* (Dean et al. 2005; Zheng et al. 2013a, 2013b; Dong et al. 2015; Peter et al. 2016; Luo et al. 2018; Plissonneau et al. 2018). Similarly, sequencing of the *Ustilago esculenta* genome and gene annotation revealed the molecular mechanism of the artificial selection and smut fungi-host interaction involved in RNA-silencing in host defense (Ye et al. 2017). Therefore, full genome sequencing and analysis of the *B. dothidea* LW-Hubei strains infecting pear hosts will provide important molecular information that can be used to explore the pathogenic mechanisms.

Although the application of numerous chemical fungicides and fruit bagging techniques have contributed to the prevention pear ring rot disease, the associated environmental pollution has serious detrimental effects on food safety and health (Guo et al. 2009; Zhao and Cao 2012; Zhou et al. 2012). Thus, new, safe and effective measures for the biocontrol of pear ring rot disease induced by *B. dothidea* are urgently needed. Mycovirus-mediated hypovirulence in plant pathogenic fungi has become an important strategy for the biocontrol of fungal diseases (Anagnostakis 1982; Nuss 2005; Chiba et al. 2009; Ghabrial and Suzuki 2009; Pearson et al. 2009; Yu et al. 2013; Xie and Jiang 2014). Recently, hypovirulence of *B. dothidea* LW-C strain infected with *B. dothidea* chrysovirus 1 (BdCV1) has been reported as a potential candidate for the biocontrol of fungal diseases (Wang et al. 2014; Wang et al. 2017b) Studies of the molecular basis of hypovirulence of BdCV1-infected *B. dothidea* have provided further clarification of the pathogenesis of *B. dothidea* and provided a theoretical basis for the prevention and control of disease caused by this fungus.

RNA-silencing is a conserved mechanism in eukaryotic organisms, in which small RNAs (sRNAs), particularly microRNAs (miRNAs), regulate transcriptional or post-transcriptional gene silencing (Kim et al. 2009; Huntzinger and Izaurralde 2011; Bologna and Voinnet 2014). sRNAs are involved in regulation of morphological and developmental processes in response to abiotic and biotic stresses (Dang et al. 2011; Wang et al. 2016a, 2016b). However, studies on sRNA-mediated gene silencing in fungi and mycoviruses are rare (Li et al. 2010; Nuss 2011; Chiba et al. 2013). RNA-silencing as a mechanism of sRNA-based antiviral defense requires the involvement of *Dicer*, *Ago*, *RdRp* genes, which regulate sRNA biosynthesis and mycovirus expression in fungi (Kadotani et al. 2004; Segers et al. 2007; Janbon et al. 2010; Campo et al. 2016). In fungi, sRNAs suppress plant immunity by hijacking host RNAi pathways, as reported for *Botrytis cinerea*, *Rhizoctonia solani*, Verticillium wilt and *Puccinia striiformis* (Wang et al. 2016a, 2016b; Weiberg et al. 2013, 2014; Wang et al. 2017a, 2017c; Cai et al. 2018). sRNAs can be transported between plants and fungi to silence virulence gene, indicating that resistance based on cross-kingdom sRNA transport has extensive application potential for the prevention and control of various plant diseases (Weiberg et al. 2013, 2014; Cai et al. 2018). High-throughput sequencing technologies and bioinformatics analysis have been used to identify microRNAs (miRNAs) in some plant pathogenic fungi, such as *Rhizoctonia solani*, *Neurospora crassa*, *Puccinia striiformis, Cryptococcus neoformans*, *Trichoderma reesei*, *Sclerotinia sclerotiorum*, and *Fusarium graminearum*, *F. oxysporum*, and *Penicillium marneffei* (Lee et al. 2010; Jiang et al. 2012; Zhou et al. 2012; Kang et al. 2013; Lau et al. 2013; Chen et al. 2014, 2015; Dahlmann and Kück 2015; Mueth et al. 2015; Lin et al. 2016). Mature miRNAs negatively regulate gene expression by complementary binding to the open reading frame (ORF) or untranslated region (UTR) of specific target genes, although the mechanism of miRNA-mediated regulation of target mRNAs remains poorly understood in most fungal species (Lee et al. 2010; Li et al. 2010; Dang et al. 2011; Huntzinger and Izaurralde 2011). RNAi components and sRNAs have been identified in *P. striiformis*. *Pst*-milR1 was identified as an important pathogenicity factor that impairs wheat resistance by regulation of the pathogenesis-related 2 gene (*PR2*) in wheat, and *Ps*DCL was found to regulate *Pst*-milR1 production (Mueth et al. 2015; Wang et al. 2017a). Therefore, miRNA-mediated regulation of biological processes may be involved in different biological pathways in host fungi (Li et al. 2010; Huntzinger and Izaurralde 2011).

As the causative agent of pear ring rot, *B. dothidea* is associated with serious agricultural losses. Some studies of the genome from *B. dothidea* infecting with apple and grapevine hosts, and transcriptome of *B. dothidea* infecting pear hosts in response to mycovirus, have been reported; however, the sRNAs in *B. dothidea* remain to be identified (Garibaldi et al. 2012; Zhai et al. 2014; Liu et al. 2016; Wang et al. 2018a, 2018b). The existence of microRNA-like RNAs (milRNAs) in *B. dothidea* and the potential mechanism by which these molecules regulate their target genes to counter pathogen infections remains to be established. In our previous study, we used de novo transcriptome sequencing to show that the *DCL*, *Ago* and *RdRp* genes were expressed in *B. dothidea* strains in response to mycovirus infection (Wang et al. 2018b). RNA-silencing has been predicted to exist in *B. dothidea* strains, although this remains to be confirmed. Furthermore, the roles of RNA-silencing components in sRNA transcription and milRNA generation in *B. dothidea* infected with mycovirus remain to be clarified.

Mycoviruses are used for biocontrol, especially in fruit tree disease. BdCV1 (*Botryosphaeria dothidea* chrysovirus 1) and BdPV1 (*Botryosphaeria dothidea* partitivirus 1) belonging to dsRNA mycovirus, influence the biological characteristics of *B. dothidea* strains. BdCV1 is a potential candidate for the control of fungal disease. Therefore, it is vital to explore interactions between *B. dothidea* and mycovirus to clarify the pathogenic mechanisms of *B. dothidea* and hypovirulence of *B. dothidea* in pear. In this study, we sequenced the entire genome of *B. dothidea* and constructed four sRNA libraries of *B. dothidea* infected with mycovirus to examine the evidence for RNAi induction and milRNA expression in response to mycovirus in hypovirulent and non-hypovirulent *B. dothidea* strains. This information is important in providing insights into milRNAs as potential pathogenic factors and elucidating the molecular basis of the interaction of *B. dothidea* with mycovirus.

## MATERIALS AND METHODS

### B. dothidea strains, conidia and hyphal morphological analysis by light microscopy

The *Botryosphaeria dothidea* LW-1 (designated as LW-CP) strain, with a mixed infection of *Botryosphaeria dothidea* chrysovirus 1 (BdCV1) and *Botryosphaeria dothidea* partitivirus 1 (BdPV1), was isolated from sand pear (*Pyrus pyrofolia*) trunk in Hubei Province, China, as previously described (Wang et al. 2014). The virulent *Botryosphaeria dothidea* LW-P strain infected with BdPV1, the attenuated *Botryosphaeria dothidea* LW-C strain infected with BdCV1, and *Botryosphaeria dothidea* Mock strain (designated as LW-Hubei) as a virus-free control, were used in this study (Wang et al. 2017b). In addition, we used the HL-1 and HBWH-1 *B. dothidea* strains isolated from sand pear trunks collected from Hubei Province (Wang et al. 2014; Wang et al. 2017b).

Colonies of the four *B. dothidea* strains (LW-CP, LW-C, LW-P and Mock) were cultured for 3 days on potato dextrose agar (PDA) and Murashige Skoog (MS) at 25 °C in darkness, respectively. To induce sporulation, the mycelia were harvested and exposed to blank light for 11 d. Sporulation and conidial angle were observed under a stereo microscope (Olympus, SZX16; Japan). Conidia were stained with DAPI (Solarbio, Beijing of China) and conidial size was measured from images obtained under DAPI filter using a fluorescence microscope (Leica DM2500, Germany) (Tebeest et al. 1989). In addition, the mycelia were cultured for 3 d on agarose gel to observe the morphology and assess the effect of mycovirus on the biological characteristics of *B. dothidea*.

### Evaluation of the cellular morphology of B. dothidea strain mycelia by transmission electron microscopy (TEM)

The four samples of mycelium from *B. dothidea* strains were cultured for 48 h before collection. The samples were then fixed with 2.5% glutaraldehyde for 2 h at room temperature (RT), rinsed three times with 0.1 M phosphate-buffered saline (pH 7.4) and subsequently fixed in 1% osmic acid for 2 h at 4 °C. The four samples were dehydrated in a graded ethanol series (50, 70, 80, 85, 90, 95 and 100%), permeated successively with 2:1 and 1:1 acetone and epoxy as a penetrating agent, and embedded in ethoxyline resin. Ultra-thin sections (thickness, 60–100 nm) were prepared with a diamond microtome (Leica, EM UC7, Germany) and collected onto Formvar-coated copper grids. The sections were then stained with lead and uranium for observation of the cellular morphology and damage to the phytopathogenic fungi caused by mycovirus infection using a TEM (America FBI company, Cat. Tecnai G^2^ 20 TWIN) under a 200-kV accelerating voltage.

### Evaluation of B. dothidea strain pathogenicity

The pathogenicity of *B. dothidea* strains was evaluated on apple and pear fruit, and pear branches as previously described with a little modification (Wang et al. 2014). The mycelial plugs from strains were cultured on PDA for 3 d to obtain fresh samples for inoculation. The inoculation was performed using the wounding method and the expansion of the lesion area was observed and measured.

### Vertical transmission of BdCV1 and/or BdPV1 to B. dothidea LW-Hubei strain

Single conidia were obtained from the induced pycnidia of *B. dothidea* strains LW-C, LW-P and LW-CP generated as described in the previous section. The derivatives were screened for BdCV1 and/or BdPV1 dsRNAs to assess vertical transmission ability and stability of BdCV1 and BdPV1 in *B. dothidea* strains (Yang et al. 2017a, 2017b).

### Horizontal transmission between BdCV1 and BdPV1 in B. dothidea strains

Transmission of hypovirulence traits of strain LW-CP, LW-C and LW-P was assessed according to a previous method with some modifications (Wang et al. 2017b). *B. dothidea* strains were co-cultured at 25 °C for 7 days to allow contact between the two colonies. The hypovirulent strains LW-CP and LW-C, and the virulent strain LW-P served as the donors, and the virus-free strain Mock served as the recipient. In addition the LW-C and LW-P strains were also used as both recipient and donor, respectively, to evaluate BdCV1 and BdPV1 transmission between the two strains. After incubation of the contact cultures, mycelia agar plugs from the colony margin of the Mock, LW-C and LW-P strains were transferred to a fresh PDA plate to obtain three derived isolates from each co-cultured recipient strain. The derivatives were screened for dsRNAs as described previously (Yang et al. 2017a). The parental strains LW-CP, LW-C and LW-P were included as controls.

### B. dothidea strain information and genomic DNA isolation

The mycelia were cultured for 5 d at 25 °C on PDA containing 50 μg/ml ampicillin and streptomycin. The mycelia were then collected and ground in liquid nitrogen. Genomic DNA from the Mock strain (designated as the LW-Hubei isolate) were isolated using the standard cetyltrimethylammonium bromide (CTAB) method with some modifications (Murray and Thompson 1980). The gDNA titer and quality were assessed by Qubit Fluorometer, agrose gel electrophoresis and NanoDrop spectrophotometer prior to library construction.

### Genome sequencing, assembly and annotation

The genomic DNA of the *B. dothidea* LW-Hubei isolate was sequenced using the Single Molecule Real-Time (SMRT) method at the Beijing Genomics Institute (BGI) in Shenzhen, China (Chin et al. 2013). A 270-bp paired-end library and a 10-kb mate-pair DNA library were constructed, and subsequently subjected to paired-end 150-bp sequencing using the Illumina HiSeq4000 platform. The treated sequencing data (filtered reads: 6.84 G, sequencing depth: 147×) were used to estimate the genome size, repeat content, and genome heterozygosis by K-mer analysis and then confirmed by alignment between assembled scaffolds and original reads. The 20-kb library was then constructed according to standard PacBio methods, including genomic DNA fragmentation, end-repair, adaptor ligation, and template purification. The 20-kb library was quantified using a 2100 Bioanalyzer (Agilent, USA) and sequenced by SMRT. The raw data generated by the PacBio platform were treated according to standard protocols. The reads were assembled using variety of software in combination with bioinformatics analysis of read assemblies, contig linkage to scaffolds and gap-filling (Kim et al. 2014; Badouin et al. 2015; Faino et al. 2015; Sit et al. 2015; Tsuji et al. 2015). The sequencing data (filtered reads: 3.72G, sequencing depth: 80×) were assembled by CANU (Version-1.2) with default parameters and Falcon (Version-0.3.0).

Protein-encoding genes were annotated by a combination of independent ab initio predictors based on homology (Johnson et al. 2008; Stanke et al. 2008; Ter-Hovhannisyan et al. 2008). The transcriptome data obtained from the four libraries of LW-Hubei strains were mapped to the genome using Trinity to assess the quality of *B. dothidea* genome assembly and annotation (Wang et al. 2018b).

### Functional annotation of predicted genes

Functional annotation of all predicted genes was performed using multiple databases including Swiss-Prot, NR (NCBI non-redundant protein sequences), KEGG (Kyoto Encyclopedia of Genes and Genome), GO (Gene Ontology), COG (Cluster of Orthologous Groups) and KOG by BlastP with a cut-off *E*-value of 10^− 5^ (Ashburner et al. 2000; Jones et al. 2014; Galperin et al. 2015; Makarova et al. 2015; Kanehisa et al. 2016). In addition, potential virulence-related proteins were identified by searching against the pathogen-host interaction database (PHI) (Torto-Alalibo et al. 2009). The following databases were used to analyze genes from the *B. dothidea* LW-Hubei isolate: fungal cytochrome P450 database, carbohydrate-active enzymes (CAZy) database (http://www.cazy.org/), virulence factor database (VFDB), type III secretion system effector protein (T3SS), and transporters (Fischer et al. 2007; Vargas et al. 2012; Levasseur et al. 2013; Chen et al. 2016; Elbourne et al. 2017). Putative kinase proteins were predicted and annotated using the databases (http://ekpd.biocuckoo.org/download/HMM/kinase.hmm).

### Repeat annotation, DNA methylation analysis and synteny annotation

A de novo repeat database of the *B. dothidea* LW-Hubei isolate was generated using Repbase, ProMask, the De novo database and the Tandem Repeats Finder (TRF) (Benson 1999). Transposable elements (TEs) were identified by aligning the assembly with the known transposon sequence database and the De novo database. The software package used for predication repeat annotation contains Repeat Masker (Version: 4–0-6), Repeat Protein Masker and the De novo database (Smit et al. 2014). Tandem repeats (TR) were predicted by TRF (Version: 4.04). Whole-genome DNA modification detection and motif analysis were performed using the PacBio SMRT software (version 2.3.0, http://www.pacb.com) (Fang et al. 2012; Davis et al. 2013; Manso et al. 2014; Adhikari and Curtis 2016; Cohen et al. 2016). The whole genome syntenic analysis of *B. dothidea* LW-Hubei isolate compared with *Macrophomina phaseolina*, *Neofusicoccum parvum*, *Diplodia corticola* and *Diplodia seriata* was performed with MUMmer (version 3.0) (Fu et al. 2012). Linear synteny graphs were constructed at the nucleic acid and amino acid levels (*E*-value ≤1e^− 5^, sequence identity ≥85% and the best hit for each protein were selected). In addition, a schematic representation of the synteny between the selected scaffold1 of the *B. dothidea* LW-Hubei isolate with those of the four strains was also generated with CIRCOS (http://mkweb.bcgsc.ca/circos/).

### Comparative genomic analysis of gene families

The protein sequences of the *B. dothidea* LW-Hubei isolate genome was compared with those of four pathogenic species of *Botryosphaeriaceae* family (*M. phaseolina*, *N. parvum*, *D. corticola* and *D. seriata*). OrthoMCL (version 2.0) was used to analyze the orthologs, co-orthologs and in-paralog pairs. Gene families were constructed and analyzed based on the genes from *B. dothidea* LW-Hubei isolate and the other fungi as reference strains. In addition, single-copy gene family analysis was performed according to the BGI protocols, including alignment of the protein sequence in BLAST, gene family TreeFam clustering with Hcluster_sg software, and conversion of the alignment proteins into those of the multiple amino acid sequences in the CDS region using MUSCLE-3.8 (Edgar 2004a, 2004b).

### Phylogenetic tree analysis

The phylogenetic relationships between *B. dothidea* LW-Hubei and the four *Botryosphaeriaceae* isolates (*M. phaseolina, N. parvum, D. corticola, D. seriata*) as well as four additional *Dothideomycete* isolates (*Cenococcum geophilum*, *Dothistroma septosporum, Exserohilum turcica, Lepidopterella palustris*) were analyzed at amino acid level based on the consistent phylogenetic core-pan gene and single-copy orthologous families. The sequence alignments were performed using MUSCLE-3.8(Edgar 2004b; Nandi et al. 2010).

The phylogenetic tree was generated by MEGA 7 using a TreeBeST method, with the bootstrap value set at 1000 replicates (Kumar et al. 2016).

### RNA preparation and isolation

The mycelia of the mycovirus-infected three strains and the virus-free strain of *B. dothidea* were collected after culture on PDA for 5 days and then ground under liquid nitrogen. The total RNAs (tRNAs) were extracted using TRIzol reagent (Invitrogen) according to the manufacturer’s instructions with some modifications. The concentration and integrity of the tRNAs was further assessed using a NanoDrop spectrophotometer and agarose gel electrophoresis. High quality tRNAs were used for high-throughput sequencing of sRNA, verification of milRNAs and mRNA expression analysis.

### Small RNA library construction and deep sequencing

Small RNA libraries were constructed using 20 μg of tRNA and the kits of PAGE gel separation of RNA segments of different size to cut out between 18 and 30 nt strip (Illumina, USA) according to the manufacturer’s protocols for sRNA fraction isolation, adapter ligation, reverse transcription (RT) (Invitrogen) in combination with adapter-specific RT-primers, PCR amplification and purification by PAGE gel electrophoresis. The quality and yield of purified high-quality cDNA library were detected using Agilent 2100 Bioanalyzer and ABI StepOnePlus Real-Time PCR System, and sequenced on an Illumina Genome AnalyserIIx (BGI, Shenzhen, China).

### Identification of milRNAs and expression by bioinformatics analysis

High-quality reads obtained from the four *B. dothidea* strains by elimination of the low-quality reads, adaptors and other contaminants to obtain 18–30 nt clean reads. sRNAs with perfect genomic matches were used for further analysis. All trimmed sequences between 18 and 30 bp in length were used to align clean reads (tags) to the reference genome in the study and to other sRNA in databases to identify known milRNA by Bowtie (Langmead et al. 2009). Alignments with no gap and two mismatches between the query sequences and known milRNAs were used in subsequent analysis. The reads that did not match any known miRNAs were further processed to discover putative novel milRNAs with their predicted hairpin precursors using miRDeep2 software (Friedlander et al. 2008). To further confirm precursor stem-loop structure of these predicted novel milRNAs, the putative precursor sequence was subjected to folding analysis using RNAfold software. In addition, the sRNA expression levels were estimated in terms of Transcripts Per Kilobase Million (TPM). The milRNA gene expression levels were used directly to determine differential gene expression compared with that of the *B. dothidea* strains in response to mycovirus infection. To evaluate the significance of differential gene expression, FDR ≤0.001 and the absolute value of |log2FC| ≥ 1 were set as the default threshold.

### Bd-milRNA and mRNA expression analysis in B. dothidea strains

The *Bd*-milRNAs were detected by stem-loop RT-PCR (Chen et al. 2005). Briefly, a stem-loop RT primer was used to reverse-transcribe mature milRNAs to obtain cDNAs. First, 500 ng tRNAs and 0.25 μM of each individual stem-loop RT primer were added to be 10 μl RT reaction mixture and incubated at 95 °C for 5 min before placing on ice immediately. Following the addition of 2 U RNase inhibitor (Yeasen, Shanghai city of China), 0.5 mM dNTPs (Takara, Dalian of China), cDNA was generated using the PrimeScript™ RT Kit with genomic DNA Eraser (Takara, Dalian of China) in a 20 μl RT reaction mixture, which was incubated at 42 °C for 60–90 min.

MilRNAs were amplified by polyadenylation-mediated PCR. sRNA was isolated using the miRcute miRNA Isolation Kit (Tiangen) according to the manufacturer’s protocol. The template was polyadenylated, and reverse transcribed to cDNA using the Poly (A) miRNA cDNA Synthesis Kit (Invitrogen) according to the manufacturer’s instructions. MilRNAs were amplified from the cDNA pool by using the mature miRNA-specific forward and reverse primers provided in the kit. The PCR products were then separated by agarose gel electrophoresis.

In addition, *B. dothidea* mRNA expression was evaluated by RT-qPCR analysis using 2.5 μl of diluted cDNA as a template and the primers described in Additional file 17: Table S1 and Additional file 18: Table S2. The reaction mixture contained 10 μl of the SYBR Premix *Ex Taq II* PCR mixture (Tli RNaseH Plus) (Takara, Dalian of China), 1 μl of each 5 mM forward and reverse primer, and deionized water to a final 20 μl volume. All reactions were run in triplicate on a CFX96 Real-time System (BIO-RAD, USA). The products were verified by melting curve analysis. The *actin* gene (GME8592_g) was included as an internal reference for each sample. The relative mRNA expression level changes were calculated using a comparative CT method (ΔΔCT) according to the formula 2^-ΔΔCT^ (Livak and Schmittgen 2001).

### Prediction of milRNA targets and function annotation

The potential target mRNAs of known and novel milRNAs were predicted using two types of software. The predicted milRNA target cleavage sites were identified by alignment of the mature milRNA sequences with reported *B. dothidea* RNA-seq data**,** which was reanalyzed based on the complete genome sequence of *B. dothidea* LW-Hubei using psRNATarget with default parameter values set as a cut-off threshold of 2.5 with the expectation of higher prediction coverage. In addition, TargetFinder software was used to predict the putative target mRNAs (Audic and Claverie 1997; Fahlgren and Carrington 2010; Dai and Zhao 2011). The KEGG pathway was also used to perform pathway enrichment analysis of the predicted biological function of the differentially expressed target genes (Kanehisa et al. 2008). In addition, the functional enrichment analysis was performed using WEGO online (http://wego.genomics.org.cn/) (Ye et al. 2006).

### Statistical analysis

All experiments were performed in triplicates or more. Data were presented as mean ± SD and analyzed using SPSS (Version 21.0) software. Data were further evaluated using Duncan’s new multiple range test. *P* < 0.05 was considered to indicate statistical significance.

## RESULTS

### The interaction between dsRNA mycoviruses and B. dothidea strains

BdCV1 and BdPV1 influenced the biological characteristics of the *B. dothidea* LW-Hubei strain. In the current study, BdCV1 reduced the virulence of LW-C and LW-1 (designated as LW-CP) inoculation on ‘*Malus domestica* cv. Fuji’ apple fruit, *Pyrus pyrifolia* cv. ‘Hohsui’ pear fruit and one-year branch growth, whereas BdPV1 enhanced the virulence of LW-P. Virus-free Mock (designated as LW-Hubei strain), HL-1 and HBWH-1 exhibited virulence as controls (Additional file 1: Figure S1). These findings are in accordance with our previous results showing hypovirulence of LW-C and LW-1 in ‘Huangguang’, ‘Kuerle’, and *Pyrus bretschneideri* Rehd. cv. ‘Mili’ pear fruit and *P. pyrifolia* Nakai cv. ‘Hongxiangsu’ pear branches (Wang et al. 2014; Wang et al. 2017b). These results further demonstrated that BdCV1 is associated with hypovirulence of *B. dothidea* infecting different pear cultivars and apple hosts; thus, BdCV1 is implicated as a promising candidate for biological control of pear ring rot disease.

**Fig. 1:**
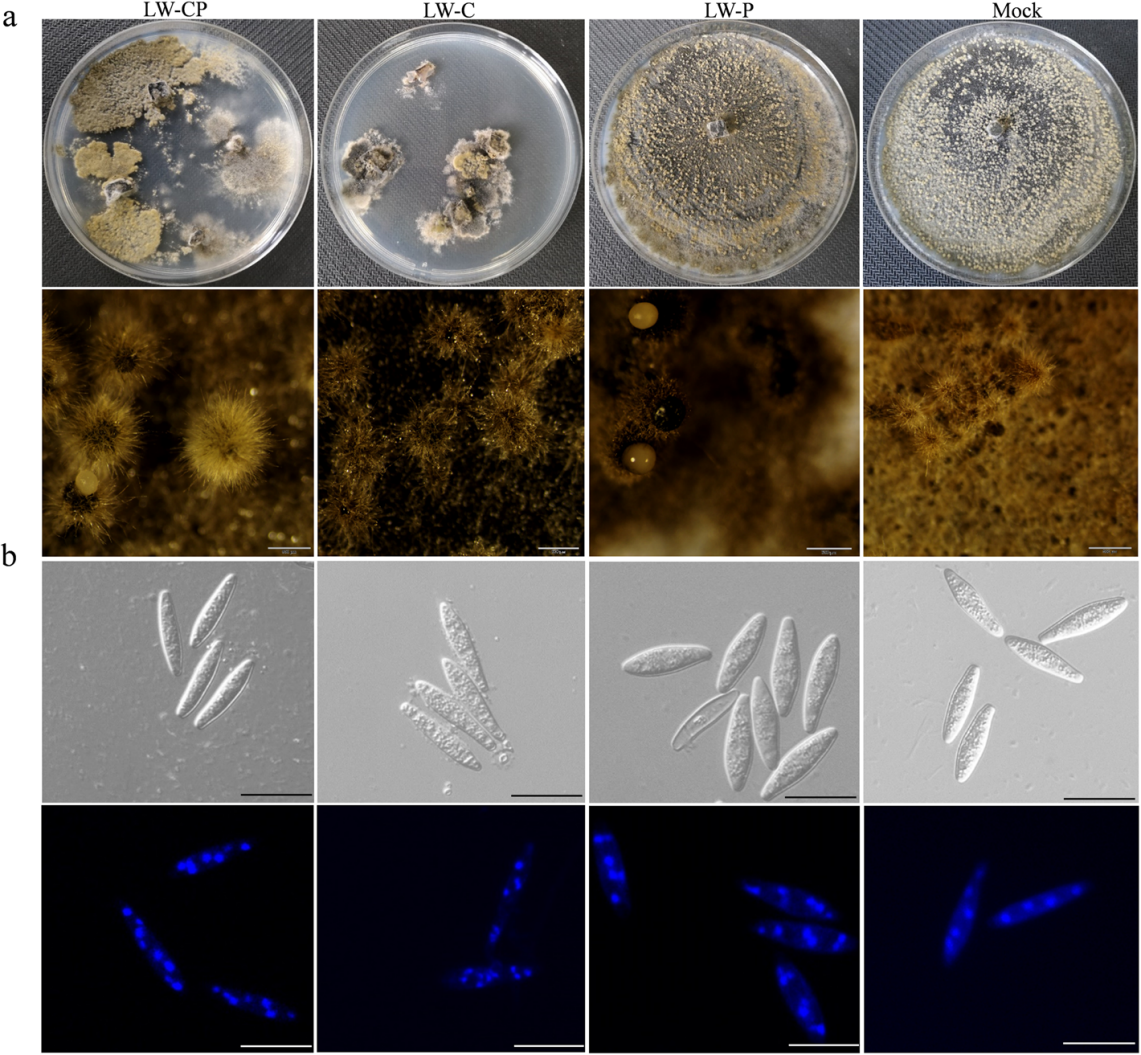
The conidiomata and conidia phenotype of *Botryosphaeria dothidea* strains with mycovirus. **a** Conidiomata and conidial angle of *Botryosphaeria dothidea* strains (LW-CP, LW-C, LW-P and Mock) on potato dextrose agar (PDA) was observed with the naked eye (upper row) and using stereo microscope (bottom row). Scale bar = 0.5 mm; **b** The phenotype of conidia of *B. dothidea* strains cultured on PDA culture observed following DAPI staining. Scale bar = 20 μm

**Fig. 2:**
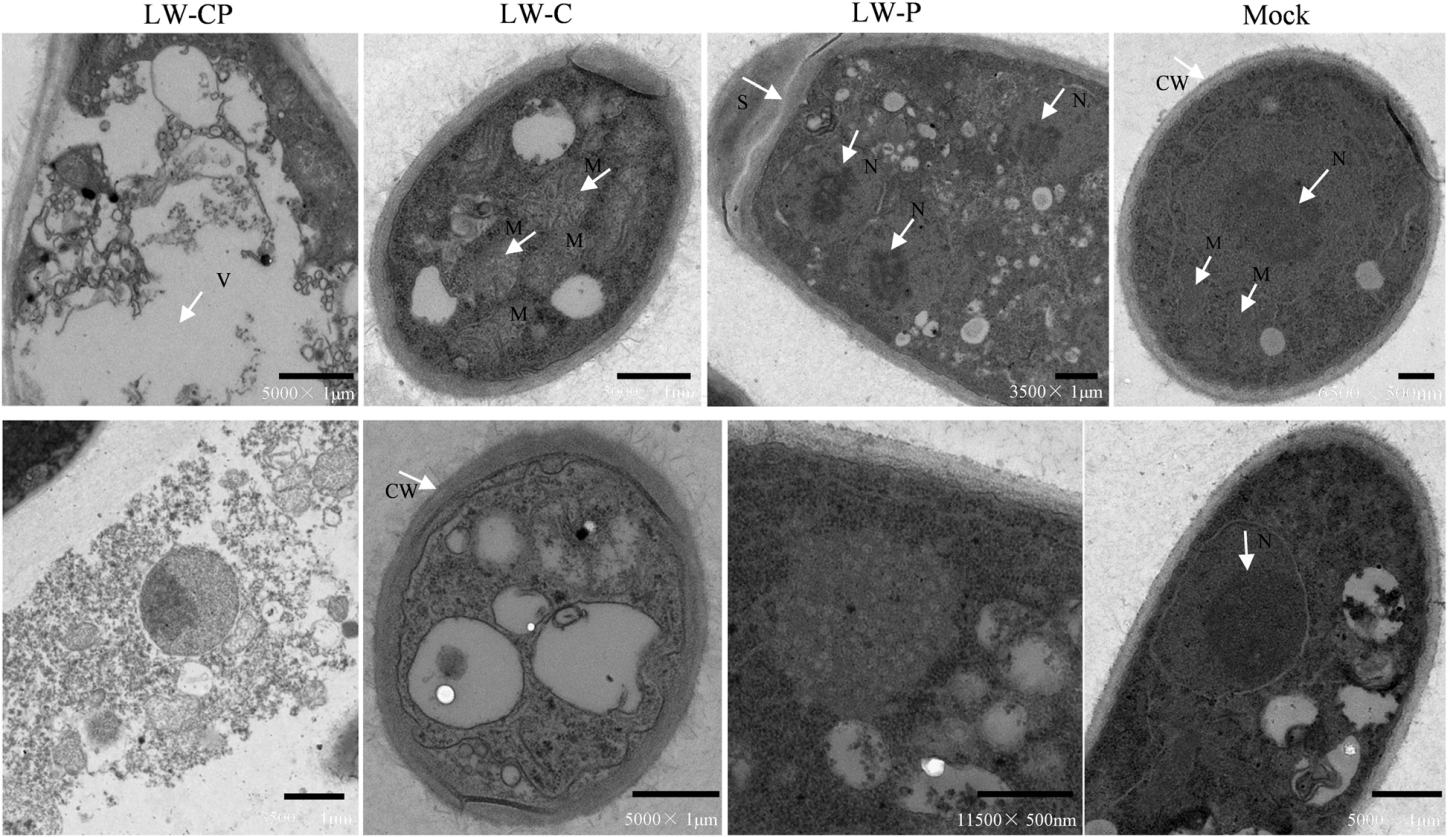
Microscopic observation cell ultrastructure of fungal hyphae from *Botryosphaeria dothidea* strains (LW-CP, LW-C, LW-P and Mock) cultured on PDA for 48 h. V, vacuole; N, nucleus; CW, cell wall; M, mitochondria. The magnification and Scale bar was shown on the images

In addition, we analyzed the effects of BdCV1 and BdPV1 on conidial bodies, microscopic morphology and cell ultrastructure of the mycelium from *B. dothidea* LW-Hubei strains. The LW-P strain produced conidiomata and conidium angle more effectively than the LW-C and LW-CP strains when cultured on PDA and MS medium, respectively (Fig. 1a, Additional file 2: Figure S2). The LW-P and Mock strains produced solitary, globular pycnidia covered with mycelia, whereas the condial bodies of the LW-CP and LW-C strains formed a yellow mass. The conidia produced by LW-P were longer than those produced by LW-C, LW-CP and Mock (Fig. 1b, Additional file 19: Table S3). Observation of the conidial growth and morphology revealed that the four strains produced hyaline conidia, with septa rarely formed before germination, and predominantly containing eight nuclei (Fig. 1b, Additional file 3: Figure S3). Observation of the mycelia produced after 48 h in culture by transmission electron microscopy (TEM) showed that the cell structure of the LW-C and LW-CP strains was markedly different from that of the Mock and LW-P strains. The cells of the LW-C and LW-CP strains were small and deformed, with irregular thickening and some degradation of the cell wall, exosome formation and plasmolysis of the protoplasm, swollen mitochondria, and heavily vacuolization. In contrast, the cell wall and membrane in the LW-P and Mock strains were uniform and smooth, and the nucleus profile was clearly visible (Fig. 2). Microscopic observation of *B. dothidea* strains cultured on PDA for 3 d showed that the hyphae from LW-C and LW-CP were deformed and with more branches compared with those of LW-P and Mock (Additional file 4: Figure S4).

**Fig. 3:**
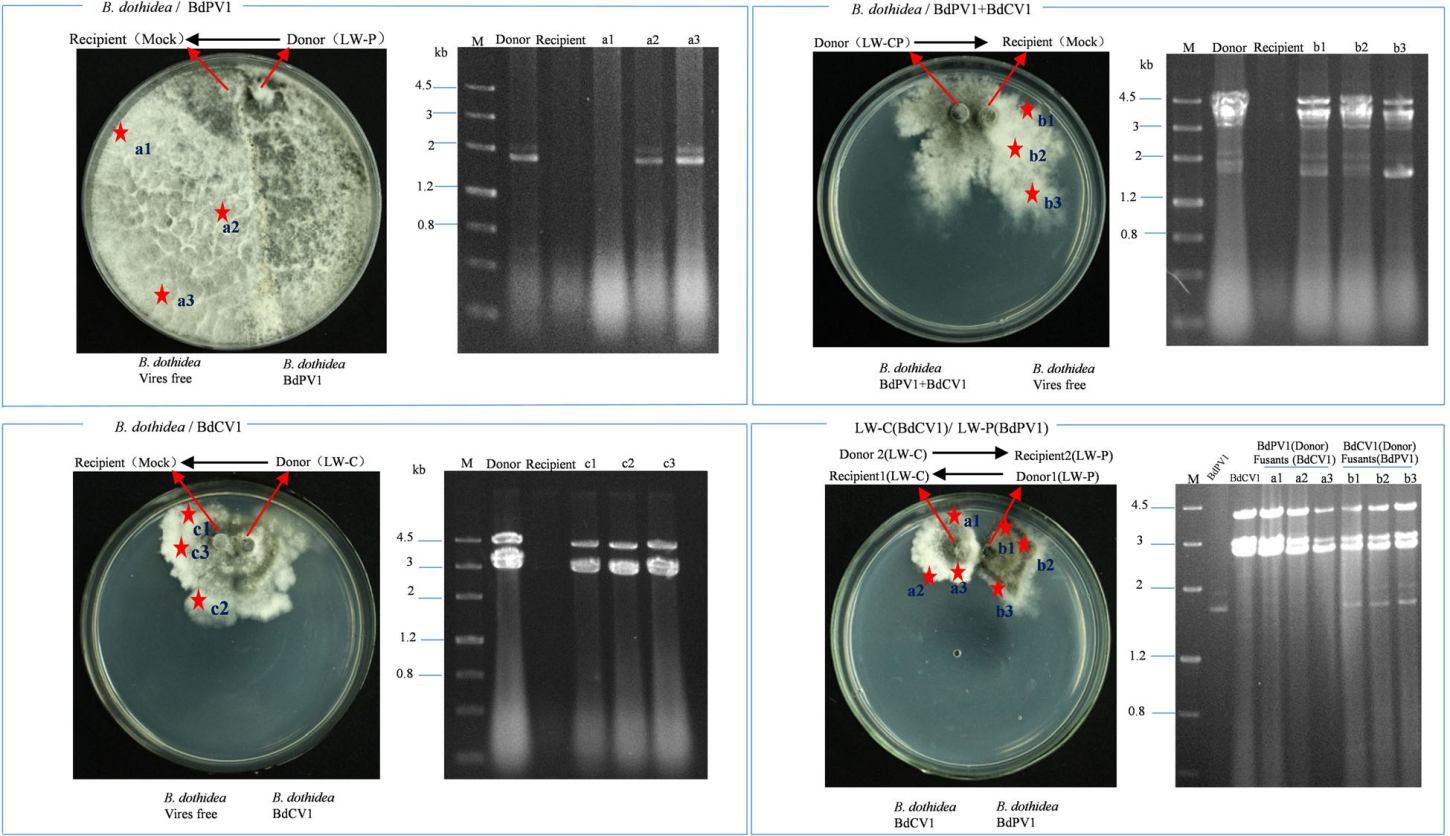
Horizontal transfer of mycovirus and dsRNA analysis of *Botryosphaeria dothidea* derivates. a1, a2, a3; b1, b2, b3; c1, c2, c3 represent derivates infected with mycovirus

BdCV1 and BdPV1 transmission to *B. dothidea* hosts was assessed by the detection of mycoviral dsRNA (Yang et al. 2017a). The results showed that BdCV1 and/or BdPV1 exist stably in LW-1 (109 of 109), LW-C (84 of 84) and LW-P (94 of 94) strains following vertical transmission of the offspring conidia from *B. dothidea* strains (Additional file 5: Figure S5). In addition, the horizontal transmission in co-cultures of LW-C/BdCV1 + LW-P/BdPV1 was also analyzed by detection of mycoviral dsRNA in the LW-C and LW-P strains. The results demonstrated that BdCV1 was transmitted to LW-P, whereas BdPV1 was not transmitted to LW-C (Fig. 3). In contrast, in the LW-CP/Mock, LW-C/Mock, and LW-P/Mock control groups, BdCV1 and/or BdPV1 were effectively transmitted to the Mock strain at higher frequencies than that reported previously (Wang et al. 2014, Wang et al. 2017b).

These results revealed that BdCV1 has a marked effect on the growth, development and pathogenic properties of *B. dothidea* during pear infection; however, the mechanisms underlying these effects remain to be clarified. To further understand the interactions between *B. dothidea* and BdCV1 and BdPV1, it is important to obtain a complete knowledge of the genome of *B. dothidea* LW-Hubei strain infecting the pear host.

### Sequencing and assembly of the B. dothidea LW-Hubei strain genome

**Fig. 4:**
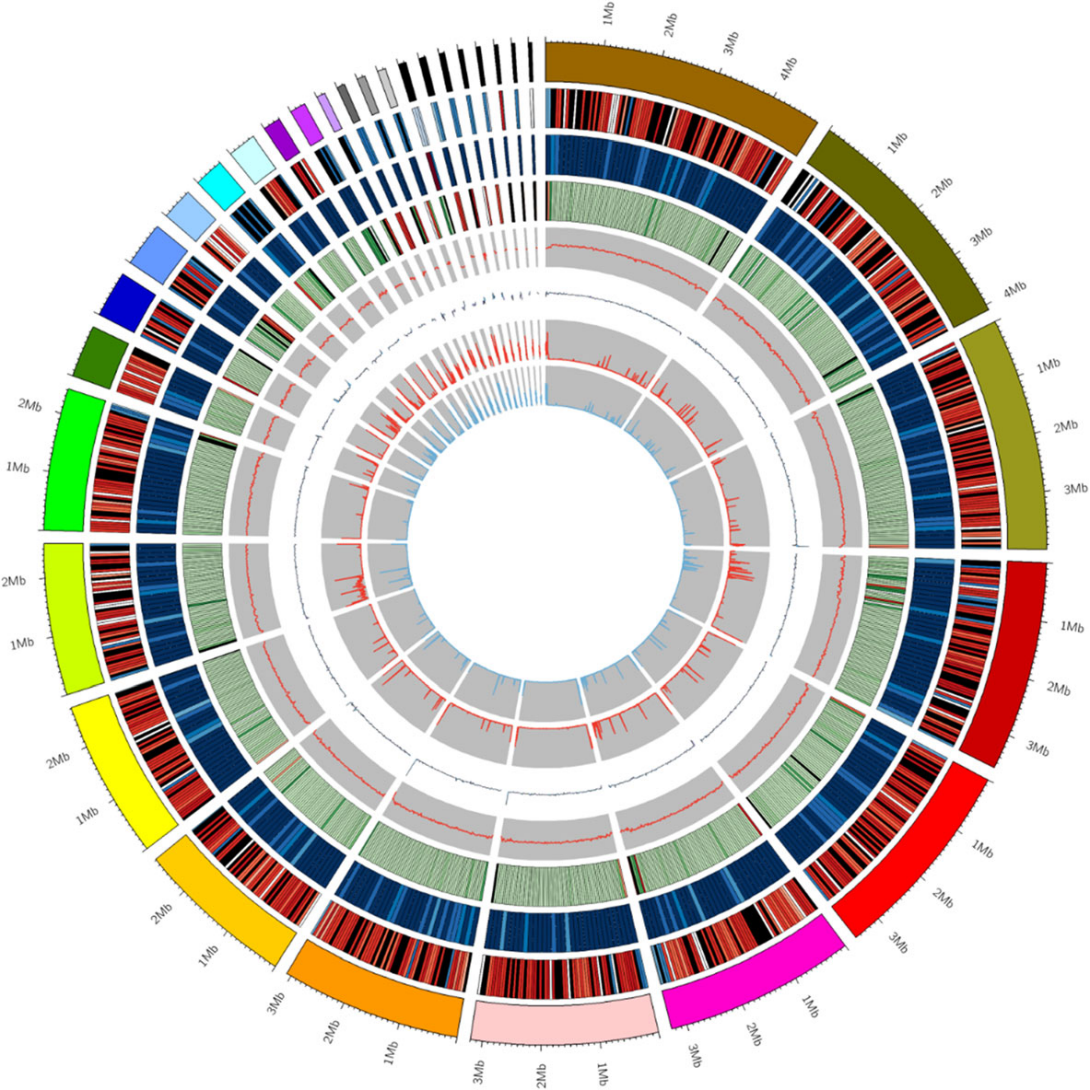
CIRCOS plots of the *Botryosphaeria dothidea* LW-Hubei genome. CIRCOS plots of the 49 scaffolds of *B. dothidea*, including 12 scaffolds (length > 2 Mb), are displayed in CIRCOS plots (Mb scale). The circus (from outside to inside) represent the following: 49 scaffolds (sorted by length), gene density (gene number in 50,000-bp non-overlapping windows), ncRNA density (ncRNA number in 100,000-bp non-overlapping windows), repeat coverage (repeat_coverage in 50,000-bp non-overlapping windows), GC (GC rate in 20,000-bp non-overlapping windows), GC_skew (GC skew in 20,000-bp non-overlapping windows), m4c and m6A methylations (100,000-bp non-overlapping windows)

To analyze the pathogenic mechanism by which *B. dothidea* infects pear, total genomic DNA from the mycelia of the *B. dothidea* LW-Hubei isolate was extracted and sequenced by the Single Molecule Real-Time (SMRT) technique in combination with the HiSeq platform. Three different sizes of insert libraries (270 bp, 10 kb and 20 kb) were sequenced to generate 6835 Mb trimmed data with a genome coverage of 147× for the Illumina and 3721 Mb (genome coverage of 80×) for the PacBio sequencing platform (Additional file 20: Table S4 and Additional file 21: Table S5). For the PacBio platform, subread distribution analyses confirmed the high quality of the 20-kb library (Additional file 6: Figure S6). Evaluation of the heterozygosity and distribution by k-mer revealed a volume peak at 30, indicating high homozygosity of the *B. dothidea* LW-Hubei isolate genome. In total, 68 contigs were generated with an N50 length of 2.72 Mb and 49 scaffolds with an N50 length of 3.27 Mb, including the 12 largest scaffolds with lengths > 2 Mb (40,021,009, accounting for 86.4% of the complete genome) displayed as a circos-plot (Table 1, Fig. 4). The total assembly size was approximately 46.34 Mb and 14,091 protein-coding genes were predicted, with a gene density of 304 genes per 1 Mb. In total, 465 non-coding RNAs (ncRNAs) were predicted, including 137 tRNAs (Table 1). Furthermore, 13,188 putative protein-coding genes were supported by mapping of the RNA-seq data to the obtained genome (Wang et al. 2018b), confirming the accuracy of the full genome assembly for the LW-Hubei strain (Additional file 22: Table S6). The average length of the predicted genes was 1684 bp, containing 3.1 exons (average length, 470 bp) and 2.1 introns (average length, 116 bp). The length of the coding regions of the predicted genes accounted for 43.89% of the total genome. The genomic features of the *B. dothidea* LW-Hubei isolates are shown in Table 1 and the genome structures are displayed in a circos-plot (Fig. 4). The complete genome has been deposited in the DDBJ/EMBL/GenBank databases under accession numbers SACU00000000. It is also worth noting that the complete genome of the *B. dothidea* LW-Hubei isolate is much larger than those of the previously reported isolates of *B. dothidea* LW030101 and *B. dothidea* Sxg01s that infect apple and grapevine, respectively, with genome sizes estimated at approximately 45.26 M and 43 M, respectively, based on assemblies of 125-bp libraries (Liu et al. 2016, Yan et al. 2018).

**Table 1:** Features of the *Botryosphaeria dothidea* LW-Hubei isolate genome

Features		*B. dothidea* LW-Hubei isolate
Assembly statistics	Size (Mb)	46.34
	HiSeq data (coverage)	6835 Mb (147×)
	PacBio data (coverage)	3721 Mb (80×)
	Total contig numbers (length)	68 (46,328,675 bp)
	Total scaffold number (length)	49 (46,346,907 bp)
	N50 contig (bp)	2,722,300
	N50 scaffold (bp)	3,265,504
	G + C content (%)	54.27
	Chromosome no.	0
Genome components	Protein-coding gene numbers (Length/genome length)	14,091 (51.21%)
	Average gene length (bp)	1684
	Gene density (#gene per Mb)	304
	Exons numbers (length/genome length)	43,305 (43.89%)
	Exons/gene	3.07
	Average exon length (bp)	469.68
	Intron numbers (length/genome length)	29,214 bp (7.33%)
	Introns/gene	2.07
	Average intron length (bp)	116.26
	ncRNA	465
	tRNA genes	137
	TEs and repeat size and % in genome	4.31 Mb and 9.3%

### Repetitive sequences and potential methylation sites

Repetitive sequences and transposable elements (TEs) increase the genome size and play vital roles in the genetic evolution and classification of fungal species (Bodega and Orlando 2014; Peter et al. 2016; Krishnan et al. 2018). A total of 4,309,902 bp (accounting for 9.3% of the genome) of repetitive sequences were identified in the *B. dothidea* LW-Hubei isolate genome, including DNA transposons, tandem repeat (TRs) sequences, long terminal repeats (LTR), short interspersed elements (SINE) and other unclassified transposons by using four types of prediction software. Interestingly, 94.9% (4,089,081 of 4,309,902) of the repetitive sequences were TEs, whereas the TRs accounted for only 5.1%. Notably, LTR retrotransposons and SINEs accounted for 52.2% (2,132,909 of 4,089,081) and 29.7% (1,216,468 of 4,089,081) of all TEs, respectively. In addition, DNA transposons accounted for a relatively low percentage of the sequence at 5.3% (216,254 of 4,089,081), and unknown TEs comprised 14.8% (604,268 of 4,089,081) (Table 2).

**Table 2:** The percentage of different types of repetitive elements in the *Botryosphaeria dothidea* LW-Hubei isolate genome

Repeat elements	Method	Type	DNA	LINE	LTR	SINE	unknown	Tandem Repeat	Total
TE	Repbase TEs	Length (bp)	124,634	528,773	906,058	1962	1856	–	1,546,903
		% of genome	0.2689	1.140	1.9549	0.0042	0.004	–	3.3377
	Protein Mask TEs	Length (bp)	159,623	931,118	1,525,603	0	0	–	2,613,914
		% of genome	0.3444	2.009	3.2917	0	0	–	5.6399
	De novo TEs	Length (bp)	82,280	1,122,469	2,001,497	0	602,412	–	3,796,845
		% of genome	0.1775	2.4219	4.3185	0	1.2998	–	8.1922
	Combined TEs	Length (bp)	216,254	1,216,468	2,132,909	1962	604,268	–	4,089,081
		% of genome	0.4666	2.6247	4.6021	0.0042	1.3038	–	8.8228
TR	TRF	Length (bp)	–	–	–	–	–	307,386	307,386
		% of genome	–	–	–	–	–	0.6632	0.6632
Repeat sequences	Total	Length (bp)	–	–	–	–	–		4,309,902
		% of genome	–	–	–	–	–		9.2992

Note:“-” indicate no data were obtained

It was recently reported that DNA methylation may exist in fungi (Luo et al. 2018). In total, 374,995 m4C (4-methyl-cytosine) residues, and 20,121 m6A (6-methyl-adenosine) were identified by SMRT analysis (Fig. 4). Moreover, most of the methylation sites (1,117,684) were unclassified. In accordance with the identified methylation positions, multiple motifs recognized specifically by transmethylases were detected (Additional file 23: Table S7).

### Comparative genomic analysis of B. dothidea LW-Hubei strains in Botryosphaerace

**Fig. 5:**
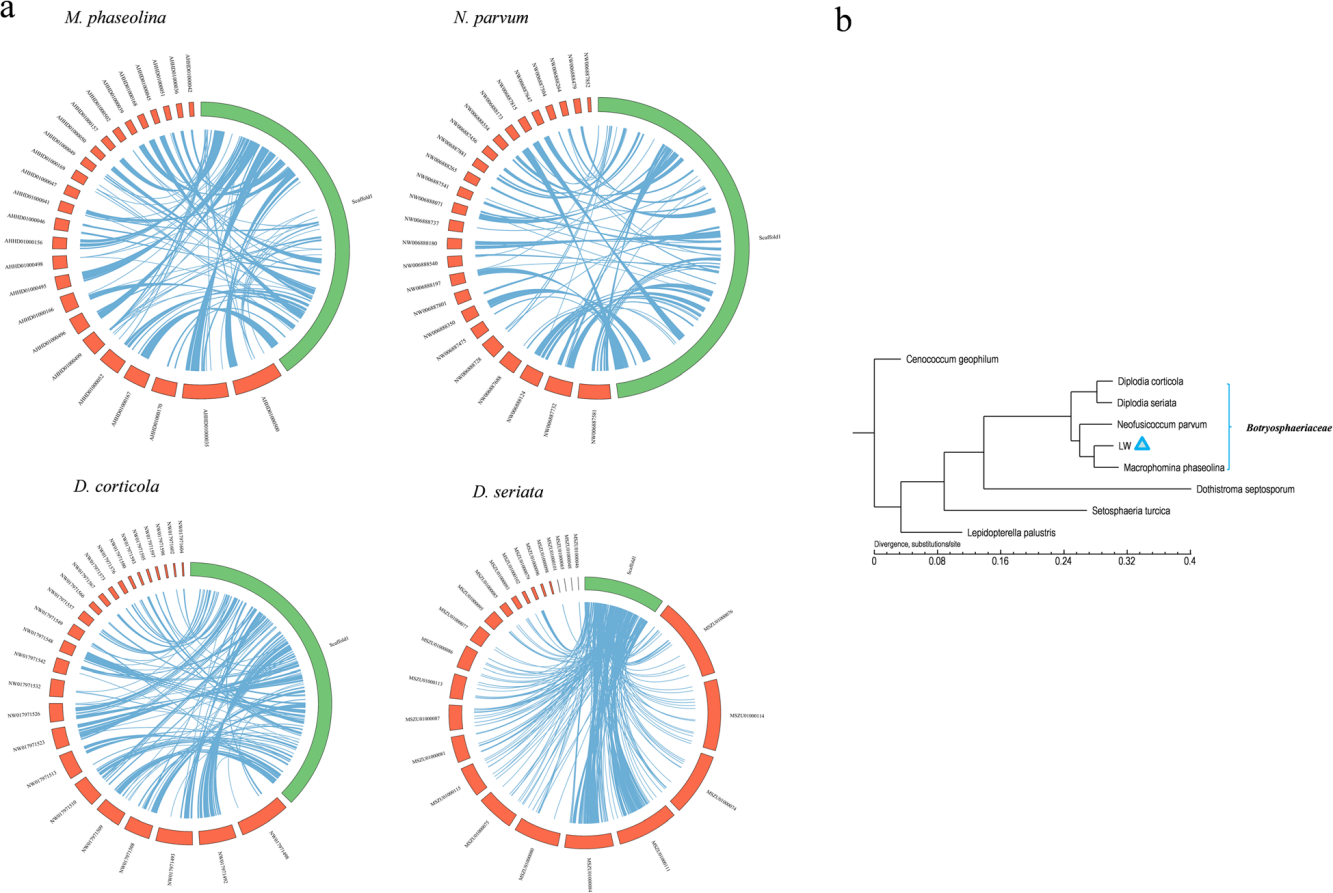
Synteny analysis and phylogenetic tree analysis of *Botryosphaeria dothidea* LW-Hubei with other fungal strains. **a** Synteny relationship between scaffold1 of *B. dothidea* LW-Hubei and that of *Macrophomina phaseolina, Neofusicoccum parvum, Diplodia corticola* and *Diplodia seriata* are indicated by connected lines; **b** Phylogenetic tree analysis based on core-pan genes from *B. dothidea* LW-Hubei and eight other fungi using the neighbor-joining method. The scale number represents branch lengths

Synteny analysis of the *B. dothidea* LW-Hubei isolate genome revealed that *B. dothidea* LW-Hubei exhibits a high degree of synteny with the other four *Botryosphaeriaceae* spp. genomes, *Macrophomina phaseolina*, *Neofusicoccum parvum*, *Diplodia seriata* and *Diplodia corticola* (Islam et al. 2012; Blanco-Ulate et al. 2013; van der Nest et al. 2014; Yan et al. 2018), at the nucleotide and amino acid levels, indicating sharing of the conserved core genes of *Botryosphaeriaceae* (Additional file 7: Figure S7). In detail, the scaffold1 sequences of the *B. dothidea* LW-Hubei isolate corresponded well with the corresponding scaffolds of *M. phaseolina*, *N. parvum*, *D. seriata* and *D. corticola* (Fig. 5a). Venn diagrams of the core-pan genes showed the existence of 3292 core genes in the five *Botrysphaerace* species, in addition to 4202 genes unique to the LW-Hubei isolate, 5294 for *M. phaseolina,* 3405 for *N. parvum,* 2384 for *D. corticola*, and 1412 for *D. seriata* (Additional file 8: Figure S8). Heatmaps of the dispensable genes in each strain showed the similarities and clusters of genes of the five species (Additional file 9: Figure S9).

Phylogenetic trees were constructed based on the similarities and differences in genotypes between species to reveal the relationships between fungal species in terms of genetic evolution. In addition to the reported sequences of four botryosphaeriaceous isolates, four *Dothideomycetes* spp. genomes were also included in comparative analyses (Additional file 24: Table S8). The sequences of the core-pan genes and sing-copy orthologs revealed that the LW-Hubei isolate is evolutionarily close to *M. phaseolina*, a plant pathogen that can cause stem bark sclerotia disease in jute plants (Fig. 5b). In addition, LW-Hubei is also evolutionarily close to the other three *Botrysphaerace* strains. Homologous proteins of LW-Hubei showed an average identity of 86.7, 83.3, 79.7, and 82.4% with those of *M. phaseolina*, *N. parvum, D. corticola* and *D. seriata*, respectively.

**Fig. 6:**
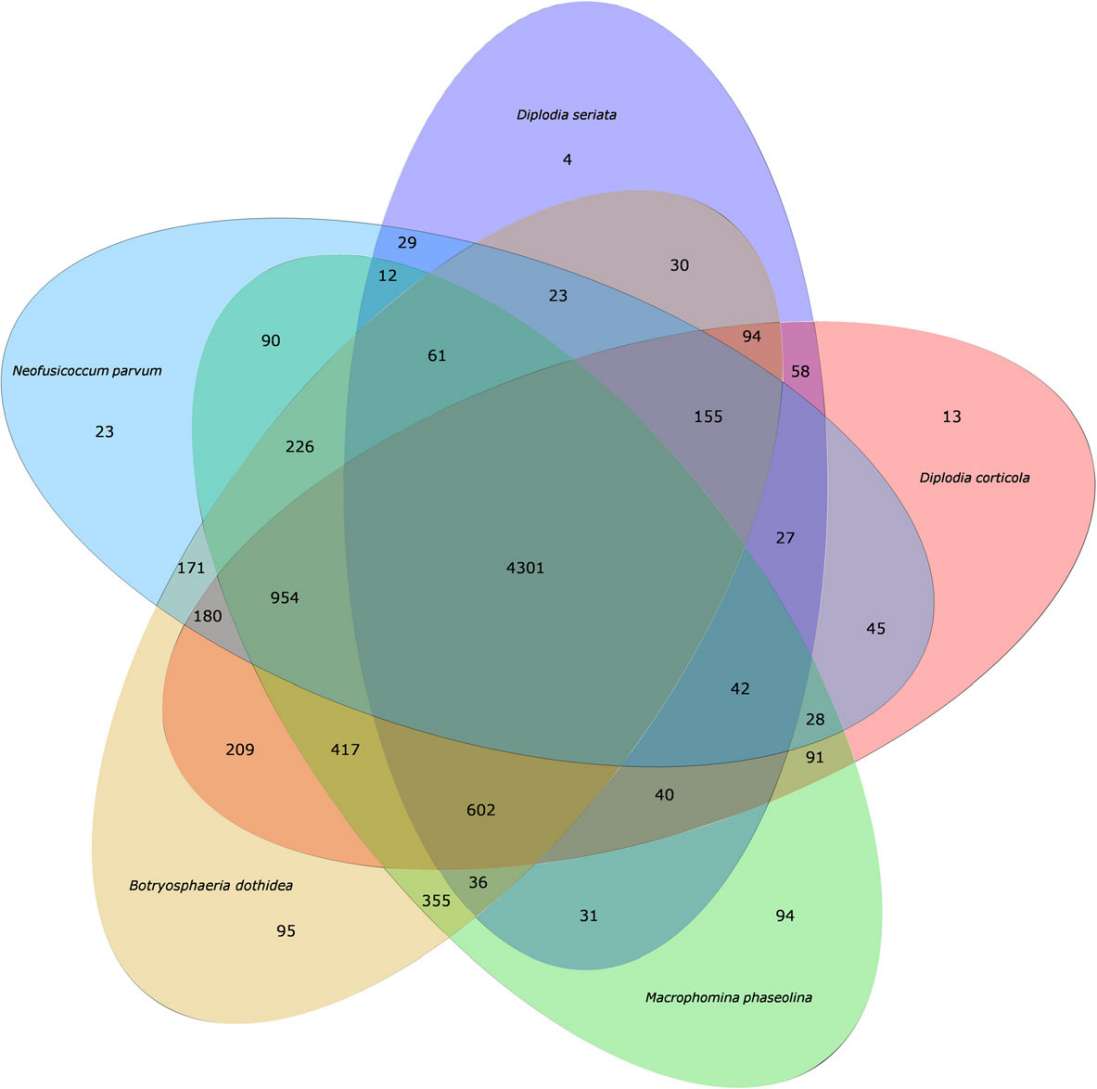
Venn diagram of predicted *Botryosphaeria dothidea* LW-Hubei gene families by comparison with four *Botryosphaeriaceae* species*.* The five fungi are represented by different colors: *B. dothidea* LW-Hubei strain, light pink; *Macrophomina phaseolina*, green; *Neofusicoccum parvum*, light blue; *Diplodia corticola*, pink and *Diplodia seriata*, blue

Gene families can be used to analyze the evolutionary history and differentiation paths of genes. Comparisons of the numbers of orthologs showed that LW-Hubei, *M. phaseolina, N. parvum, D. corticola* and *D. seriata* shared 4301 gene families, including 3139 single-copy orthologs, which were common to all five species. In total, 229 *Botryosphaeriaceae spp.*-specific groups were identified, consisting of 95 families unique to the LW-Hubei strain, 94 for *M. phaseolina,* 23 for *N. parvum*, 13 for *D. corticola* and four for *D. seriata* (Fig. 6). When compared with all other genomes, LW-Hubei had the highest orthologous (single-copy and multiple-copy orthologs) similarity to *M. phaseolina* (93.15%, 7334 of 7873), while sharing 90.98% (7163 of 7873), 85.84% (6758 of 7873) and 82.1% (6464 of 7873) gene orthologue similarities with *N. parvum*, *D. corticola* and *D. seriata*, respectively. The phylogenetic tree analysis showed that LW-Hubei is closely related to *M. phaseolina* based on gene family analysis (Additional file 10: Figure S10).

### Genome annotation of B. dothidea LW-Hubei isolate

A total of 14,091 protein-coding genes were predicted to exist in the genome of the *B. dothidea* LW-Hubei strain, covering approximately 43.9% of the genome sequence. Of these, 13,135 genes were annotated by comparisons with multiple databases. Functional annotation showed that 7558 (53.6%) genes were assigned with GO terms, most of which were involved in metabolic process, catalytic activity, binding, cell process and response to stress or stimulus. A further 4523 (32.1%) genes were mapped to the KEGG pathway database (Additional file 25: Table S9 and Additional file 11: Figure S11). The major functional groups included transcription factors (573 genes), pathogen-host interaction (PHI) (1096 genes) and 351 putative CAZymes, of which 79 (22%) potential CAZymes were annotated as PHI, indicating that a large group of PHI may be involved in LW-Hubei pathogenicity (Additional file 26: Table S10). The CAZy categories contained 59 auxiliary activities (AA), including 13 AA1 with degrading lactase activity, 85 carbohydrate binding modules (CBMs), 16 carbohydrate esterases (CEs), 131 glycoside hydrolases (GHs), 44 glycosyl transferases (GTs) and 16 polysaccharide lyases (PLs). Compared with the other four botryosphaeriaceous fungi*,* the *B. dothidea* LW-Hubei isolate contained a larger group of potential GHs, followed by CBMs, including CBM1, CBM12, CBM13, CBM18, CBM20, CBM21, CBM39, CBM42, CBM47, CBM48, CBM52 and CBM63. The numbers of CAZymes possessed by *B. dothidea* LW-Hubei was equal to that of *M. phaseolina* (362) (Islam et al. 2012).

Our analyses predicted 3833 secreted proteins. In addition, 12 putative effectors were annotated using the PHI database (Additional file 27: Table S11). The corresponding numbers of secreted proteins found in *M. phaseolina* (1863), *D. seriata* (910) and *N. parvum* (1097) were lower than that of the *B. dothidea* LW-Hubei isolate.

The *B. dothidea* genome encodes 552 transporter genes comprising 18 families. The majority of the transporter genes (133 of 552) were similar to those cataloged in the PHI-base. In the *B. dothidea* LW-Hubei genome, the largest proportion of transporters belonged to the major facilitator superfamily (MFS) family (83 genes), followed by the MC family (29), the NDH family (29), the ABC superfamily (27) and the amino acid-polyamine-organocation (APC) family (24) (Additional file 28: Table S12).

In addition, the *B. dothidea* genome encodes 128 kinase genes, consisting of AGC (13), atypical (14), CAMK (19), CK1 (2), CMGC (27), STE (18) and others (24) (Additional file 29: Table S13), which had orthologs in the PHI-base (82 of 128). This indicates that transporters and kinases in *B. dothidea* play a functional role in the pathogen-host interaction (Saier Jr et al. 2014; Cheng et al. 2015).

### The identification and expression levels of gene silencing components in B. dothidea strains during periods of infection with mycovirus

According to the functional annotation of the full genome in combination with de novo sequencing analysis reports (Wang et al. 2018b), several RNAi components were identified in the *B. dothidea* LW-Hubei genome, including two Dicers (DCL1 for GME1231_g and DCL2 for GME553_g), four argonautes (GME11353_g, GME6306_g, GME9732_g and GME8235_g) and three RNA-directed RNA polymerases (RdRp1 for GME8162_g, RdRp2 for GME11473_g, RdRp3 for GME9357_g).

**Fig. 7:**
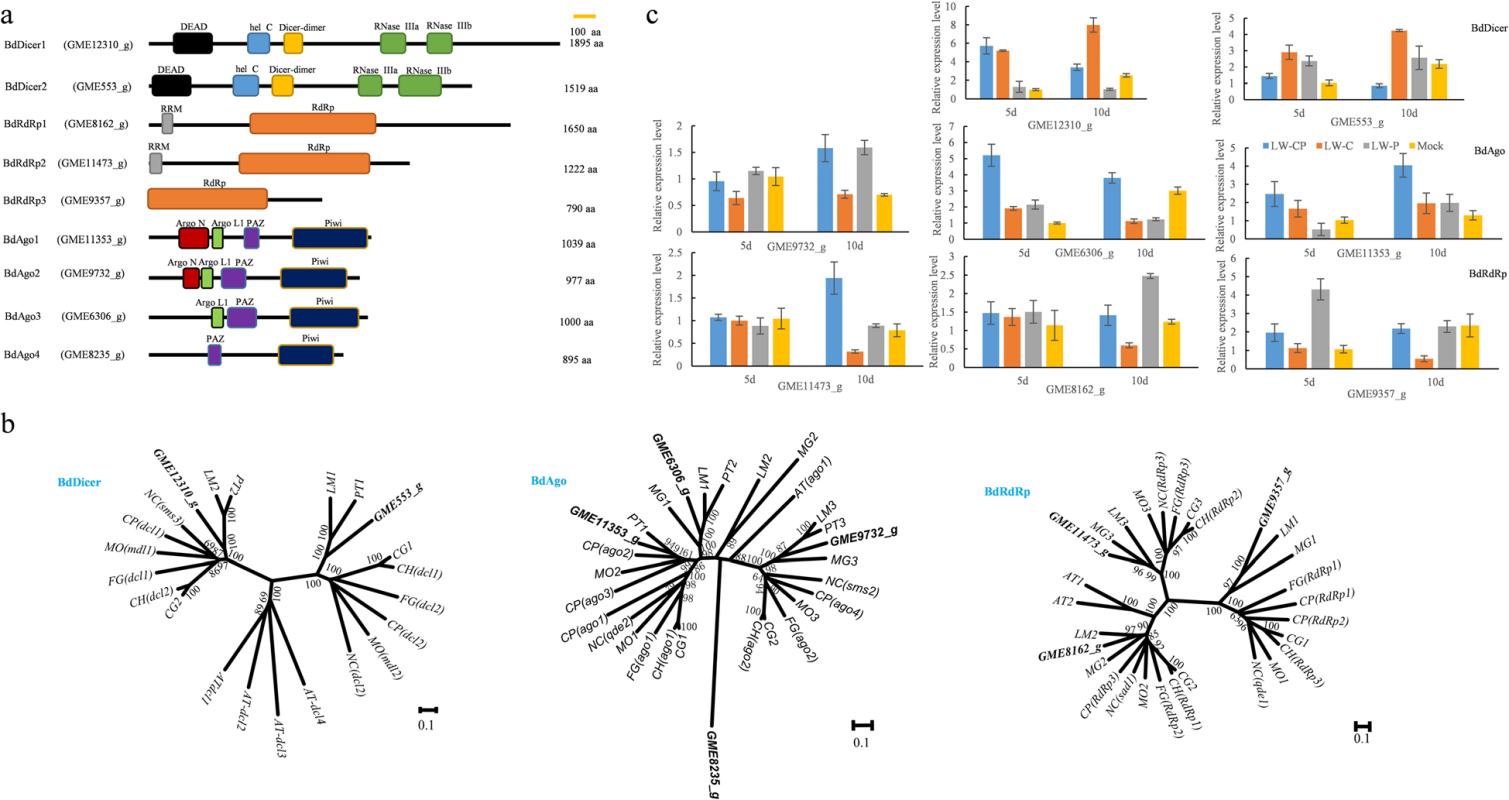
Sequence and expression analyses of the RNA-silencing components of *Botryosphaeria dothidea.* **a** Predicted domain structure of dicer (DCL), RNA-dependent RNA polymerases (RdRps) and argonaute (Ago) proteins in *B. dothidea* LW-Hubei strain. Lines represent the full length of proteins. Boxes represent the identified domains and are labeled with different colors. **b** Phylogenetic analyses of dicer (left), RdRps (right), and Ago (middle) protein sequences from *B. dothidea* and homologous proteins from representative members of other fungi. Counterparts from *Arabidopsis thaliana* served as the outgroup member. Rooted maximum likelihood neighbor-joining trees with 1000 bootstrap replicates were constructed by alignment of full-length protein sequences. The accession numbers for dicer, RdRps, and AGO protein sequences from representative members used in the alignment are listed in Additional file 30: Table S14. **c** Analysis of BdDicer, BdRdRp and BdAgo gene expression in mycovirus-infected *B. dothidea* LW-Hubei strains. Values were normalized to the mean *actin* gene (GME8592_g) expression

The BdDicer1 ORF encodes a protein of 1895 amino acids, and the size of the predicted protein encoded by BdDicer2 is 1519 amino acids. Both BdDicers possess a DEAD box, RNA helicase domain (helC), Dicer-dimerization domain and two RNaseIII catalytic domains. Amino acid sequence analysis revealed that three BdRdRps contained an RNA-dependent RNA polymerase domain (RdRp), which are highly conserved in fungi, whereas BdRdRp1 and BdRdRp2 possess an additional RNA recognition motif (RRM) in the N-terminal region. Three BdAgo proteins (GME11353_g, GME6306_g, GME9732_g) containing three conserved domains (Argo L1, PAZ and Piwi), and one BdAgo (GME8235_g) with two conserved domains (PAZ and Piwi) were identified among other argonaute orthologs (Fig. 7a).

To explore the molecular genetic evolution of the RNA-silencing pathway in Ascomycota, phylogenetic tree analysis of RdRp-, Dicer- and Ago-like proteins from *B. dothidea* LW-Hubei was performed at the amino acid level using the sequences of corresponding proteins in other fungal species from the Ascomycota clade as references (Additional file 30: Table S14). The BdDicers, BdRdRps and BdAgos clustered clearly with known members from other fungi with functions in meiotic silencing via the unpaired DNA (MSUD) or quelling pathways (Romano and Macino 1992; Fulci and Macino 2007; Li et al. 2010; Dang et al. 2011). The two BdDicer proteins were grouped into two clusters, whereas the four BdAgo proteins and three BdRdRp proteins were clustered into three groups. BdDicer2, BdRdRp3, and BdAgo1,3 clustered in a group with their counterparts from the quelling pathway, which were homologous to *Neurospora crassa* dcl2 (27.86% identity), qde1 (14.13%), and Qde2 (30.2 and 29.29%), respectively. BdDicer1, BdRdRp1, and BdAgo2 clustered with proteins from the MSUD pathway, which were homologous to *Neurospora crassa* sms3 (34.76% identity), sad1 (30.19%), and sms2 (32.71%), respectively. BdAgo4 was identified within a single group (Fig. 7b). BdRdRp2 was identified in a separate group with *N. crassa* RdRp3 (29.64% identity), the function of which is unknown. The expression level of BdRdRp2 (GME11473_g) in mycelia was elevated in response to BdPV1 infection of LW-P and LW-CP strains for 10 d (Fig. 7c).

The expression levels of RNAi components from *B. dothidea* LW-Hubei strains in response to BdCV1 and BdPV1 were determined by RT-qPCR. The results showed that the expression levels of BdDicer, BdAgo and BdRdRp proteins in mycelia were upregulated to some extent in response to BdCV1 and BdPV1 infection for 5 d and 10 d, respectively (Fig. 7c, Additional file 17: Table S1). Taken together, these results show that BdCV1 and BdPV1 infection of *B. dothidea* strains elicits the accumulation of BdDicers, BdAgos, and BdRdRps expression at the mRNA level, indicating that these genes are involved in the antiviral silencing defense pathway in *B. dothidea* following infection with mycovirus.

### Analysis of small RNAs of B. dothidea strains in response to dsRNA mycovirus infection

The expression of RNAi genes in *B. dothidea* led to the hypothesis that it possesses functional small RNAs (sRNAs) that trigger gene silencing. Four sRNA libraries were constructed from *B. dothidea* strains infected with mycovirus and a Mock (LW-Hubei strain) infected control and subjected to Illumina deep sequencing to investigate the existence and expression of sRNAs in *B. dothidea* strains; the raw sRNA sequencing data have been deposited in the NCBI sequence read archive (SRA) (http://www.ncbi.nlm.nih.gov/sra) under the accession numbers SRP174595 (BioProject Acc. no. PRJNA51169). Fungal-specific reads were identified by mapping to the obtained *B. dothidea* LW-Hubei genome. A total of 11,221,049, 11,837,018, 10,949,651 and 10,880,651 clean sRNAs reads were obtained from LW-CP, LW-C, LW-P and Mock, respectively. Annotation of the sequenced clean sRNAs, including intron, exon, repeats, rRNA, tRNA, snRNA, snoRNA, miRNA sequences and other unannotated reads are shown in Table 3. Comparison of sRNA clean reads from the three LW-CP/Mock, LW-C/Mock and LW-P/Mock comparisons libraries revealed great differences in reads among the LW-CP, LW-C, LW-P and Mock samples, although the expression of the common sRNA sequences is centralized as previously reported in plant and fungal hosts (Additional file 31: Table S15). Furthermore, these comparisons revealed the high level of consistency in the high-throughput sequencing data obtained for the four *B. dothidea* strains.

**Table 3:** The sRNA annotation of the Mock, LW-P, LW-C and LW-CP libraries constructed from the *Botryosphaeria dothidea* LW-Hubei strain

	Mock		LW-P		LW-C		LW-CP	
	Unique sRNA reads (%)	Total sRNA reads (%)	Unique sRNA reads (%)	Total sRNA reads (%)	Unique sRNA reads (%)	Total sRNA reads (%)	Unique sRNA reads (%)	Total sRNA reads (%)
Total	1,052,904 (100%)	10,880,651 (100%)	1,047,920 (100%)	10,949,651 (100%)	947,838 (100%)	11,837,018 (100%)	1,223,398 (100%)	11,221,049 (100%)
exon_antisense	7312 (0.69%)	13,835 (0.13%)	5689 (0.54%)	9833 (0.09%)	1184 (0.12%)	3168 (0.03%)	3037 (0.25%)	7638 (0.07%)
exon_sense	76,375 (7.25%)	95,458 (0.88%)	40,633 (3.88%)	48,843 (0.45%)	26,314 (2.78%)	32,399 (0.27%)	50,639 (4.14%)	61,294 (0.55%)
intron_antisense	3253 (0.31%)	13,331 (0.12%)	1635 (0.16%)	4160 (0.04%)	478 (0.05%)	2592 (0.02%)	925 (0.08%)	2793 (0.02%)
intron_sense	6753 (0.64%)	20,665 (0.19%)	3482 (0.33%)	24,179 (0.22%)	2379 (0.25%)	13,418 (0.11%)	4172 (0.34%)	34,456 (0.31%)
miRNA	13,276 (1.26%)	278,093 (2.56%)	14,568 (1.39%)	267,681 (2.44%)	16,364 (1.73%)	229,446 (1.94%)	16,098 (1.32%)	264,274 (2.36%)
rRNA	76,021 (7.22%)	527,959 (4.85%)	81,074 (7.7.4%)	532,555 (4.86%)	72,983 (7.70%)	599,461 (5.06%)	86,945 (7.11%)	826,302 (7.36%)
repeat	129,247 (12.28%)	4,444,540 (40.85%)	71,670 (6.84%)	898,130 (8.20%)	23,718 (2.50%)	98,207 (0.83%)	40,975 (3.35%)	218,227 (1.94%)
snRNA	638 (0.06%)	2020 (0.02%)	651 (0.06%)	2344 (0.02%)	537 (0.06%)	2608 (0.02%)	831 (0.07%)	3544 (0.03%)
snoRNA	296 (0.03%)	1787 (0.02%)	231 (0.02%)	1617 (0.01%)	274 (0.03%)	1619 (0.01%)	282 (0.02%)	1665 (0.01%)
tRNA	2963 (0.28%)	40,722 (0.37%)	3371 (0.32%)	74,633 (0.68%)	4866 (0.51%)	75,363 (0.64%)	4586 (0.37%)	129,076 (1.15%)
unannnation	736,770 (69.98%)	5,442,241 (50.02%)	824,916 (78.72%)	9,085,676 (82.98%)	798,741 (84.27%)	10,778,737 (91.06%)	1,014,908 (82.96%)	9,671,780 (86.19%)

**Fig. 8:**
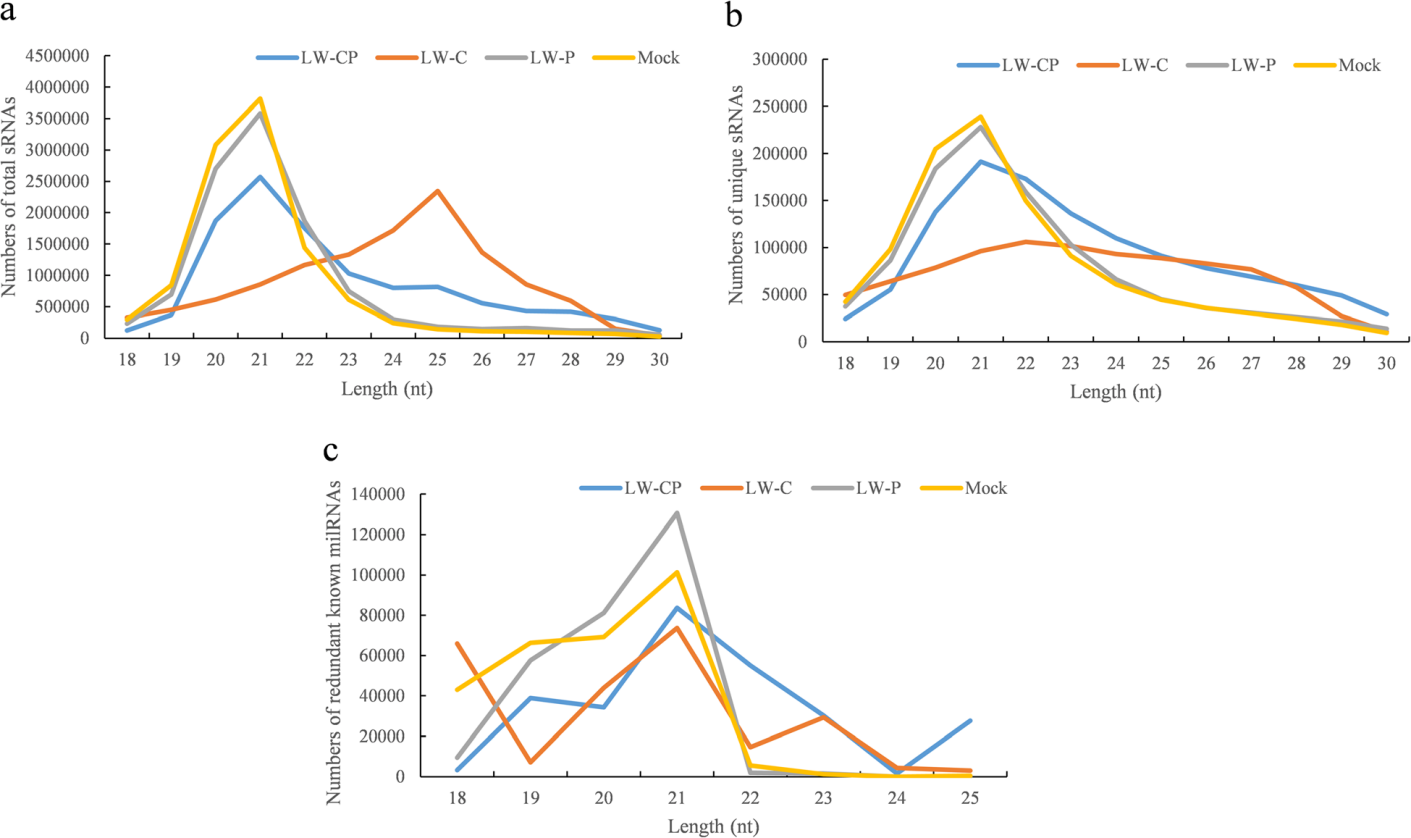
Length distribution of small RNAs (18–30 nucleotides) in the four libraries *Botryosphaeria dothidea* LW-Hubei strains. **a** Total small RNAs; **b** unique small RNAs; and **c** known milRNAs

*B. dothidea* miRNA-like RNAs (designated as *Bd-*milRNAs) accounted for 264,274 (2.36%), 222,946 (1.94%), 267,681 (2.44%) and 278,093 (2.56%) of the sequences in the LW-CP, LW-C, LW-P and Mock libraries, respectively (Table 3). Analysis of the length distribution revealed that sRNAs consisting of 21 nt were the most abundant, with 2,569,298 (191,333 unique reads), 3,582,770 (228,004 unique reads) and 3,816,137 (239,297 unique reads) in the LW-CP, LW-P and Mock libraries, respectively, while sRNAs consisting of 25 nt were the most abundant in LW-C, with 2,343,056 reads (88,608 unique reads). The next most abundant classes were the 20-nt and 22-nt sRNAs from the LW-CP, LW-P and Mock libraries, respectively, while the next most abundant class was 24-nt in LW-C (Fig. 8a). Among the unique sequences, the 21-nt sRNA sequences were most abundant, followed by the 20-nt sequences in the LW-P and Mock libraries, and the 22-nt sRNAs in the LW-CP library. The 22-nt sRNAs were the most abundant in the LW-C library (Fig. 8b).

Among the known *Bd-*milRNAs, the most abundant were 21-nt in length, with a total of 83,676, 73,641, 130,666 and 101,313 reads in the LW-CP, LW-C, LW-P and Mock libraries, respectively (Fig. 8c). Among the novel *Bd-*milRNAs, the most abundant were 21-nt in length, with a total of 209, 311, 1192 and 3623 reads in the LW-CP, LW-C, LW-P and Mock libraries, respectively. These data revealed inhibition of the *Bd-*milRNAs in the LW-CP and LW-C libraries (Fig. 8c). There were marked differences in the overall size distribution patterns of sRNAs between the libraries generated from the hypovirulent and virulent *B. dothidea* strains infected with mycoviruses, suggesting that BdCV1 and BdPV1 differentially affect sRNA and *Bd-*milRNAs accumulation in *B. dothidea.* In addition, these findings indicate that the proportion of reads from libraries of LW-CP, LW-C and LW-P, do not match the obtained target genome sequences at high proportion, mainly because of mycoviral infection of *B. dothidea* strains (Additional file 22: Table S6).

### Prediction novel Bd-milRNAs candidate in responsive to mycovirus

**Fig. 9:**
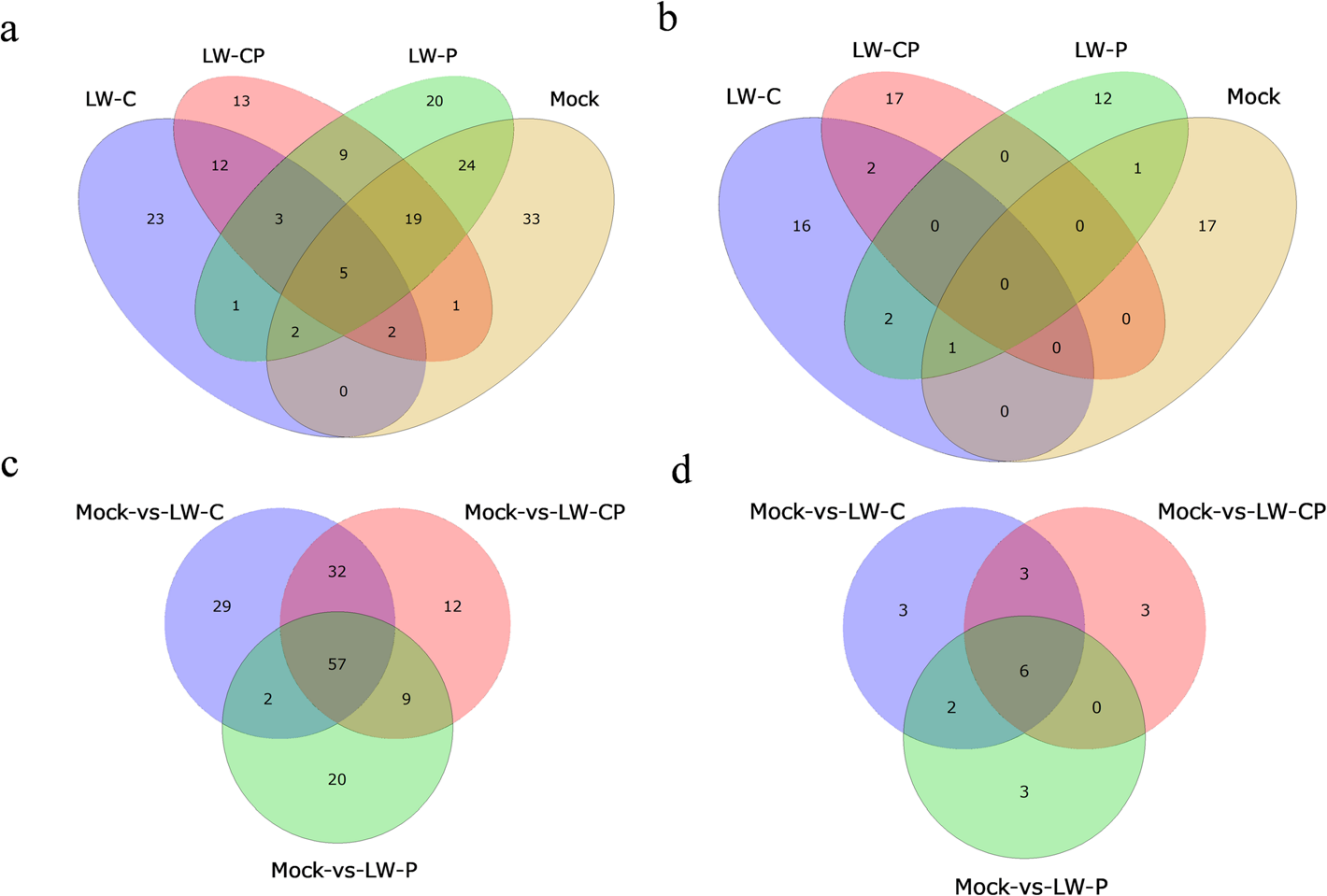
Venn diagrams illustrating the number of expressed milRNAs from mycovirus-infected *Botryosphaeria dothidea* LW-Hubei strains. **a** The unique and commonly expressed known milRNAs and **b** novel milRNAs in the four libraries (LW-CP, LW-C, LW-P and Mock). Illustration of the unique and commonly differentially expressed **c** known milRNAs and **d** novel milRNAs detected in three comparisons of LW-CP/Mock, LW-C/Mock and LW-P/Mock. Pair-wise interactions identified in comparisons between W-CP vs. LW-C, LW-CP vs. LW-P, LW-CP vs. Mock, and LW-C vs. LW-P libraries. Blue oval, LW-C; green oval, LW-P; light pink oval, Mock; pink oval, LW-CP

After removing the *Bd-*milRNAs with expression levels < 5 reads for further analysis of differential expression in *B. dothidea* strains, a total of 68 novel *Bd-*milRNAs candidate were predicted. Of these, 19, 21, 16 and 19 *Bd-*milRNAs were identified from the LW-CP, LW-C, LW-P and Mock libraries, respectively (Fig. 9b, Additional file 32: Table S16). The precursor sequences of these novel *Bd-*milRNA candidates were folded into a hairpin-like structure with precursor lengths ranging from 67 to 356 nt and minimal folding energy (MFE) ranging from − 21.4 to − 136.4 kal/mol (Additional file 32: Table S16 and Additional file 12: Figure S12). The base bias in the first position among the predicted novel *Bd*-milRNA candidates showed a preference for uridine (U) (Additional file 13: Figure S13). The sequencing and bioinformatics results showed lower expression levels of the novel *Bd*-milRNAs.

### Differential expression analysis of Bd-milRNAs from B. dothidea strains in response to mycoviral infection

The role of *Bd*-milRNAs in fungus-mycovirus interactions was investigated. A total of 167 known *Bd*-milRNAs were predicted, of which 64, 48, 83 and 86 were identified from the LW-CP, LW-C, LW-P and Mock libraries, respectively. Furthermore, 110, 120 and 88 known *Bd-*milRNAs were found to be differentially expressed in analysis of the three LW-CP/Mock, LW-C/Mock and LW-P/Mock comparison libraries, respectively (Fig. 9a, c). As expected, more changes in *Bd-*milRNA expression were observed in the analysis of the LW-CP/Mock and LW-C/Mock comparison libraries than those in the LW-P/Mock, revealing that BdCV1 has a greater effect than the BdPV1 on the expression of *Bd-*milRNAs. Evaluation of the LW-CP/Mock, LW-C/Mock and LW-P/Mock comparison libraries revealed common differential expression of 57 known *Bd*-milRNAs, while 12 known *Bd-*milRNAs were specifically expressed in the LW-CP/Mock library, 29 in the LW-C/Mock library and 20 in the LW-P/Mock library. In addition, 161 known *Bd-*milRNAs were differentially expressed, with the majority showing up-regulated expression in response to mycoviruses (Additional file 33: Table S17). Four randomly selected known *Bd-*milRNAs, *Bd*-milR8635, *Bd-*milR172c-3p, *Bd-*milR5636 and *Bd-*milR5568f-3p, were verified by stem-loop RT-PCR and sequencing analyses (Additional file 18: Table S2 and Additional file 34: Table S18).

Further, differential expression analysis of predicted novel *Bd-*milRNAs was performed. Twenty novel *Bd*-milRNAs candidates were expressed differentially, of which 12, 14 and 11 were significantly differentially expressed in the LW-CP/Mock, LW-C/Mock and LW-P/Mock libraries, respectively. Six novel *Bd-*milRNAs were commonly expressed differentially in the LW-CP/Mock, LW-C/Mock and LW-P/Mock libraries, while three novel *Bd-*milRNAs were specifically expressed in the LW-CP/Mock library, three in the LW-C/Mock library and three in the LW-P/Mock library (Fig. 9b, d). This revealed that specific and common alterations in *Bd-*milRNAs accumulation levels were induced in response to infection of *B. dothidea* strains with mycoviruses exhibiting mild and severe hypovirulence traits. The expression of two novel (*Bd-*milR6, *Bd-*milR12) and one known *Bd-*milRNA (*Bd-*milR1147.2) selected at random was verified by poly (A) tailing-mediated RT-PCR analysis, meanwhile u6 was as control (Additional file 18: Table S2 and Additional file 14: Figure S14).

### Temporal expression patterns of potential milRNA-mediated target genes in response to mycovirus infection of B. dothidea strains

Based on the *B. dothidea* LW-Hubei genome sequence, 690 targets were predicted as target genes for 81 known *Bd-*milRNAs from the LW-C/Mock library by both the psRobot and TargetFinder software, while 604 target genes for were predicted for 73 known *Bd-*milRNAs from the LW-CP/Mock library and 568 target genes for 62 known *Bd-*milRNA from the LW-P/Mock library. It was observed that many *Bd-*milRNAs regulated multiple mRNA targets from *B. dothidea* (Additional file 35: Table S19). In addition, using the psRobot and/or TargetFinder software, 35 target genes were predicted for 11 novel milRNAs from the LW-C/Mock library, 51 target genes for nine novel milRNAs from the LW-CP/Mock library and 25 target genes for nine novel milRNAs from the LW-P/Mock library (Additional file 36: Table S20). The targeted genes were annotated as TFs, transporters, kinases and effectors involved in the PHI, response to stimulus and signal transduction pathways (Additional file 35: Table S19 and Additional file 36: Table S20).

To confirm the ability of mycovirus-responsive *Bd-*milRNAs to regulate expression of their target genes during the period of infection, the expression levels of randomly selected *Bd-*milRNAs and putative target mRNAs were analyzed in *B. dothidea* strains cultured on PDA for 5 d. RT-qPCR was performed to analyze the temporal expression patterns of the following putative target mRNAs mediated by *Bd-*milRNAs: putative calcium-transporting p-type ATPase (GME3643_g) and hypothetical protein MPH_03910 (GME1913_g) as predicted targets of *Bd-*milR12; peptidase M43 pregnancy-associated plasma-A (GME4906_g) as a predicted target of *Bd-*milR33; putative polyketide synthase protein (GME2439_g) and hypothetical protein MPH_01630 (GME5002_g) as predicted targets of *Bd-*milR60; putative ABC transporter protein ATP-dependent bile acid permease (GME4334_g) and multicopper oxidase type 1 (GME2445_g) as predicted targets of *Bd-*milR1147.2; zinc finger C2H2-type protein (GME4116_g) as a predicted target of *Bd-*milR2094-5p; glucose-6-phosphate dehydrogenase (GME4518_g) as a predicted target of *Bd-*milR46; mannose-6-phosphate receptor binding protein (GME2361_g) as a predicted target of *Bd-*milR6; sugar/inositol transporter mfs sugar transporter (GME11656_g) as a predicted target of *Bd-*milR65; putative wd repeat domain 5b (GME3697_g) as a predicted target of *Bd-*milR172c-3p; urea carboxylase (GME1751_g) and putative ABC multidrug transporter mdr1 protein (GME4636_g) as predicted targets of *Bd-*milR8635; The cyclin-dependent protein kinase complex component PHO85 cyclin-6 (GME10476_g), hypothetical protein MPH_03537 (GME12854_g) and BdCV1 RdRp (GenBank Acc. no, KF688736) as predicted targets of *Bd-*milR5636; and BdCV1 CP (GenBank Acc. no, KF688737) as a predicted target of *Bd-*milR5568. The correlations between the expression levels of these *Bd-*milRNAs and their target mRNAs during mycelial growth for 5 d following BdCV1 and BdPV1 infection are shown in Table 4 The results revealed that the expression levels of partial target mRNAs exhibited the opposite expression patterns compared with those of the corresponding *Bd-*milRNAs in *B. dothidea* strains in response to mycovirus infection. In contrast, the expression patterns of partial putative target genes showed a positive correlation with those of the corresponding *Bd-*milRNAs (Table 4, Additional file 18: Table S2 and Additional file 15: Figure S15).

**Table 4:** The patterns of differentially expressed *Bd*-milRNAs and targeted gene mRNAs analyzed in mycelia from *Botryosphaeria dothidea* strains cultured for 5 d following infection with BdCV1 and BdPV1 obtained by sRNA sequencing and RT-qPCR analysis, respectively

ID *Bd*miRNA	Log_2_^FC^			ID target gene	RT- qPCR			Function annotation
	LW-CP/Mock	LW-C/Mock	LW-P/Mock		LW-CP/Mock	LW-C/Mock	LW-P/Mock	
*Bd*-milR12	/	10.22	/	GME3643_g	1.52	1.13	2.54	Putative calcium-transporting P-type ATPase
				GME1913_g	1.88	1.69	1.52	Hypothetical protein MPH_03910
*Bd*-milR33	8.55	/	/	GME4906_g	1.79	1.00	1.12	Peptidase M43 pregnancy-associated plasma-A
*Bd*-milR60	−7.01	−7.01	− 7.01	GME2439_g	0.44	0.76	2.04	Putative polyketide synthase protein
				GME5002_g	1.91	2.38	1.80	Hypothetical protein MPH_01630
				GME11323_g	0.04	0.35	0.03	Hypothetical protein UCDDS831_g08653
*Bd*-milR1147.2	/	15.973	/	GME2445_g	/	2.76	1.40	Multicopper oxidase type 1
				GME4334_g	1.11	0.93	1.50	Putative ABC transporter protein
*Bd*-miR2094-5p	−3.561	−15.79	−3.259	GME4116_g	6.16	3.15	0.68	Zinc finger C2H2-type protein
*Bd*-milR46	−7.37	−7.37	/	GME4518_g	2.36	3.87	0.62	Glucose-6-phosphate dehydrogenase
*Bd*-milR6	/	6.66	6.78	GME2361_g	1.87	1.75	1.21	Mannose-6-phosphate receptor binding protein
				GME985_g	0.64	1.01	0.28	Hypothetical protein MPH_12204
*Bd*-milR65	−7.72	−7.72	−7.72	GME11656_g	3.95	5.32	0.97	MFS sugar transporter
*Bd*-milR172c-3p	2.94	−14.26	2.68	GME3697_g	1.20	1.55	1.05	Putative WD repeat domain 5b
*Bd*-milR8635	−2.82	−5.34	/	GME1751_g	2.41	1.50	2.51	Urea carboxylase
				GME4636_g	1.16	0.86	0.45	Putative ABC multidrug transporter mdr1 protein
*Bd*-milR5636	/	14.92	/	GME10476_g	1.89	1.24	2.81	Cyclin-dependent protein kinase complex component
				GME1284_g	1.44	0.28	1.41	Hypothetical protein MPH_03537
				KF688736	8133.09	9243.67	–	Putative RNA-dependent RNA polymerase (*RdRp*) gene
*Bd*-milR5568	/	14.12	/	Unigene3269	128,971.41	11,426.09	47.78	Hypothetical protein PGUG_01202
				KF688737	2053.45	1537.81	–	Putative coat protein (*cp*) gene

Note: “/” indicates no differential expression for *Bd*-milRNA in library; “-” indicates no significant data

### Functional enrichment of putative milRNA-mediated target genes

**Fig. 10:**
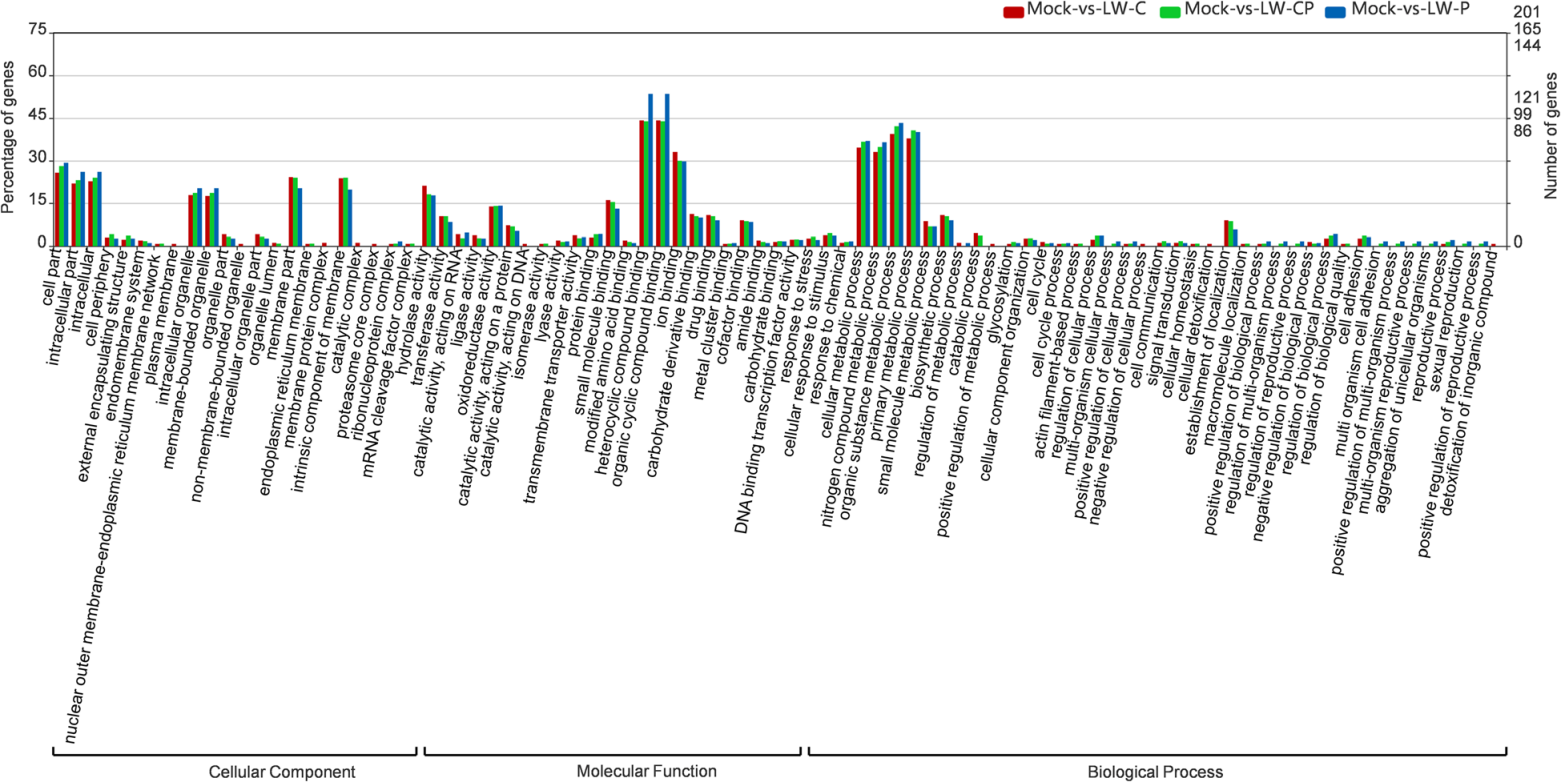
WEGO functional enrichment analysis of predicted *Bd*-milRNA target genes in mycovirus-infected *Botryosphaeria dothidea* LW-Hubei strains including of three comparisons between LW-CP vs. Mock (green), LW-C vs. Mock (red), and LW-P vs. Mock (blue) libraries, respectively. X axis represents GO term; Y axis represents number (percentage) of DEGs

The functional roles of the predicted milRNA target genes in the three comparison libraries were predicted based on analysis of the functional enrichment of all identified *Bd*-milRNA targets using WEGO (Web Gene Ontology Annotation Plot) software (Fig. 10). The predicted milRNA-regulated targets showed enrichment of GO terms in the biological process, cell process and molecular function categories, which is accordance with the predictions of de novo transcription analysis described previously (Wang et al. 2018b). In the biological process category, the predicted targets were involved in different biological processes, including cellular metabolic process, biosynthetic process, localization, catabolic process, cellular response to stimulus, regulation of cellular process, regulation of biological process, and signal transduction. In the cellular component category, the enriched GO terms included cell part, membrane-bounded organelle, membrane part, hydrolase activity and transferase activity. In the molecular function category, the enriched GO terms were involved in binding, cofactor binding, carbohydrate binding and response to stress. In addition, pathway enrichment analyses revealed spliceosome (ko03040), including 57 target genes with pathway annotation, was enriched to a greater level in *B. dothidea* strains in response to BdCV1 infection (Additional file 16: Figure S16).

## DISCUSSION

Among the economically important *Botryosphaeriae* species of fungi, *B. dothidea* causes severe warts and stem canker, as well as ring rot diseases of pear worldwide (Slippers and Wingfield 2007; Zhai et al. 2014). In this study, we investigated the potential pathogenic mechanism of *B. dothidea* LW-Hubei isolates on pear and the interactions of *B. dothidea* and mycovirus. To address these issues, we performed high-throughput genome sequencing in combination comparative genomics analysis of sRNAs, including *Bd-*milRNA/target mRNAs from *B. dothidea* strains infected with dsRNA mycoviruses.

Our investigations revealed that the complete genome sequence size of the *B. dothidea* LW-Hubei isolate is 46.34 Mb (Table 1). This is larger than the genome sizes of sequenced but not annotated *Botryosphaeriaceae* taxa *B. dothidea* and *N. parvum* isolates with similar genome sizes (42.6 Mb to 45.26 Mb), and low proportions of repeat sequences determined using the NGS method (Bodega and Orlando 2014; van der Nest et al. 2014; Liu et al. 2016; Luo et al. 2018; Yan et al. 2018). In particular, the assembled *B. dothidea* LW-Hubei isolate genome contains 4.31 Mb repetitive sequences, representing a relatively high proportion (9.41%) of the complete genome in *Botryosphaeriaceae* (Table 2, Fig. 4). The repeat sequences were randomly distributed across the full genome of *B. dothidea* LW-Hubei isolate. It was recently reported that DNA methylation may be involved in host cell processes, such as regulation gene transcription in fungal species (Luo et al. 2018; Plissonneau et al. 2018; Yan et al. 2018). The m6A and m4C in *B. dothidea* were predicted for the first time using SMRT, which revealed larger numbers of m4C in the genome, occurring at a frequency of 8082/Mb, while m6A was detected at a frequency of 434/Mb. However, the mechanism of m4C and m6A regulation in *B. dothidea* remains to be elucidated.

The comparative genome analysis revealed gene families and core-pan genes that are specific to *B. dothidea* LW-Hubei as well as those that are common to *Botryosphaeriaceae spp*., and are more closely related to *M. phaseolina* than to *D. corticola* and *D. seriata* (Figs. 5, and 6). Based on the genome assembly and annotation, we analyzed the specific CAZymes, effectors protein, kinase, and transporter proteins involved in *B. dothidea* infection of pear, and showed that there are slightly fewer than those found in other species of the *Botryosphaeriacea*e taxa (Islam et al. 2012; Fernandes et al. 2014; van der Nest et al. 2014; Liu et al. 2016; Wang et al. 2018b; Yan et al. 2018), although *B. dothidea* was found to possess a significant number of genes involved in PHI. Annotation of these genes revealed that the *B. dothidea* genome possesses a large number genes involved in the pathogenesis in pear hosts that are both specific and common to those of other pathogenic fungi. These findings significantly expand our understanding of the determinants of *B. dothidea* pathogenicity and host-pathogen interactions.

It is worth noting the existence of RNAi in the *B. dothidea* LW-Hubei strain. Genes encoding two homologs of Dicer (DCL1 and DCL2), four homologs of Argonaute, and three homologs of RdRp, were identified in the genome (Fig. 7). The phylogenetic trees indicate that BdDCL2, BdRdRp3, and BdAgo1,3 play roles in the RNAi pathway, whereas the combination of BdDCL1, BdRdRp1, and BdAgo2 might be required for the MSUD pathway in *B. dothidea* (Fig. 7b). In particular, RT-qPCR analysis showed that the expression of RNAi components was unregulated in response to BdCV1 and BdPV1 to some extent (Fig. 7c), which is also supported by the results of de novo transcriptome sequencing of *B. dothidea* strains (Wang et al. 2018b). It demonstrated that knock-out BdDicer2 gene led to reduced mycelia growth, virulence and BdPV1 cp and RdRp mRNA expression in transformants in comparison with the wild strain LW-P (data not published). It revealed that RNAi was related to regulation of the pathogenesis of *B. dothidea* and interaction with mycovirus. In addition, the horizontal transmission between the LW-C/BdCV1 and LW-P/BdPV1 strains of *B. dothidea* revealed that BdPV1 can not be transmitted from LW-P to LW-C, indicating that BdCV1 might induce gene silencing that leads to the resistance of LW-C to the transmission of BdPV1 (Fig. 3). Based on these results, we hypothesize that *B. dothidea* possesses a RNA-silencing system preferentially involving RNAi as part of the defense mechanism against mycovirus infection.

RNA-silencing involves the function of sRNAs as small regulatory molecules that further control endogenous and exogenous RNAs in mycoviruses and regulate biological processes (Zhang et al. 2008; Li et al. 2010; Dang et al. 2011; Huntzinger and Izaurralde 2011). The RNA-silencing components encoded by the *Dicer*, *Ago*, *RdRp* genes have been reported to influence sRNA biosynthesis. RNA-silencing involving ChDCL1 and ChAGO1 in *C. higginsianum* is proposed to function as an antiviral mechanism, and FgAGO-1 and FgDICER-2 is responsible for hairpin RNA-triggered RNA-silencing and related small interfering RNA accumulation in *F. graminearum* (Sun et al. 2009; Huntzinger and Izaurralde 2011; Nuss 2011; Chen et al. 2015; Campo et al. 2016). It can be speculated that a sophisticated RNA-silencing system that regulates sRNA production exists in *B. dothidea* strains.

It has been reported that milRNA expression profiles change during mycelium growth, conidiogenesis, and pathogenicity of fungi (Campo et al. 2016; Wang et al. 2016a, 2016b; Wang et al. 2017a, 2017c). sRNAs, especially miRNAs and siRNAs, are perturbed during mycovirus infection of fungi and have important influences on biological processes (Zhang et al. 2014; Campo et al. 2016; Wang et al. 2016a, 2016b). In this study, we revealed that BdCV1 has a greater effect on the biological characteristics of *B. dothidea* than BdPV1 (Figs. 1, 2 and 3, Additional file 1: Figure S1). Based on previous reports of milRNA-mediated gene silencing in fungi, we investigated the existence of milRNAs in *B. dothidea* by constructing and sequencing four sRNA libraries. Additionally, the genome data obtained provided a convenient resource for genome-scale investigation of *Bd-*milRNAs where context sequences around sRNA loci were needed for secondary structure predication. The results of the size distribution, position-specific nucleotide preference, and accumulation of specific sequences all suggest that *B. dothidea* possesses an endogenous sRNA biogenesis pathway. This is the first report of alteration in milRNAs in hypovirulent and virulent *B. dothidea* strains infected with mycovirus.

These bioinformatics and RT-qPCR analyses of changes in the expression of the *B. dothidea* transcriptome and milRNAs revealed that the expression of *Bd-*milRNAs was significantly altered by mycovirus infection, especially BdCV1, which has a great effect on the expression of *B. dothidea* sRNAs, including *Bd-*milRNA. These findings indicate that such dynamic sRNA (milRNA) profiles may have important functions in suppressing mycoviral infection (Table 3, Figs. 8 and 9). Furthermore, our results indicate the hypovirulent and non-hypovirulent phenotypes and the pathogenity of *B. dothidea* strains caused by mycovirus infection are related to the numbers of differentially expressed milRNAs and milRNA-regulated mRNAs (Wang et al. 2018b). Previous studies have demonstrated that miRNAs regulate key genes in disease resistance pathways to affect viral infections (Wang et al. 2016a, 2016b; Wang et al. 2017a, 2017c; Fu et al. 2017). In this study, we identified a number of predicted milRNA-target genes from *B. dothidea* that are possibly involved in disease resistance and defense. For instance, downregulation of the expression of an ABC transporter protein that was identified as a predicted target of *Bd-*milR1147 in LW-C infected with BdCV1 may inhibit the *B. dothidea* infection. In additions, expression of the MFS sugar transporter (GME11656_g) targeted by *Bd-*milR65 was up-regulated in *B. dothidea* in response to mycovirus infection. These transporters are important in the pathogenesis of fungi (Saier Jr et al. 2014); thus, our findings indicate the involvement of such transporters in the pathogenicity of *B. dothidea*-infecting mycoviruses. Glucose-6-phosphate dehydrogenase (GME4518_g) was upregulated in response to BdCV1 in the *B. dothidea* strains LW-C and LW-CP. Zinc finger C2H2-type protein (GME4116_g) as pathogenic factor was up-regulated, targeted by down-regulated expression *Bd-*milR2094-5p in response to BdCV1 and/or BdPV1 infection of *B. dothidea* strains, which may involve in the interaction of mycovirus and *B. dothidea* (Table 4).

In addition, BdCV1 RdRp and CP were predicted as targets of *Bd-*milRNA5636 and *Bd-*milRNA5568, respectively. It has been reported that gga-miR-130b-3p plays a crucial role in host defense against IBDV (Infectious Bursal Disease Virus) infection by targeting the viral itself genome (Cheng et al. 2015; Fu et al. 2017); thus, in accordance with this, it can be speculated that *Bd-*milRNAs may be directly involved in the interaction of mycovirus with the *B. dothidea* host. WEGO analysis revealed that the predicted *Bd-*milRNA-targeted genes were enriched for kinases, small secreted proteins and transporters (Fig. 10). The majority of these targets have roles in stress-related gene regulation or PHI pathways, suggesting that milRNA-mediated regulation of the transcriptome is an essential process in the mycovirus-stress response in the *B. dothidea* host. The mechanistic details of this process in resistance of *B. dothidea* to BdPV1 and BdCV1 remain to be elucidated.

## CONCLUSIONS

In this study, we sequenced the 46.34 M genome of the *B. dothidea* LW-Hubei strain and annotated 13,135 genes to reveal the pathogenic mechanism. We identified RNAi components and mycovirus-altered milRNA expression patterns in the *B. dothidea* host and showed that BdCV1 has more effect on milRNA expression than BdPV1. This information not only contributes to a more comprehensive understanding of the complex processes involved in the regulation of the biological characteristics of *B. dothidea*, but also provides insights into the molecular mechanisms of hypovirulence of *B. dothidea* in pear following mycovirus infection. Our analysis revealed that the interaction of *B. dothidea* and mycovirus involves the combined action of the antiviral gene silencing pathway, and milRNA-mediated regulation of target gene mRNA expression in *B. dothidea*.

## Additional files

Additional file 1: **Figure S1.** Evaluation of the pathogenicity of *Botryosphaeria dothidea* strains. (**a**) Lesions on ‘Hohsui’ pear fruit and branches, and ‘Fushi’ apple fruit induced by *Botryosphaeria dothidea* strains; (**b**) Lesion length on ‘Hohsui’ pear fruit at 9 d (b-I), ‘Fuji’ apple fruit at 4 d (b-II) and ‘Hohsui’ pear branches at 20 d (b-III) after inoculation with LW-1(LW-CP), LW-C, LW-P, Mock, HL-1 and HBWH-1 *B. dothidea* strains. (DOCX 653 kb)

Additional file 2: **Figure S2.** The colony morphology of mycovirus-infected *Botryosphaeria dothidea* strains in MS culture at 25 °C darkness for 3 d (I) and developing conidiomata and conidial angle under black light with 365 nm wavelength for 5 d with the naked eye (II) and 11 d observed under stereo microscope (III). Scale bars = 0.5 mm. (DOCX 2450 kb)

Additional file 3: **Figure S3.** Conidiogenous cells with developing conidia and mature conidia from *B. dothidea* strains cultured on PDA culture. Scale bars = 20 μm. (DOC 1051 kb)

Additional file 4: **Figure S4.** The mycelial morphology of *Botryosphaeria dothidea* strains cultured on PDA for 3 d at 25 °C in continuous darkness observed under a light microscope. Scale bar = 10 μm. a, b, c and d represent LW-CP, LW-C, LW-P and Mock samples, respectively. (DOCX 614 kb)

Additional file 5: **Figure S5.** The detection of dsRNA patterns from the offspring condia of (a) LW-CP, (b) LW-C and (c) LW-P by 1.2% agarose gel electrophoresis. (DOCX 826 kb)

Additional file 6: **Figure S6.** Statistical analysis of high quality subreads with estimated genome coverage 80 X were obtained from LW-Hubei genome sequencing data. (DOCX 48 kb)

Additional file 7: **Figure S7.** Whole genome synteny analysis of LW-Hubei strain by comparison with *Macrophomina phascolina* MS6, *Neofusicoccum parvum* UCRNP2, *Diplodia corticola* CBS 112549 and *Dothistroma septosporum* NZE10 at the nucleotide (nt) level. (a) The 49 scaffolds of LW-Hubei and *Macrophomina phascolina* MS6 were compared (Mb scale). (b) The 49 scaffolds of LW-Hubei and *Neofusicoccum parvum* UCRNP2 were compared (Mb scale). (c) The 49 scaffolds of LW-Hubei and *Diplodia corticola* CBS 112549 were compared (Mb scale). (d) The 49 scaffolds of LW-Hubei and *Diplodia seriata* were compared (Mb scale). (DOCX 3136 kb)

Additional file 8: **Figure S8.** Venn diagram of the common and unique Core-pan genes among five *Botryosphaeriaceae* strains: *Botryosphaeria dothidea* LW-Hubei, *M. phaseolina* MS6, *N. parvum* UCRNP2*, D. corticola* CBS 112549 and *D. seriata. (DOCX 114 kb)*

Additional file 9: **Figure S9.** Heatmap of the dispensable gene in five *Botryosphaeriaceae* strains*. (DOCX 69 kb)*

Additional file 10: **Figure S10.** Phylogenetic tree analysis based on gene families from *Botryosphaeria dothidea* LW-Hubei and the eight reference fungi by the neighbor-joining method. The scale number represents branch lengths. (DOCX 65 kb)

Additional file 11: **Figure S11.** Annotation of the LW-Hubei genome by Nr and KOG databases. (a) Annotation of 12,273 proteins by the Nr database. Large percentage of genes in LW-Hubei genome with homologs in *Macrophomina phascolina*. (b) Annotation of 2536 proteins by the KOG database for delineation of 25 Clusters of Orthologous Groups of proteins (COG). The majority of proteins (*n* = 292) were classified into General function prediction only. (DOCX 141 kb)

Additional file 12: **Figure S12.** The precursors of five novel milRNAs and their hairpin structures in *Botryosphaeria dothidea* strains. The mature *Bd*-milRNAs are shown in yellow and milRNA*s are underlined in green. The numbers show the base locations. (PDF 203 kb)

Additional file 13: **Figure S13.** First nucleotide bias in novel *Bd*-milRNA candidates isolated from *Botryosphaeria dothidea* strains in (a) Mock, (b) LW-C, (c) LW-P and (d) LW-CP libraries. (DOCX 215 kb)

Additional file 14: **Figure S14.** Putative *Bd*-milRNAs (two novel and one known) detected by poly (A) RT-PCR. (DOCX 233 kb)

Additional file 15: **Figure S15.** Expression levels of *Bd*-milRNA target mRNAs detected by RT-qPCR. (PDF 282 kb)

Additional file 16: **Figure S16.** KEGG pathway classifications of functional enrichment for differentially expressed known (a) and novel (b) *Bd*-milRNA target mRNAs in mycovirus-infected *Botryosphaeria dothidea* strains of LW-CP, LW-C and LW-P. (PDF 125 kb)

Additional file 17: **Table S1.** Sequences of qPCR primers used for analysis of the expression level of RNAi components in *Botryosphaeria dothidea* strains. (DOCX 14 kb)

Additional file 18: **Table S2.** The sequences of primers used for analysis of the expression of putative *Bd*-milRNAs and target gene mRNAs in *Botryosphaeria dothidea* strains. (DOCX 16 kb)

Additional file 19: **Table S3.** The sizes of conidia from *Botryosphaeria dothidea* strains. (DOCX 13 kb)

Additional file 20: **Table S4.** Summary of original sequencing data for *Botryosphaeria dothidea* LW-Hubei isolate generated using the Illumina platform. (DOCX 13 kb)

Additional file 21: **Table S5.** Summary of original sequencing data for *Botryosphaeria dothidea* LW-Hubei isolate generated using the PacBio platform. (DOCX 14 kb)

Additional file 22: **Table S6.** Statistical analysis of basic RNA-seq and small RNA sequencing information corresponding to the LW-Hubei strain genome used in this study. (DOCX 15 kb)

Additional file 23: **Table S7.** Statistical analysis of specific motifs for transmethylases. (DOCX 19 kb)

Additional file 24: **Table S8.** The complete genome features of the eight fungi used as references in the comparative genome analyses. (DOCX 14 kb)

Additional file 25: **Table S9.** Different methods used for prediction of functional annotation of *Botryosphaeria dothidea* LW-Hubei isolate. (DOCX 13 kb)

Additional file 26: **Table S10.** Annotation of the CAZy categories, PHI and TF in LW-Hubei isolate. (XLSX 149 kb)

Additional file 27: **Table S11.** Predicted effectors (*n* = 12) from *Botryosphaeria dothidea* LW-Hubei can be annotated by PHI. (DOCX 15 kb)

Additional file 28: **Table S12.** Annotated transporters (*n* = 552) of *Botryosphaeria dothidea* LW-Hubei isolate. (DOCX 13 kb)

Additional file 29: **Table S13.** Predicted kinases (*n* = 128) from *Botryosphaeria dothidea* LW-Hubei isolate. (DOCX 14 kb)

Additional file 30: **Table S14.** Summary of accession numbers for RNAi components from the fungal strains analyzed in this study. (DOCX 15 kb)

Additional file 31: **Table S15.** Summary of common and specific sRNA sequences and mean frequencies in the (a) LW-CP and Mock libraries, (b) LW-P and Mock libraries, and (c) LW-C and Mock libraries constructed from *Botryosphaeria dothidea* strains. (DOCX 15 kb)

Additional file 32: **Table S16.** The 62 candidate novel *Bd*-milRNAs and six novel *Bd*-milRNAs among the milRNA*s detected in *Botryosphaeria dothidea* strains. (DOCX 32 kb)

Additional file 33: **Table S17.** The differentially expressed known *Bd*-milRNAs in the LW-CP/Mock, LW-C/Mock and LW-P/Mock libraries constructed from *Botryosphaeria dothidea* strains by sRNA sequencing. (DOCX 29 kb)

Additional file 34: **Table S18.** Three known *Bd*-milRNAs sequences were cloned and sequenced by stem-loop RT-PCR (DOCX 50 kb)

Additional file 35: **Table S19.** The target gene of known *Bd*-milRNAs predicted by psRobot and/or TargetFinder software. (XLSX 575 kb)

Additional file 36: **Table S20.** The target gene of novel *Bd*-milRNAs predicted by psRobot and/or TargetFinder software. (XLSX 20 kb)

## Abbreviations

Ago: Argonaute
*B. dothidea* miRNA-like RNAs: *Bd-*milRNAs
BdCV1: *Botryosphaeria dothidea* chrysovirus 1
BdPV1: *Botryosphaeria dothidea* partitivirus 1
CAZy: Carbohydrate-active enzymes
COG: Cluster of Orthologous Groups
CTAB: Cetyltrimethyl ammonium bromide
PHI: Pathogen-host interaction
RdRp: RNA-dependent RNA polymerase
RT: Reverse transcriptase
SMRT: Single Molecule Real-Time
TEM: transmission electron microscopy
WEGO: Web Gene Ontology Annotation Plot
