# Supplementary material for: Comprehensive analysis of full genome sequence and Bd-milRNA/target mRNAs to discover the mechanism of hypovirulence in Botryosphaeria dothidea strains on pear infection with BdCV1 and BdPV1
Source: IMA Fungus. 2019 Jun 7;10:3. doi: 10.1186/s43008-019-0008-4 (PMC7325678; doi:10.1186/s43008-019-0008-4)
Supplement: Supplementary file 1 — Figure S1. Evaluation of the pathogenicity of Botryosphaeria dothidea strains. (a) Lesions on ‘Hohsui’ pear fruit and branches, and ‘Fushi’ apple fruit induced by Botryosphaeria dothidea strains; (b) Lesion length on ‘Hohsui’ pear fruit at 9 d (b-I), ‘Fuji’ apple fruit at 4 d (b-II) and ‘Hohsui’ pear branches at 20 d (b-III) after inoculation with LW-1(LW-CP), LW-C, LW-P, Mock, HL-1 and HBWH-1 B. dothidea strains. (DOCX 653 kb) [file 43008_2019_8_MOESM1_ESM.docx]

Additional file 8: **Figure S1** Evaluation of the pathogenicity of *Botryosphaeria dothidea* strains. **(a)** Lesions on ‘Hohsui’ pear fruit and branches, and ‘Fuji’ apple fruit induced by *Botryosphaeria dothidea* strains; **(b)** Lesion length on ‘Hohsui’ pear fruit at 9 d (b-I), ‘Fuji’ apple fruit at 4 d (b-II) and ‘Hohsui’ pear branches at 20 d (b-III) after inoculation with LW-1(LW-CP), LW-C, LW-P, Mock, HL-1 and HBWH-1 *B. dothidea* strains.

‘Hohsui’ pear stem

‘Fuji’ apple

‘Hohsui’ pear

CK

HBWH-1

HL-1

Mock

LW-P

LW-C

LW-CP


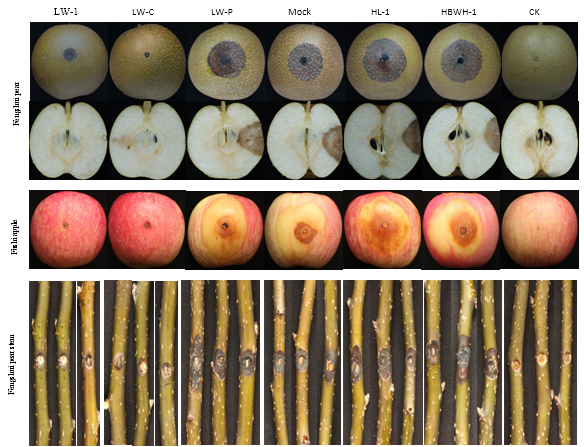


a

b


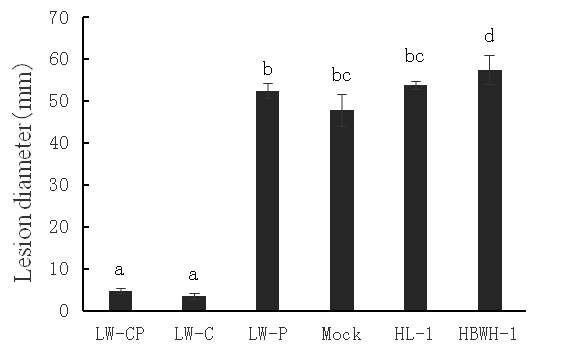

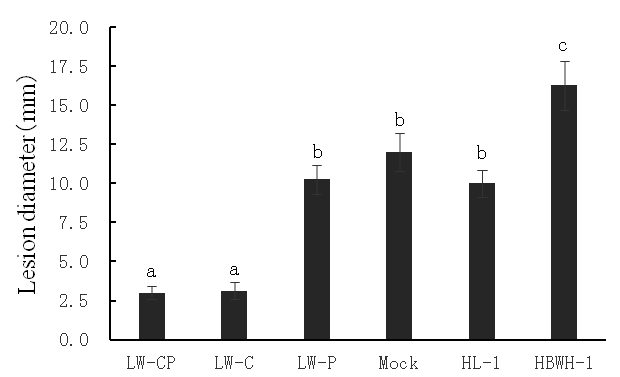

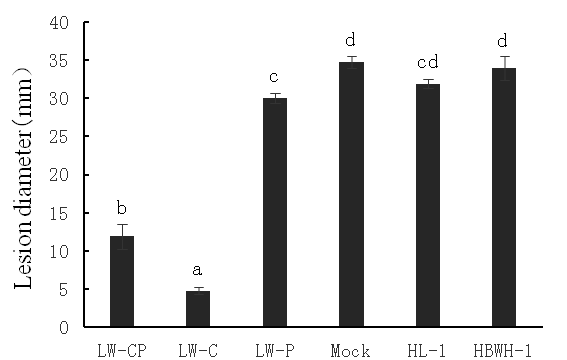


IIII III

II

I
