# Supplementary material for: Comprehensive analysis of full genome sequence and Bd-milRNA/target mRNAs to discover the mechanism of hypovirulence in Botryosphaeria dothidea strains on pear infection with BdCV1 and BdPV1
Source: IMA Fungus. 2019 Jun 7;10:3. doi: 10.1186/s43008-019-0008-4 (PMC7325678; doi:10.1186/s43008-019-0008-4)
Supplement: Supplementary file 2 — Figure S2. The colony morphology of mycovirus-infected Botryosphaeria dothidea strains in MS culture at 25 °C darkness for 3 d (I) and developing conidiomata and conidial angle under black light with 365 nm wavelength for 5 d with the naked eye (II) and 11 d observed under stereo microscope (III). Scale bars = 0.5 mm. (DOCX 2450 kb) [file 43008_2019_8_MOESM2_ESM.docx]

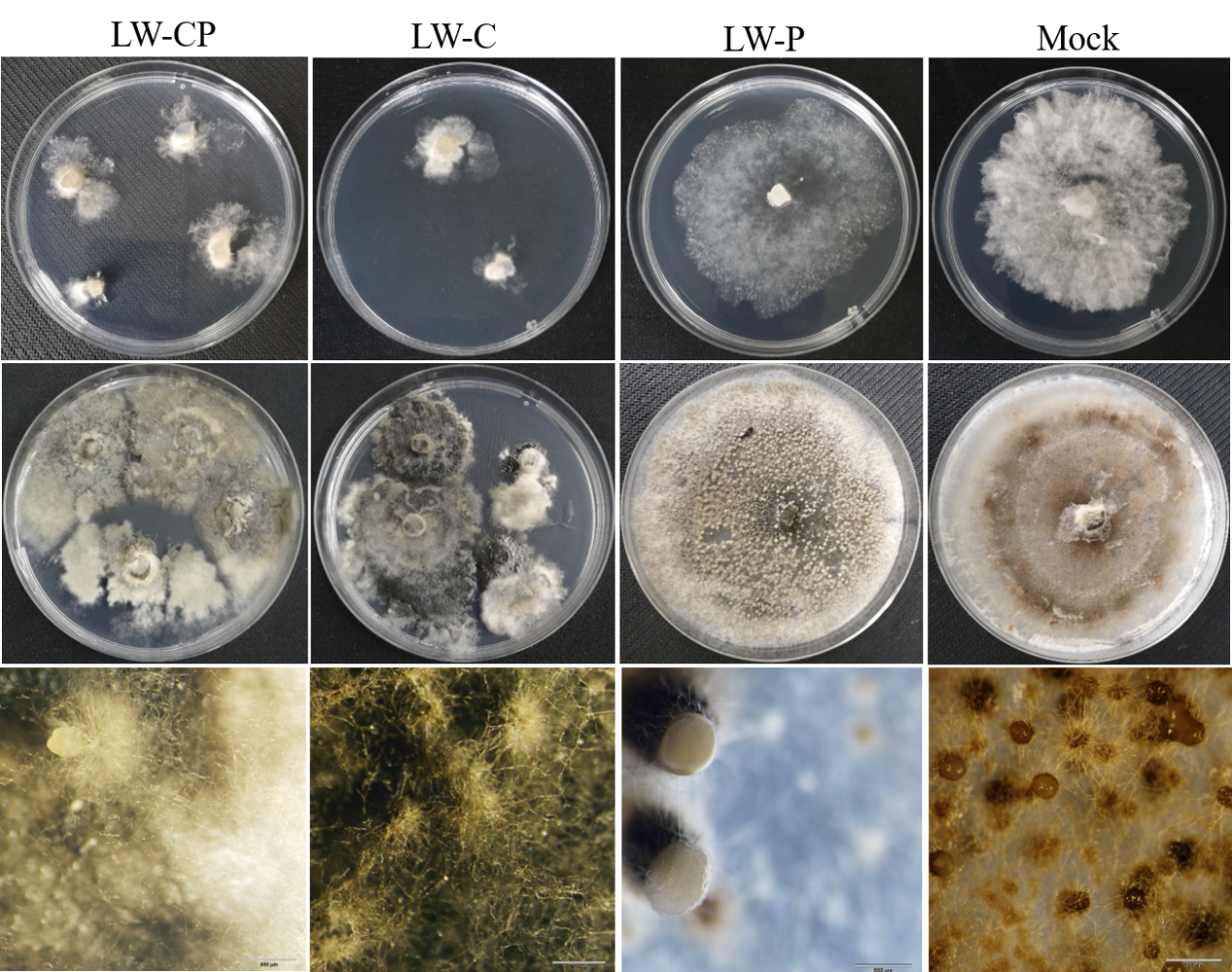
Additional file 2: **Figure S2** The colony morphology of mycovirus-infected *Botryosphaeria dothidea* strains in MS culture at 25℃ darkness for 3 d (I) and developing conidiomata and conidial angle under black light with 365 nm wavelength for 5 d with the naked eye (II) and 11 d observed under stereo microscope (III). Scale bars = 0.5mm.

III

II

I
