# Supplementary material for: Comprehensive analysis of full genome sequence and Bd-milRNA/target mRNAs to discover the mechanism of hypovirulence in Botryosphaeria dothidea strains on pear infection with BdCV1 and BdPV1
Source: IMA Fungus. 2019 Jun 7;10:3. doi: 10.1186/s43008-019-0008-4 (PMC7325678; doi:10.1186/s43008-019-0008-4)
Supplement: Supplementary file 3 — Figure S3. Conidiogenous cells with developing conidia and mature conidia from B. dothidea strains cultured on PDA culture. Scale bars = 20 μm. (DOC 1051 kb) [file 43008_2019_8_MOESM3_ESM.doc]

Additional file 3: **Figure S3** Conidiogenous cells with developing conidia and mature conidia from *B. dothidea* strains cultured on PDA culture. Scale bars = 20 μm.


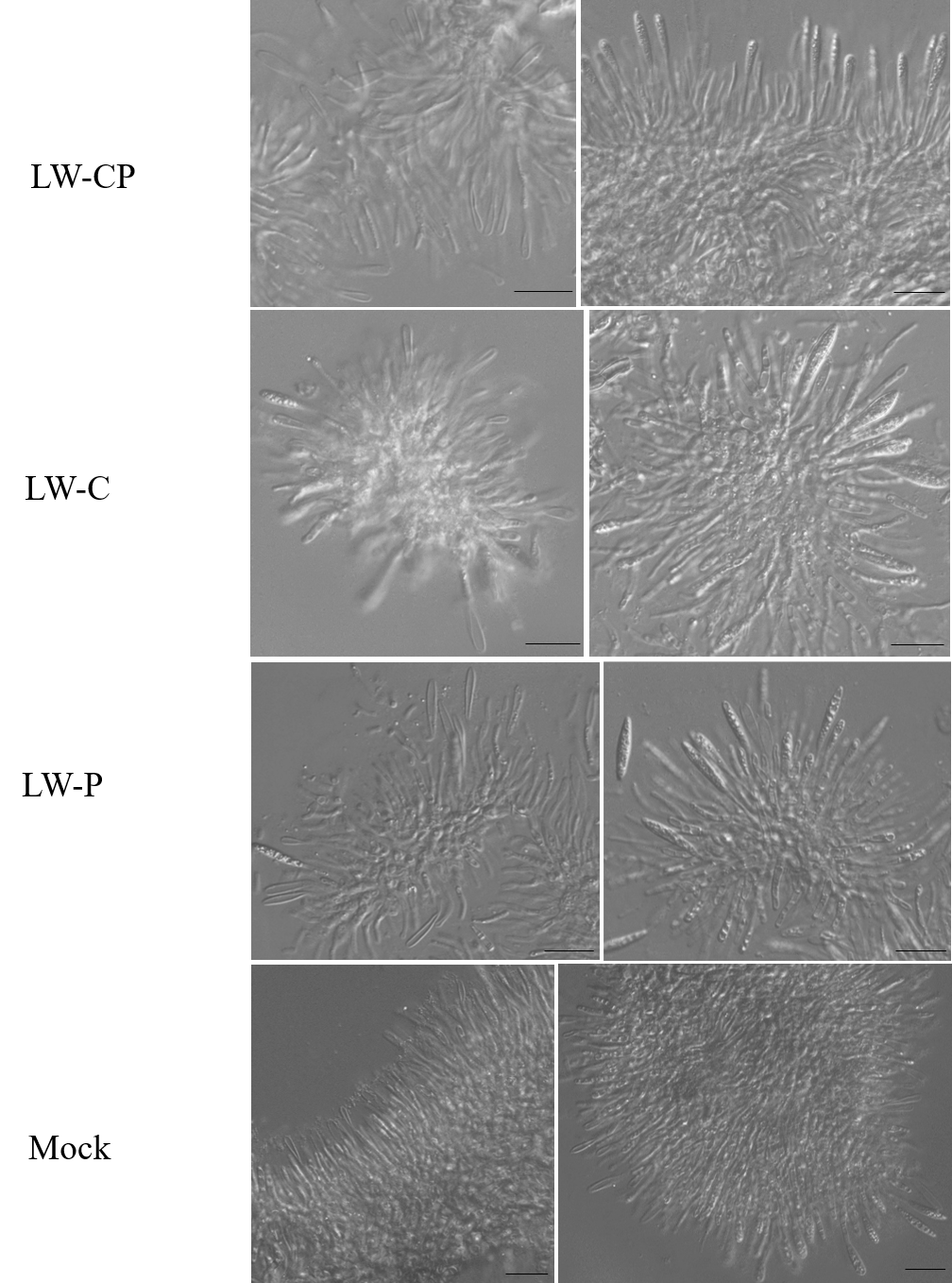
k

LW-CP

LW-C

LW-P

Mock
