# Supplementary material for: Comprehensive analysis of full genome sequence and Bd-milRNA/target mRNAs to discover the mechanism of hypovirulence in Botryosphaeria dothidea strains on pear infection with BdCV1 and BdPV1
Source: IMA Fungus. 2019 Jun 7;10:3. doi: 10.1186/s43008-019-0008-4 (PMC7325678; doi:10.1186/s43008-019-0008-4)

Additional file 4: **Figure S4** The mycelial morphology of *Botryosphaeria dothidea* strains cultured on PDA for 3 d at 25℃ in continuous darkness observed under a light microscope. Scale bar = 10 μm. a, b, c and d represent LW-CP, LW-C, LW-P and Mock samples, respectively.

a

b

d

c


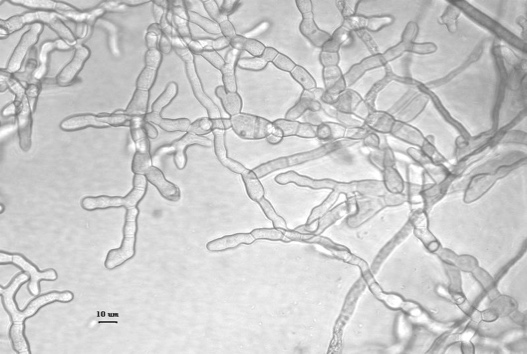

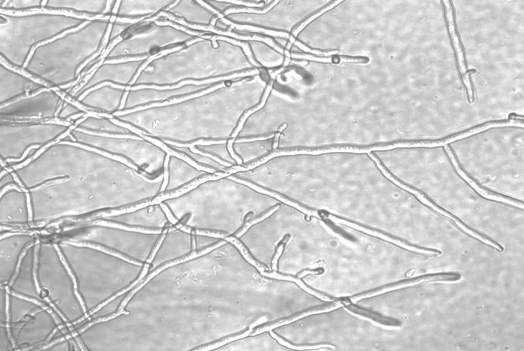

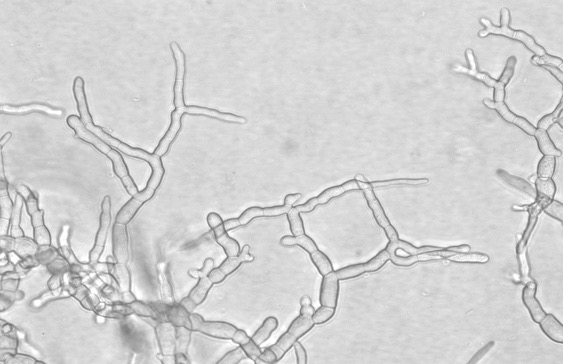

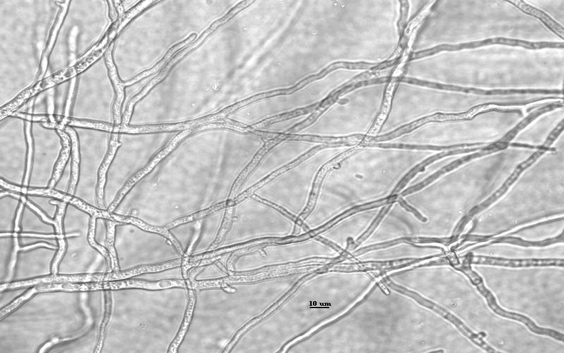

Supplement: Supplementary file 4 — Figure S4. The mycelial morphology of Botryosphaeria dothidea strains cultured on PDA for 3 d at 25 °C in continuous darkness observed under a light microscope. Scale bar = 10 μm. a, b, c and d represent LW-CP, LW-C, LW-P and Mock samples, respectively. (DOCX 614 kb) [file 43008_2019_8_MOESM4_ESM.docx]
