# Supplementary material for: Comprehensive analysis of full genome sequence and Bd-milRNA/target mRNAs to discover the mechanism of hypovirulence in Botryosphaeria dothidea strains on pear infection with BdCV1 and BdPV1
Source: IMA Fungus. 2019 Jun 7;10:3. doi: 10.1186/s43008-019-0008-4 (PMC7325678; doi:10.1186/s43008-019-0008-4)
Supplement: Supplementary file 5 — Figure S5. The detection of dsRNA patterns from the offspring condia of (a) LW-CP, (b) LW-C and (c) LW-P by 1.2% agarose gel electrophoresis. (DOCX 826 kb) [file 43008_2019_8_MOESM5_ESM.docx]

Additional file 5: **Figure S5** The detection of dsRNA patterns from the offspring condia of (a) LW-CP, (b) LW-C and (c) LW-P by 1.2% agarose gel electrophoresis.

BdCV1

BdPV1

2500

1000

4500

3000

2000

1200

4500

3000

2000

1200


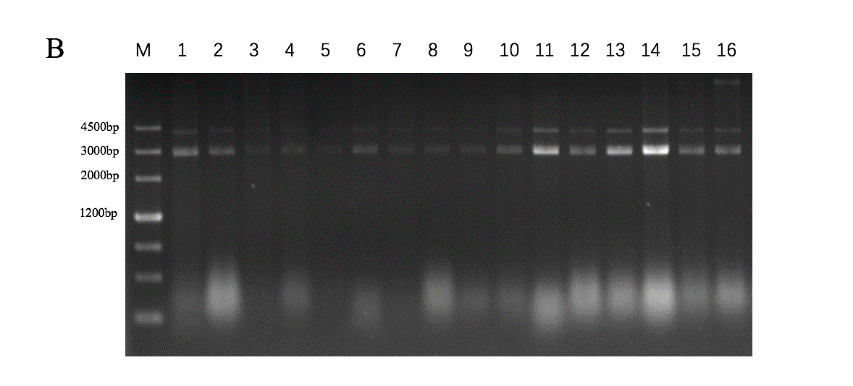

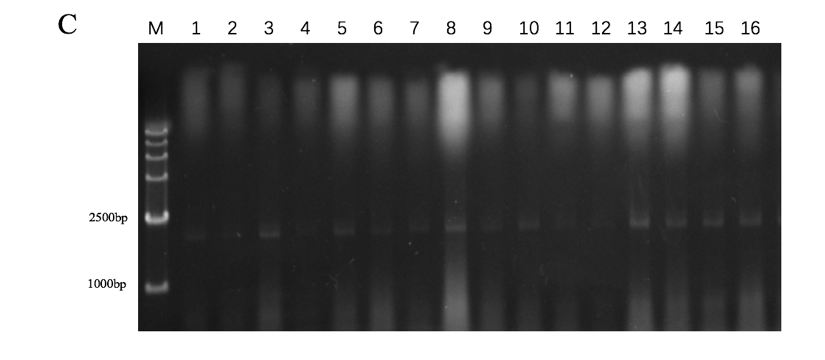

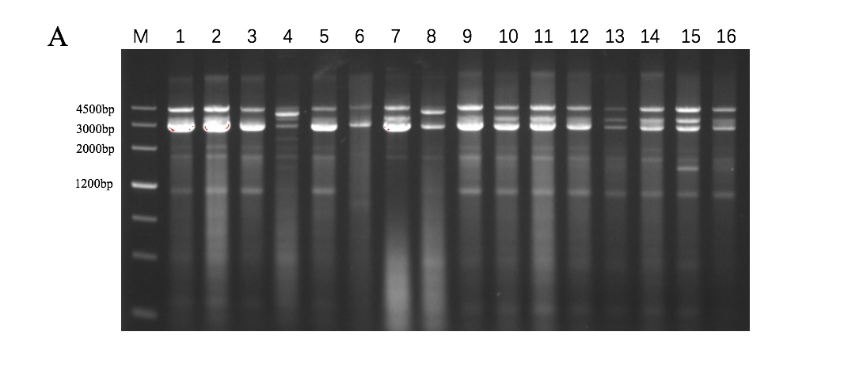


**a**

**b**

**c**

BdCV1

BdPV1
