# Supplementary material for: Comprehensive analysis of full genome sequence and Bd-milRNA/target mRNAs to discover the mechanism of hypovirulence in Botryosphaeria dothidea strains on pear infection with BdCV1 and BdPV1
Source: IMA Fungus. 2019 Jun 7;10:3. doi: 10.1186/s43008-019-0008-4 (PMC7325678; doi:10.1186/s43008-019-0008-4)
Supplement: Supplementary file 6 — Figure S6. Statistical analysis of high quality subreads with estimated genome coverage 80 X were obtained from LW-Hubei genome sequencing data. (DOCX 48 kb) [file 43008_2019_8_MOESM6_ESM.docx]

Additional file 6: **Figure S6** Statistics analysis for high quality subreads with estimated genome coverage 80 X were obtained from LW-Hubei genome sequencing data.


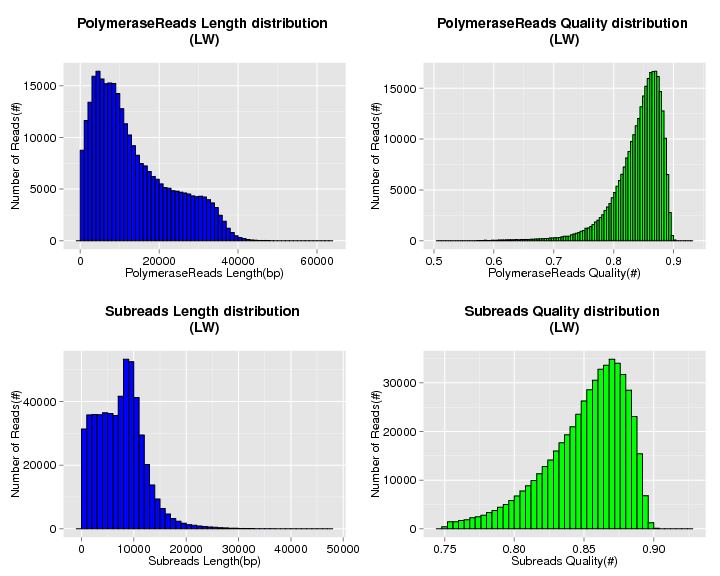


**Subreads Quality distribution**

**Subreads Length distribution**
