# Supplementary material for: Comprehensive analysis of full genome sequence and Bd-milRNA/target mRNAs to discover the mechanism of hypovirulence in Botryosphaeria dothidea strains on pear infection with BdCV1 and BdPV1
Source: IMA Fungus. 2019 Jun 7;10:3. doi: 10.1186/s43008-019-0008-4 (PMC7325678; doi:10.1186/s43008-019-0008-4)
Supplement: Supplementary file 7 — Figure S7. Whole genome synteny analysis of LW-Hubei strain by comparison with Macrophomina phascolina MS6, Neofusicoccum parvum UCRNP2, Diplodia corticola CBS 112549 and Dothistroma septosporum NZE10 at the nucleotide (nt) level. (a) The 49 scaffolds of LW-Hubei and Macrophomina phascolina MS6 were compared (Mb scale). (b) The 49 scaffolds of LW-Hubei and Neofusicoccum parvum UCRNP2 were compared (Mb scale). (c) The 49 scaffolds of LW-Hubei and Diplodia corticola CBS 112549 were compared (Mb scale). (d) The 49 scaffolds of LW-Hubei and Diplodia seriata were compared (Mb scale). (DOCX 3136 kb) [file 43008_2019_8_MOESM7_ESM.docx]

Additional file 7: **Figure S7** Whole genome synteny analysis of LW-Hubei strain by comparison with *Macrophomina phascolina* MS6, *Neofusicoccum parvum* UCRNP2, *Diplodia corticola* CBS 112549 and *Dothistroma septosporum* NZE10 at the nucleotide (nt) level. (a) The 49 scaffolds of LW-Hubei and *Macrophomina phascolina* MS6 were compared (Mb scale). (b) The 49 scaffolds of LW-Hubei and *Neofusicoccum parvum* UCRNP2 were compared (Mb scale). (c) The 49 scaffolds of LW-Hubei and *Diplodia corticola* CBS 112549 were compared (Mb scale). (d) The 49 scaffolds of LW-Hubei and *Diplodia seriata* were compared (Mb scale).


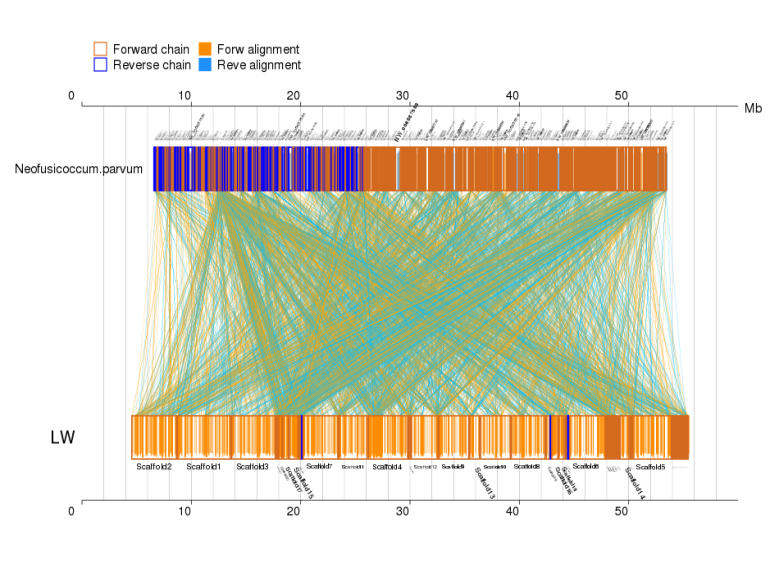

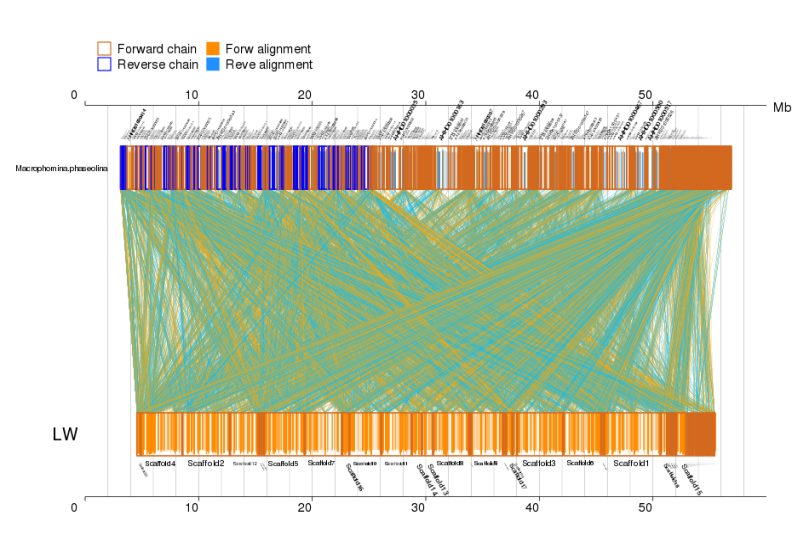


a

b


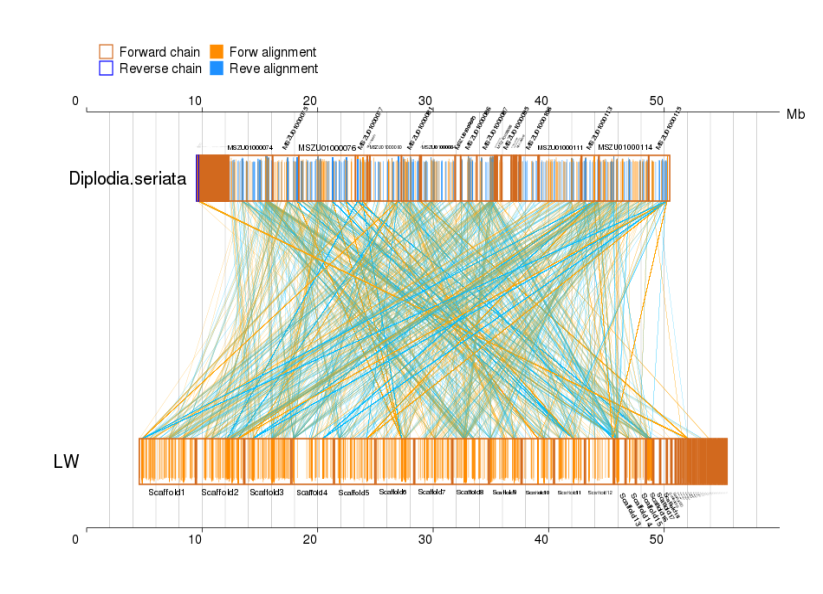

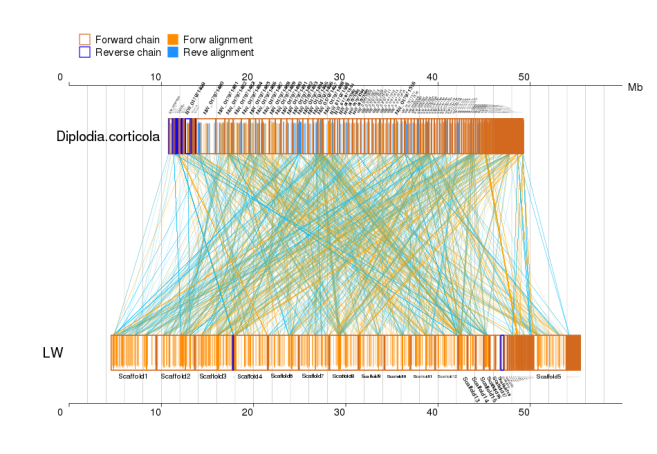


c

d
