# Supplementary material for: Comprehensive analysis of full genome sequence and Bd-milRNA/target mRNAs to discover the mechanism of hypovirulence in Botryosphaeria dothidea strains on pear infection with BdCV1 and BdPV1
Source: IMA Fungus. 2019 Jun 7;10:3. doi: 10.1186/s43008-019-0008-4 (PMC7325678; doi:10.1186/s43008-019-0008-4)

Additional file 8: **Figure S8** Venn diagram of the common and unique Core-pan genes among five *Botryosphaeriaceae* strains: *Botryosphaeria dothidea* LW-Hubei, *M. phaseolina* MS6, *N. parvum* UCRNP2*, D. corticola* CBS 112549and *D. seriata*.


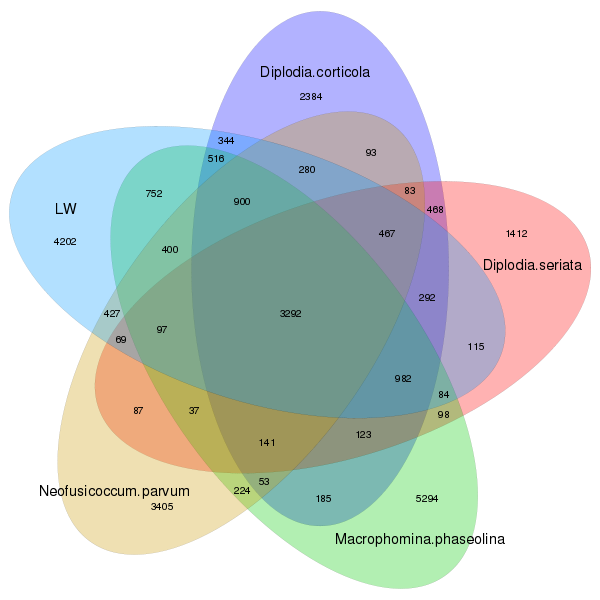

Supplement: Supplementary file 8 — Figure S8. Venn diagram of the common and unique Core-pan genes among five Botryosphaeriaceae strains: Botryosphaeria dothidea LW-Hubei, M. phaseolina MS6, N. parvum UCRNP2, D. corticola CBS 112549 and D. seriata. (DOCX 114 kb) [file 43008_2019_8_MOESM8_ESM.docx]
