# Supplementary material for: Comprehensive analysis of full genome sequence and Bd-milRNA/target mRNAs to discover the mechanism of hypovirulence in Botryosphaeria dothidea strains on pear infection with BdCV1 and BdPV1
Source: IMA Fungus. 2019 Jun 7;10:3. doi: 10.1186/s43008-019-0008-4 (PMC7325678; doi:10.1186/s43008-019-0008-4)
Supplement: Supplementary file 9 — Figure S9. Heatmap of the dispensable gene in five Botryosphaeriaceae strains. (DOCX 69 kb) [file 43008_2019_8_MOESM9_ESM.docx]

Additional file 9: **Figure S9** Heatmap of the dispensable gene in five *Botryosphaeriaceae* strains*.*


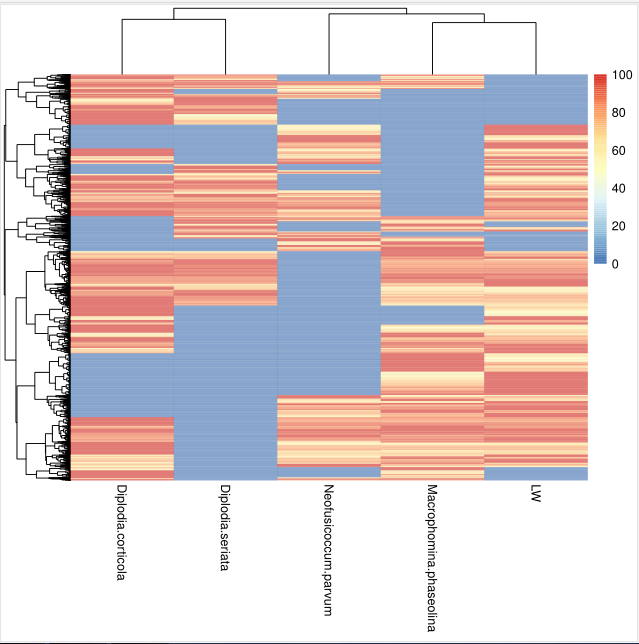


*Botryosphaeria dothidea* LW-Hubei

*Macrophomina*

*phaseolina*

*Neofusicoccum*

*parvum*

*Diplodia*

*seriata*

*Diplodia*

*corticola*
