# Supplementary material for: Comprehensive analysis of full genome sequence and Bd-milRNA/target mRNAs to discover the mechanism of hypovirulence in Botryosphaeria dothidea strains on pear infection with BdCV1 and BdPV1
Source: IMA Fungus. 2019 Jun 7;10:3. doi: 10.1186/s43008-019-0008-4 (PMC7325678; doi:10.1186/s43008-019-0008-4)

Additional file 10: **Figure S10** Phylogenetic tree analysis based on gene family from *Botryosphaeria dothidea* LW-Hubei and the eight reference fungi by neighbor-joining method. The scale number represents branch lengths.


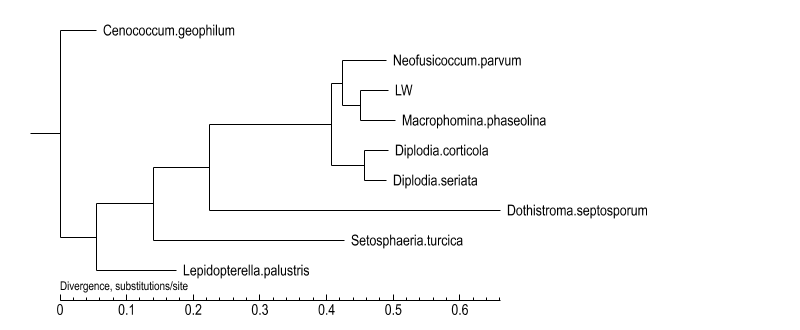


***Botryosphaeriaceae***

Divergence, substitutions/site


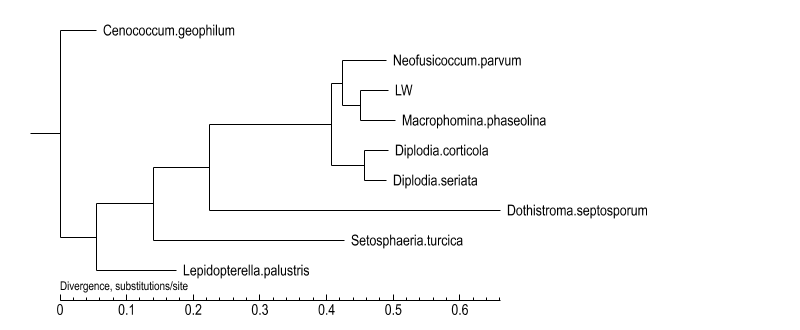

Supplement: Supplementary file 10 — Figure S10. Phylogenetic tree analysis based on gene families from Botryosphaeria dothidea LW-Hubei and the eight reference fungi by the neighbor-joining method. The scale number represents branch lengths. (DOCX 65 kb) [file 43008_2019_8_MOESM10_ESM.docx]
