# Supplementary material for: Comprehensive analysis of full genome sequence and Bd-milRNA/target mRNAs to discover the mechanism of hypovirulence in Botryosphaeria dothidea strains on pear infection with BdCV1 and BdPV1
Source: IMA Fungus. 2019 Jun 7;10:3. doi: 10.1186/s43008-019-0008-4 (PMC7325678; doi:10.1186/s43008-019-0008-4)

Additional file 11: **Figure S11** Annotation of the LW-Hubei genome by Nr and KOG databases. (a) Annotation of 12,273 proteins by the Nr database. Large percentage of genes in LW-Hubei genome with homologs in *Macrophomina phascolina*. (b) Annotation of 2,536 proteins by the KOG database for delineation of 25 Clusters of Orthologous Groups of proteins (COG). The majority of proteins (n = 292) were classified into General function prediction only.


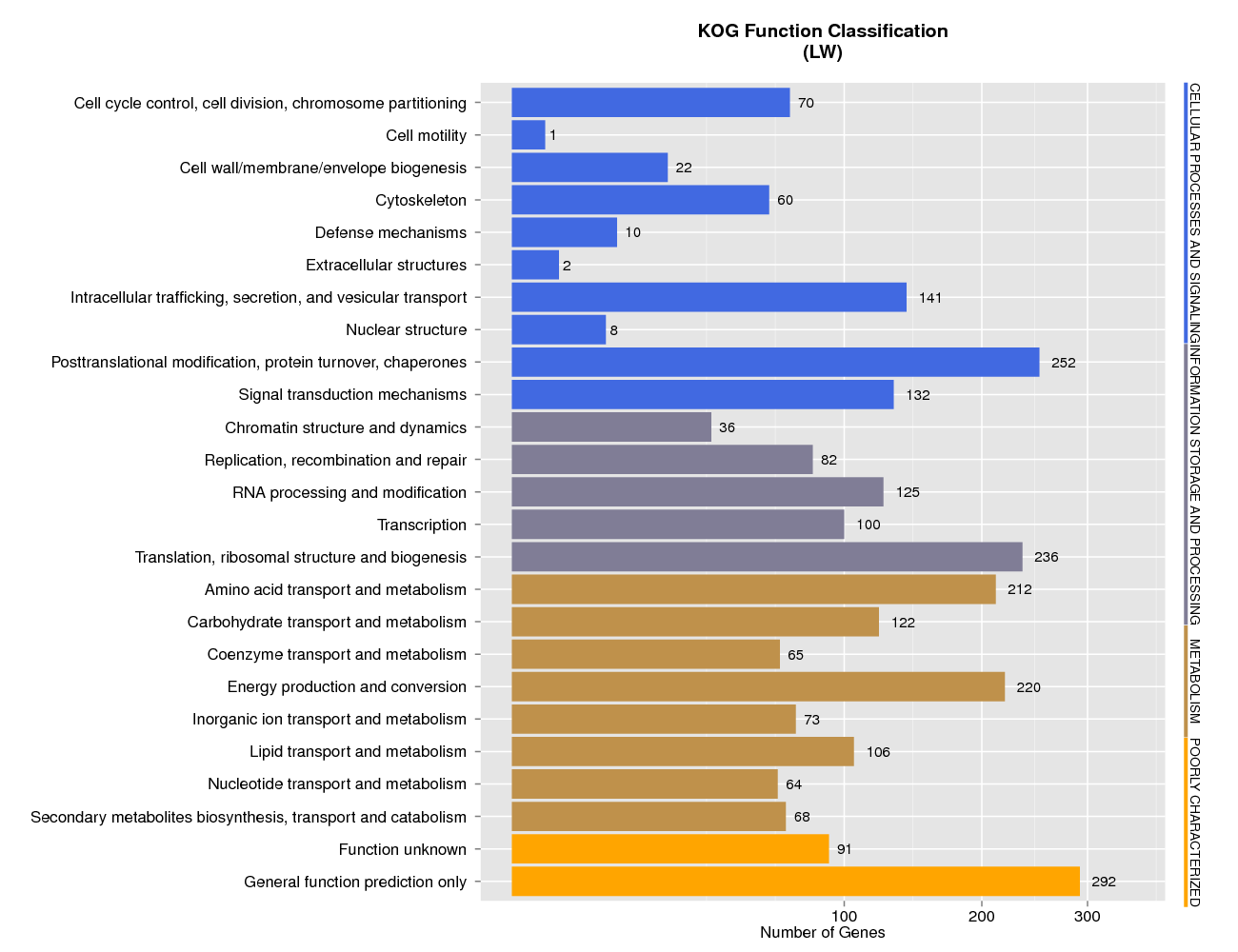


b

a


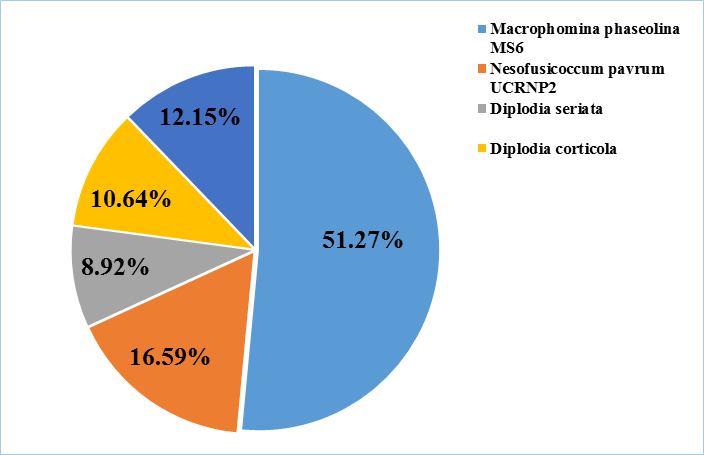


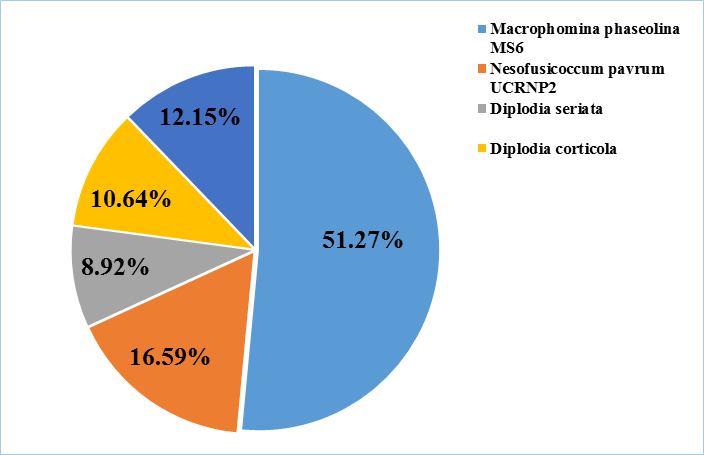

Supplement: Supplementary file 11 — Figure S11. Annotation of the LW-Hubei genome by Nr and KOG databases. (a) Annotation of 12,273 proteins by the Nr database. Large percentage of genes in LW-Hubei genome with homologs in Macrophomina phascolina. (b) Annotation of 2536 proteins by the KOG database for delineation of 25 Clusters of Orthologous Groups of proteins (COG). The majority of proteins (n = 292) were classified into General function prediction only. (DOCX 141 kb) [file 43008_2019_8_MOESM11_ESM.docx]
