# Supplementary material for: Comprehensive analysis of full genome sequence and Bd-milRNA/target mRNAs to discover the mechanism of hypovirulence in Botryosphaeria dothidea strains on pear infection with BdCV1 and BdPV1
Source: IMA Fungus. 2019 Jun 7;10:3. doi: 10.1186/s43008-019-0008-4 (PMC7325678; doi:10.1186/s43008-019-0008-4)

Additional file12: Figure S12 The precursors of five novel milRNAs and their hairpin structures in *Botryosphaeria dothidea* strains. The mature *Bd*-milRNAs are shown in yellow and milRNA*s are underlined in green. The numbers show the base locations.

***Bd*-milR55**

AGCUUCGCGCUAUCUCGCCCGGCUUCUUAUUGGCGGAAGCAACAGCCGG
GCAUGGCAGUAUAGAACAUGCACGCUUUGCUUAAUACCAAGCUUAAGG
GUCGGUAGCAUGCUCUCGUUAGGAGAAUCCGGGCGCGACCGGCUA

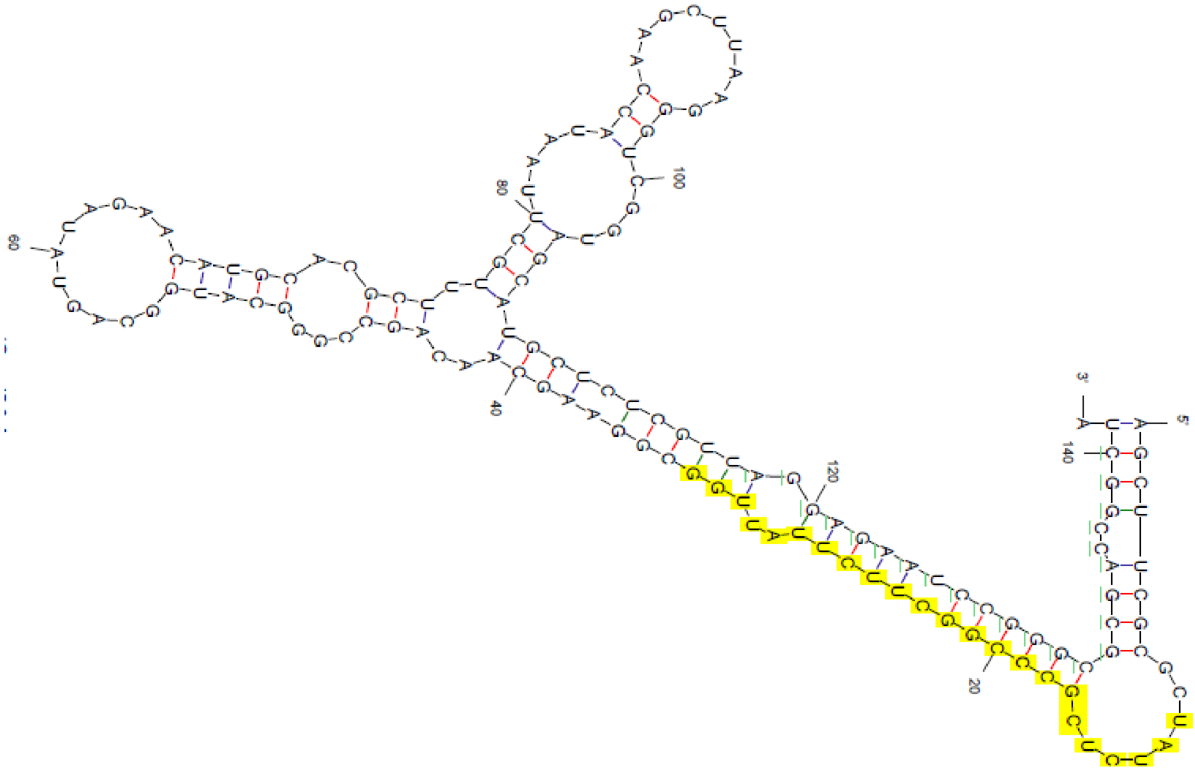

***Bd*-milR59**

GGUAGUUGCAAUUGUGUUUGCAGAGCUGGAGCAUUCGUUGAAUGUUUA
GAAGGGCUGUUCACAAUUUCAUCUAGUUGAAUGCUCCAGCUCUGCAAG
UACAACUGCACUCGCUAUUGCAAUACUUUUGCAACCGACCU

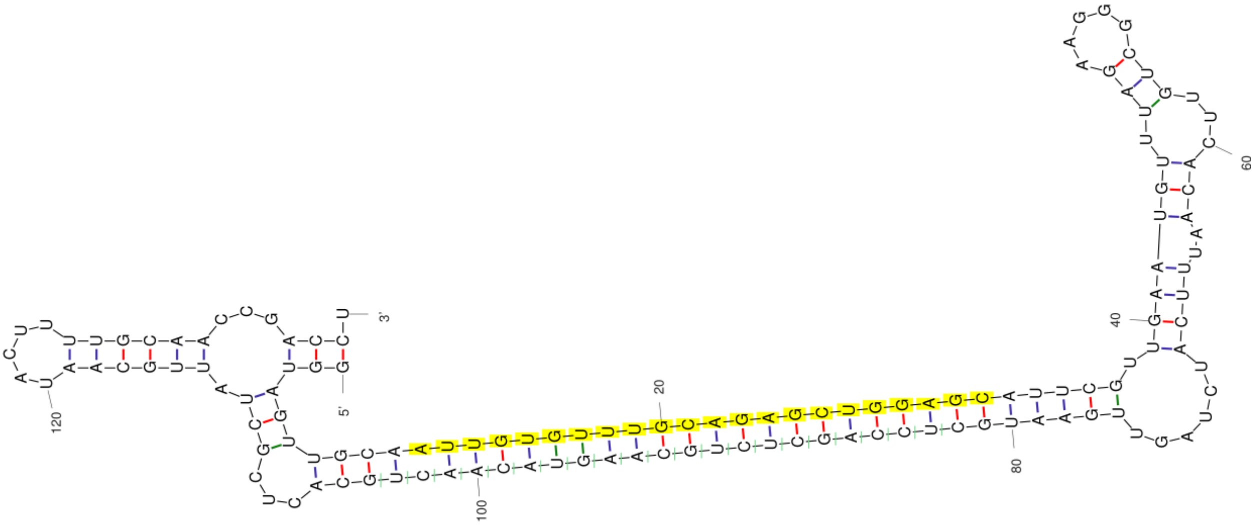

***Bd*-milR65**

UCCGCGGAAAAGCAUCACAGUGGGUCUCAAAAGUUCUUGACCUUCGAG
CCUACUUAGUUCACUGAGAUUUGGUAGGGCGGCUAAAUUCCGGAGGUC
AAGAACUUUUGAGACCCACUGUGGCAUGAAUAUCCAUUA

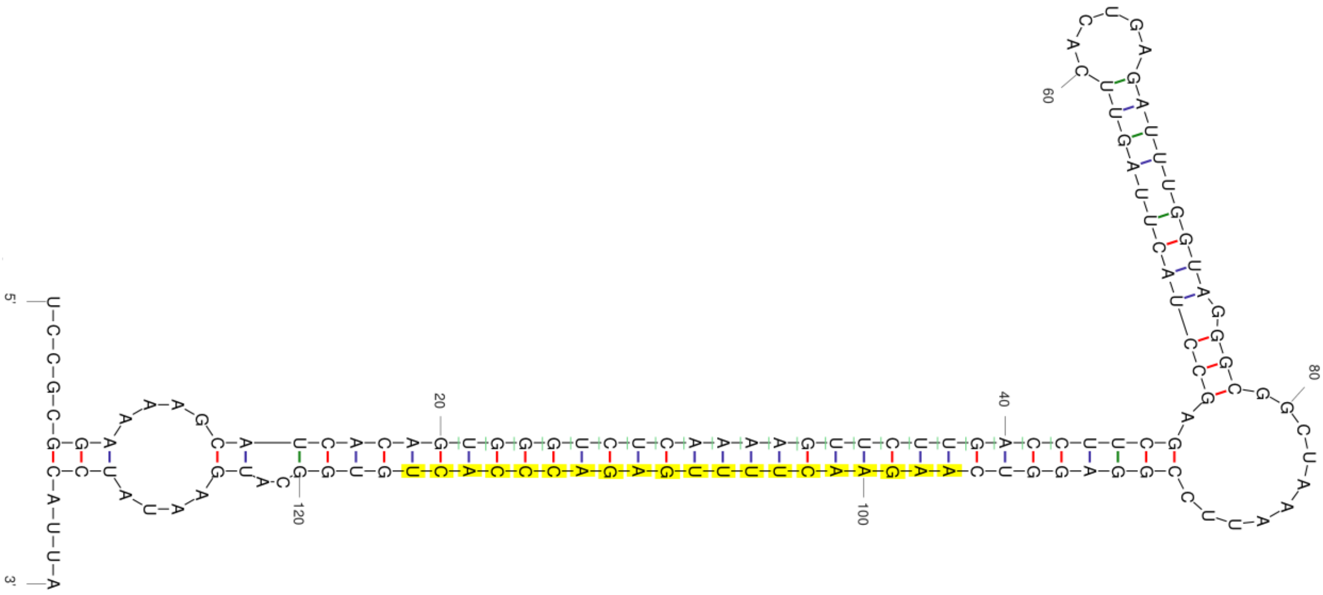

***Bd*-milR49**

UCUUCACGACUGCGGUCUAGAGAAAAUCCUGUACAACACGGCUUAGCC
AGGGGUCUUCUGCAGCUCUAAUUCUCUUCUCACGGAAACCACAUUUAU
GCUUUGCUUUUUAUCCUCUCGACGAACAGGAGGUUUUUCUCGGGUUGU
AGCUAGUCAUUUUCGAUUCCCCUUUGCAGAAGAG

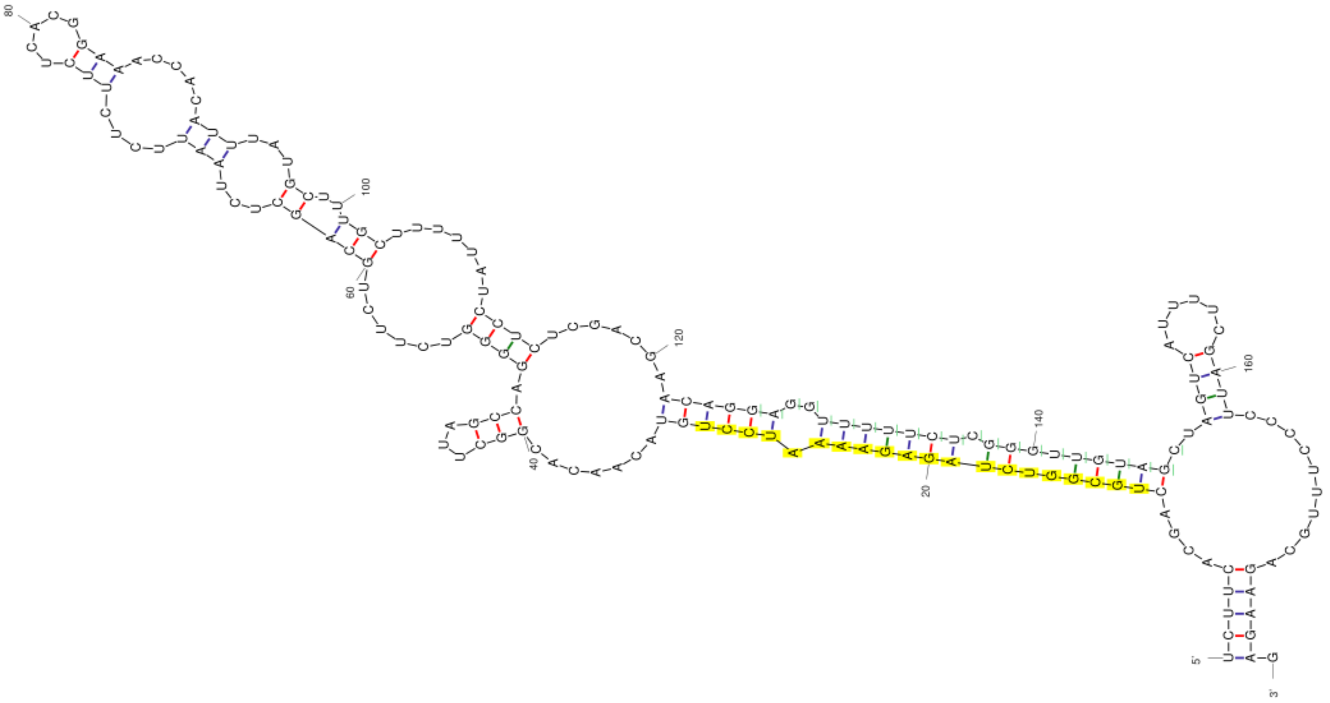

***Bd*-milR20**

UGCAGGCUCAUGGGCAGUCUGUGCGUGAGCUCCGGAGGUCGACCUUCC
GGGAUGAUUGGUUCAUGACGCAGAUGCCUAC

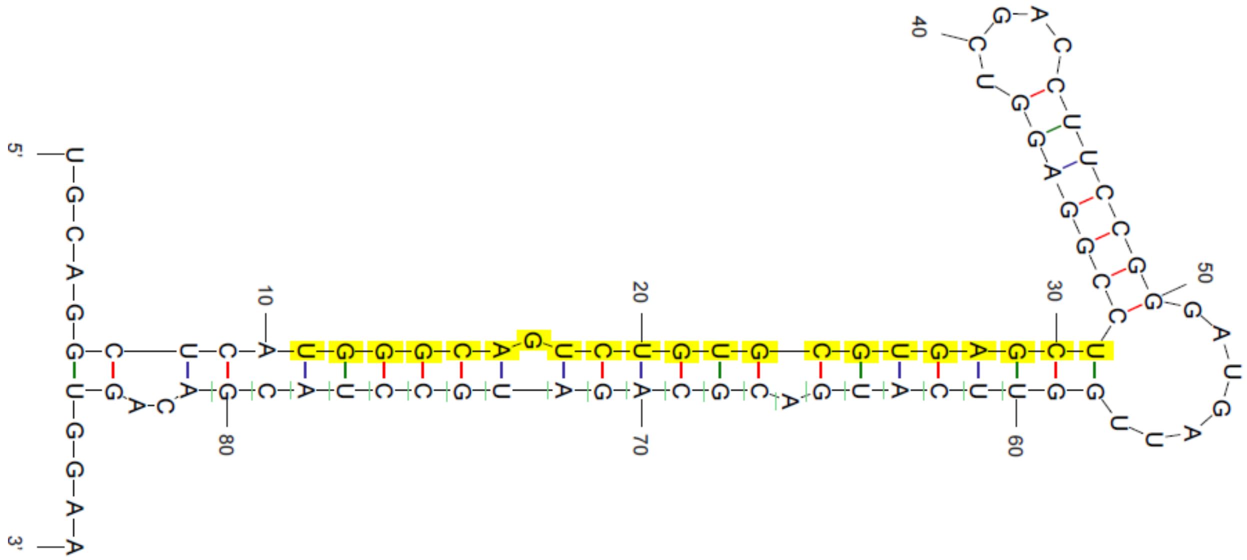

Supplement: Supplementary file 12 — Figure S12. The precursors of five novel milRNAs and their hairpin structures in Botryosphaeria dothidea strains. The mature Bd-milRNAs are shown in yellow and milRNA*s are underlined in green. The numbers show the base locations. (PDF 203 kb) [file 43008_2019_8_MOESM12_ESM.pdf]
