# Supplementary material for: Comprehensive analysis of full genome sequence and Bd-milRNA/target mRNAs to discover the mechanism of hypovirulence in Botryosphaeria dothidea strains on pear infection with BdCV1 and BdPV1
Source: IMA Fungus. 2019 Jun 7;10:3. doi: 10.1186/s43008-019-0008-4 (PMC7325678; doi:10.1186/s43008-019-0008-4)
Supplement: Supplementary file 13 — Figure S13. First nucleotide bias in novel Bd-milRNA candidates isolated from Botryosphaeria dothidea strains in (a) Mock, (b) LW-C, (c) LW-P and (d) LW-CP libraries. (DOCX 215 kb) [file 43008_2019_8_MOESM13_ESM.docx]

Additional file 13: **Figure S13** First nucleotide bias in novel *Bd*-milRNA candidates isolated from *Botryosphaeria dothidea* strains in (a) Mock, (b) LW-C, (c) LW-P and (d) LW-CP libraries.

a


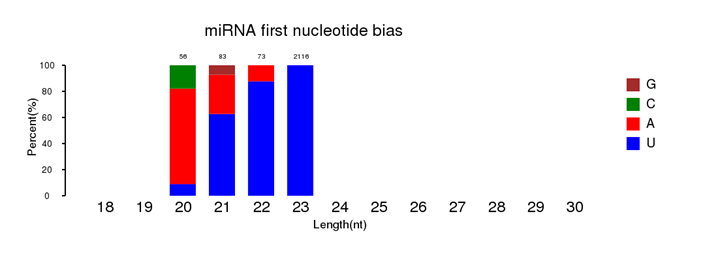

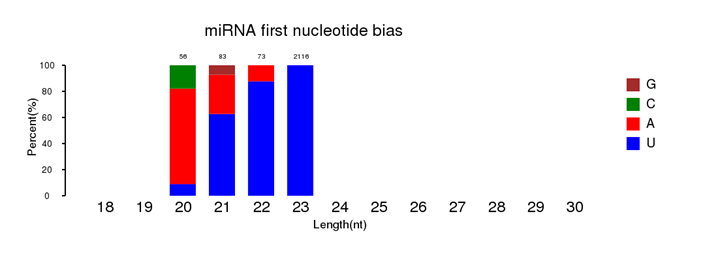

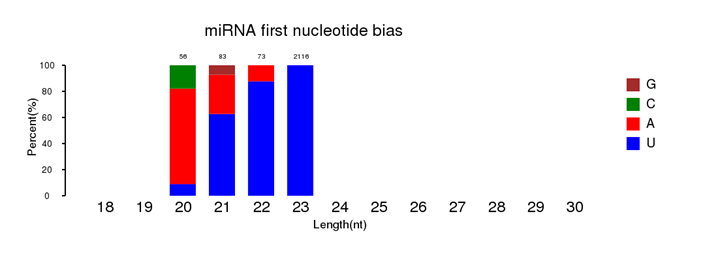


b


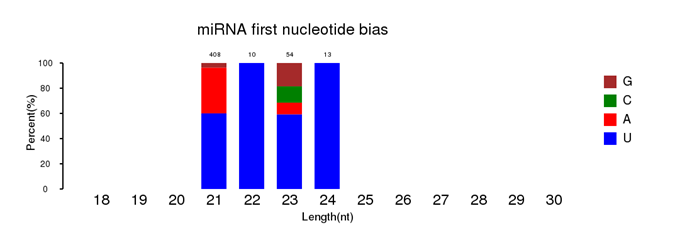

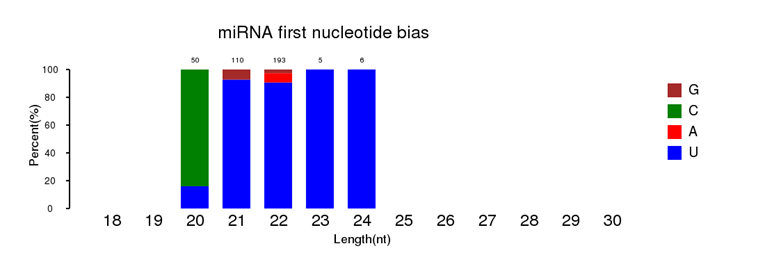

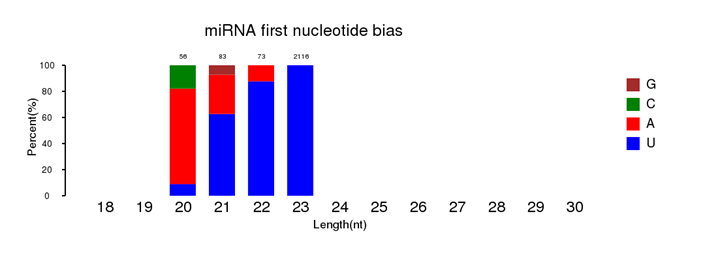


d

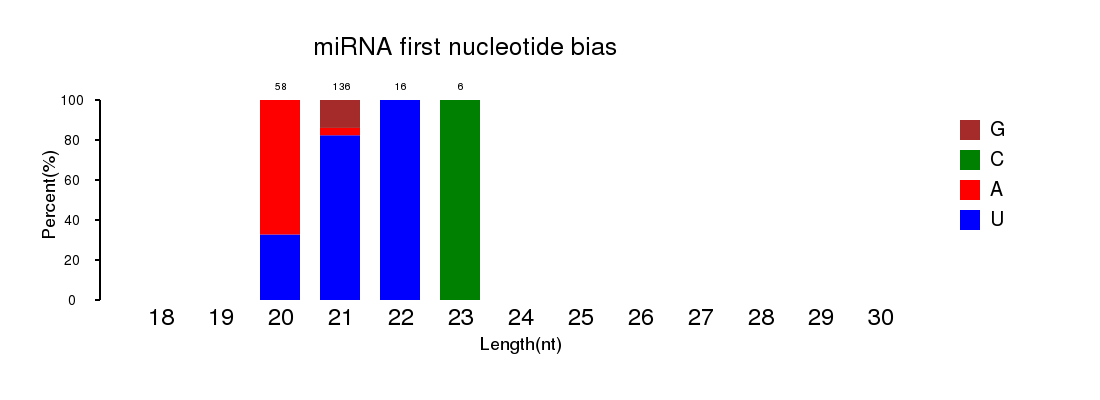

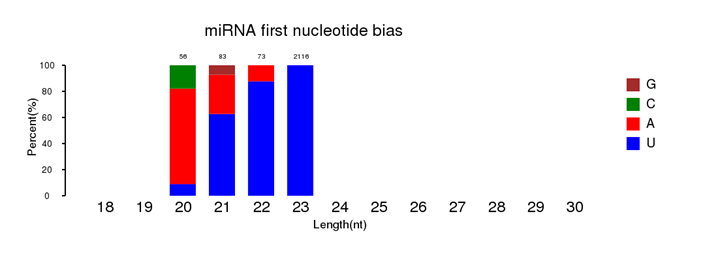


c
