# Supplementary material for: Comprehensive analysis of full genome sequence and Bd-milRNA/target mRNAs to discover the mechanism of hypovirulence in Botryosphaeria dothidea strains on pear infection with BdCV1 and BdPV1
Source: IMA Fungus. 2019 Jun 7;10:3. doi: 10.1186/s43008-019-0008-4 (PMC7325678; doi:10.1186/s43008-019-0008-4)
Supplement: Supplementary file 14 — Figure S14. Putative Bd-milRNAs (two novel and one known) detected by poly (A) RT-PCR. (DOCX 233 kb) [file 43008_2019_8_MOESM14_ESM.docx]

Additional file 14: **Figure S14** Putative *Bd*-milRNAs (two novel and one known) detected by poly (A) RT-PCR.


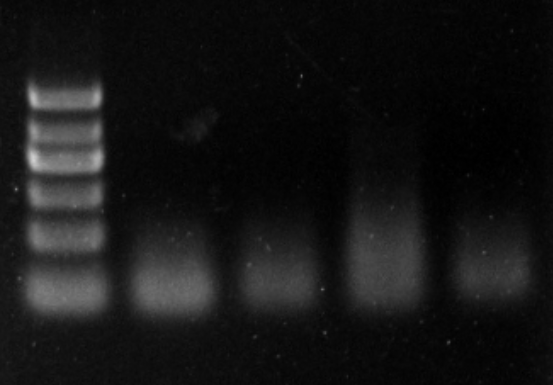


M *Bd*-milR6 *Bd*-milR12 *Bd*-milR1147.2 U6
