# Supplementary figures and images for: Comprehensive analysis of full genome sequence and Bd-milRNA/target mRNAs to discover the mechanism of hypovirulence in Botryosphaeria dothidea strains on pear infection with BdCV1 and BdPV1
Source: IMA Fungus. 2019 Jun 7;10:3. doi: 10.1186/s43008-019-0008-4 (PMC7325678; doi:10.1186/s43008-019-0008-4)

**Additional file 15: Figure S15** Expression levels of *Bd*-miRNA target mRNAs detected by RT-qPCR.

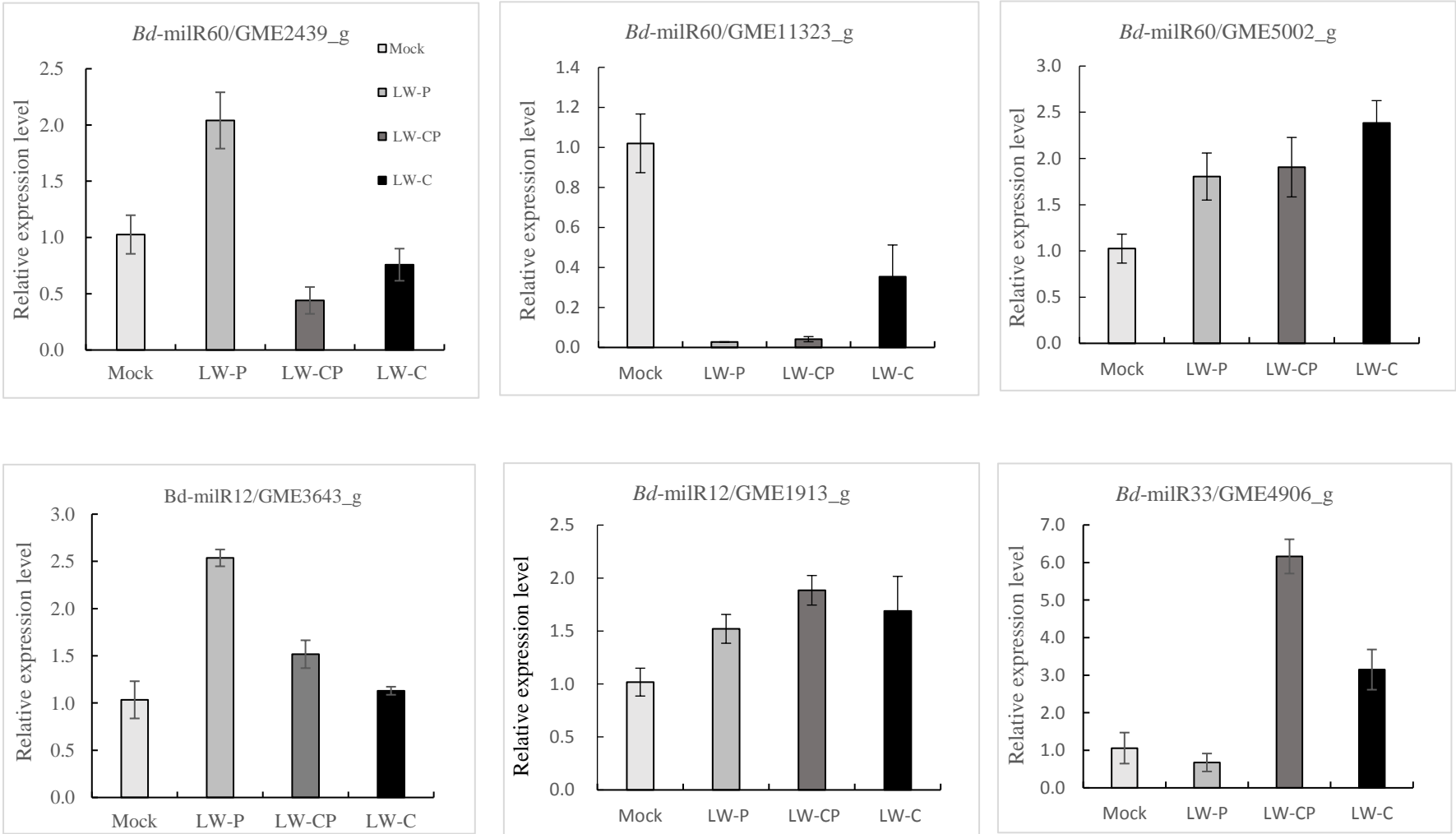

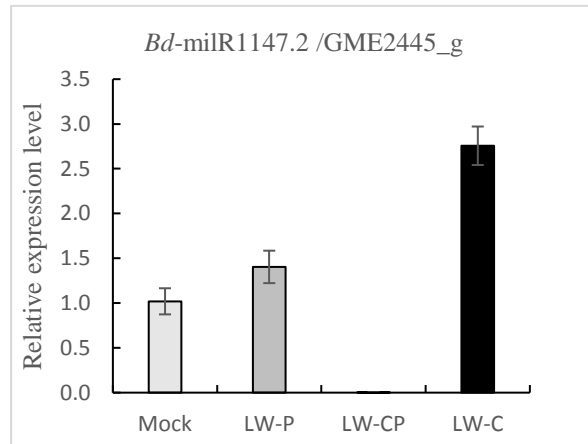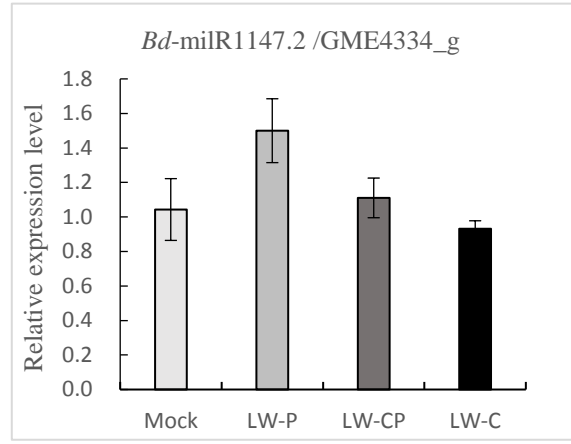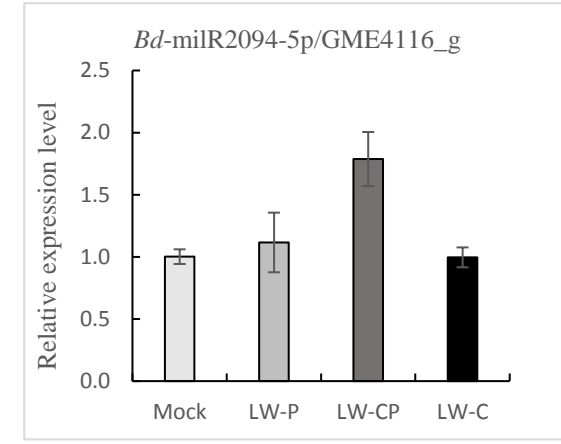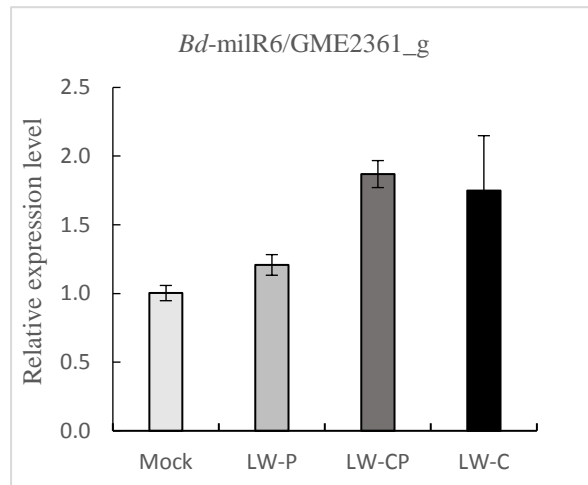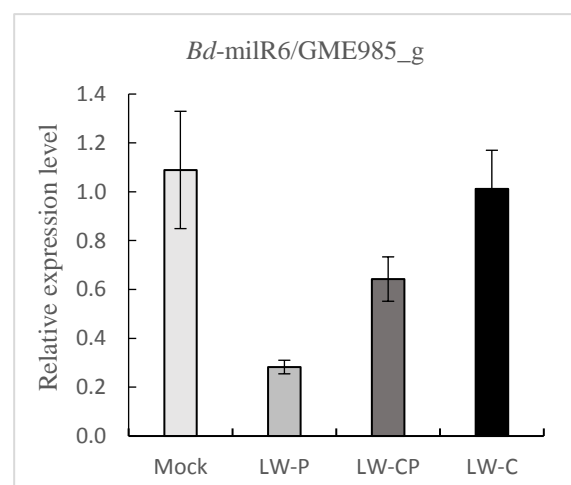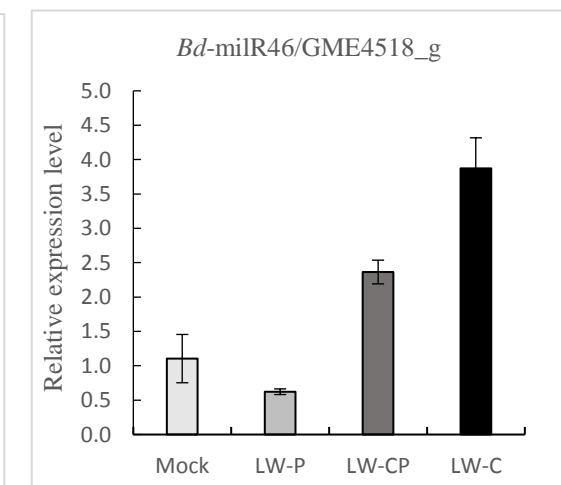

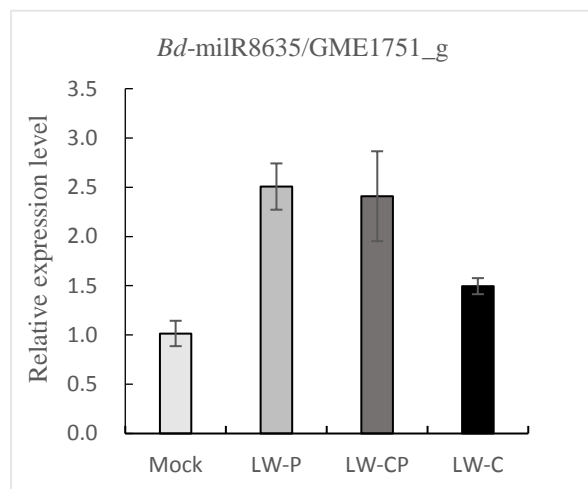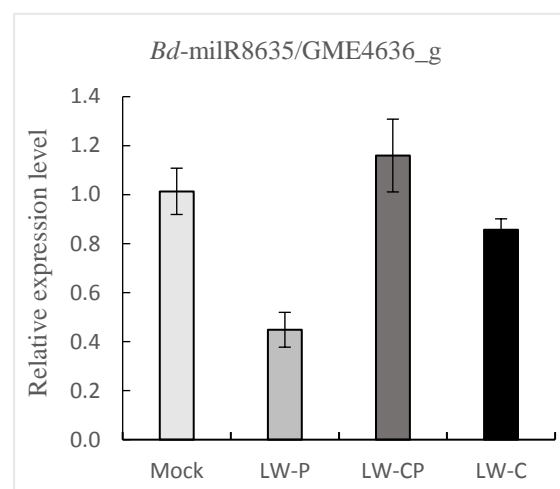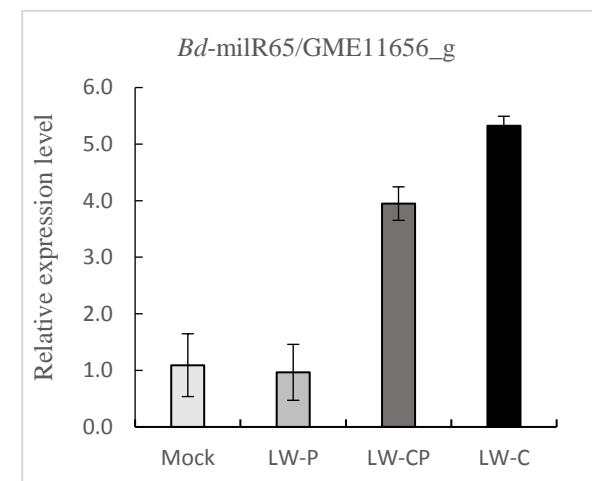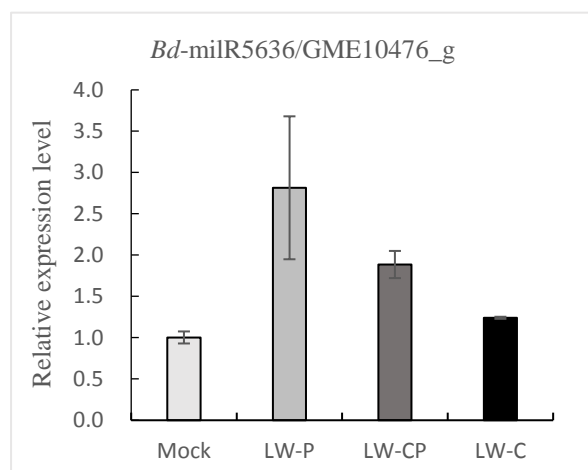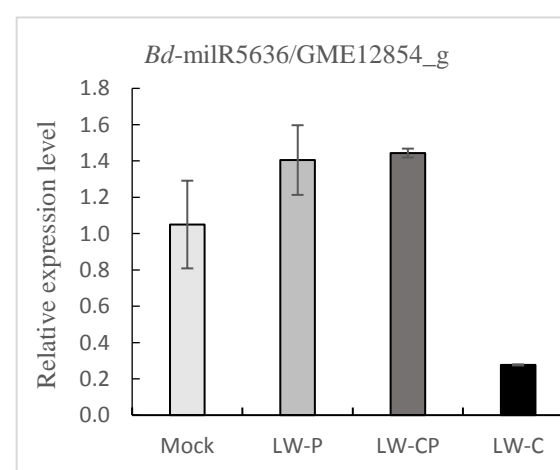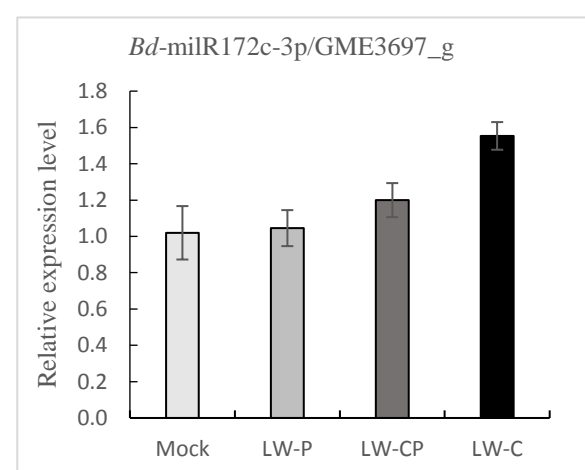

Supplement: Supplementary file 15 — Figure S15. Expression levels of Bd-milRNA target mRNAs detected by RT-qPCR. (PDF 282 kb) [file 43008_2019_8_MOESM15_ESM.pdf]
