# Supplementary material for: Comprehensive analysis of full genome sequence and Bd-milRNA/target mRNAs to discover the mechanism of hypovirulence in Botryosphaeria dothidea strains on pear infection with BdCV1 and BdPV1
Source: IMA Fungus. 2019 Jun 7;10:3. doi: 10.1186/s43008-019-0008-4 (PMC7325678; doi:10.1186/s43008-019-0008-4)
Supplement: Supplementary file 16 — Figure S16. KEGG pathway classifications of functional enrichment for differentially expressed known (a) and novel (b) Bd-milRNA target mRNAs in mycovirus-infected Botryosphaeria dothidea strains of LW-CP, LW-C and LW-P. (PDF 125 kb) [file 43008_2019_8_MOESM16_ESM.pdf]

Additional file 16: Figure S16 KEGG pathway classifications of functional enrichment for differentially expressed known (a) and novel (b) *Bd*-milRNA target mRNAs in mycovirus-infected *Botryosphaeria dothidea* strains of LW-1(LW-CP), LW-C and LW-P.

a

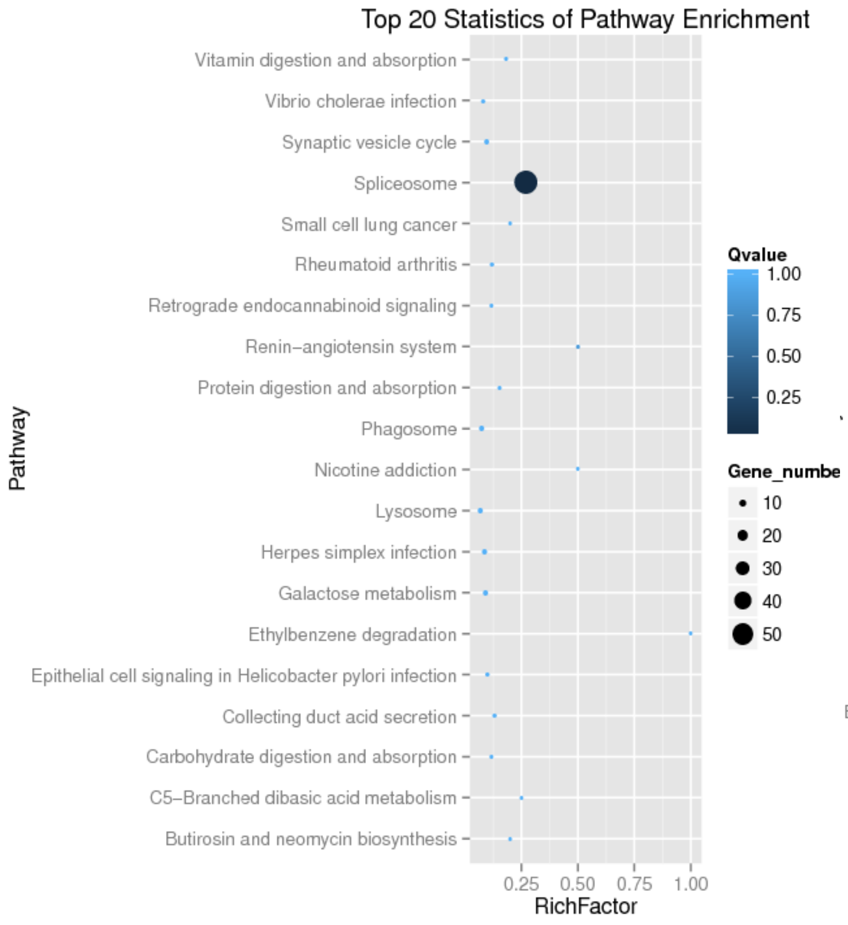

LW-CP/Mock

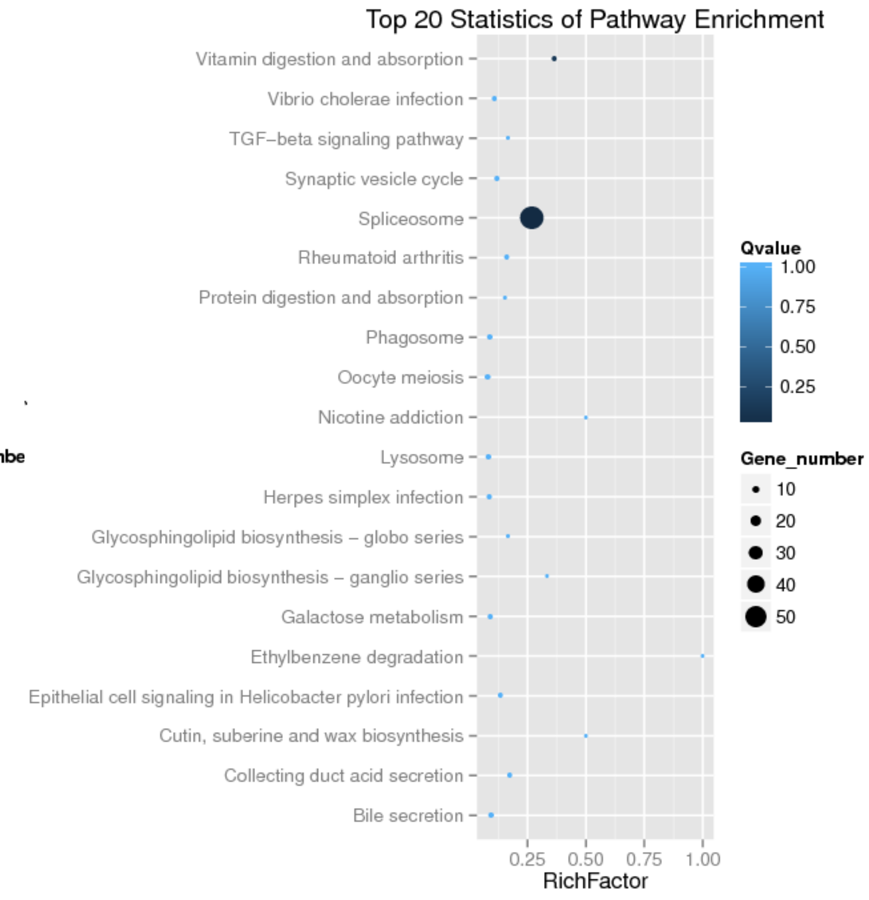

LW-CP/Mock

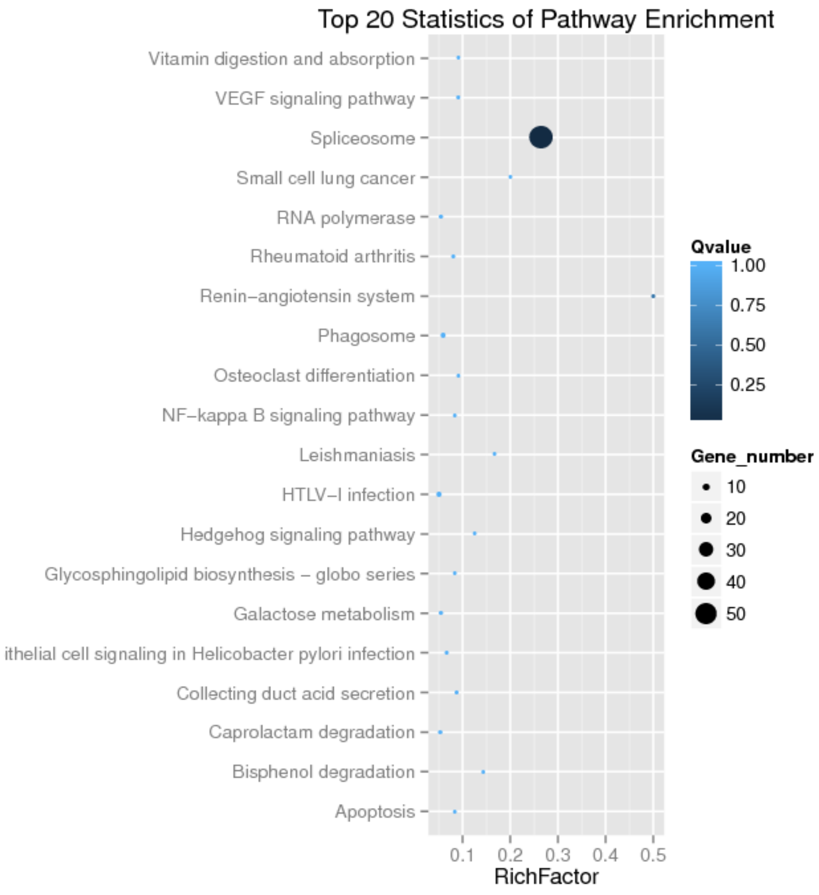

LW-P/Mock

b

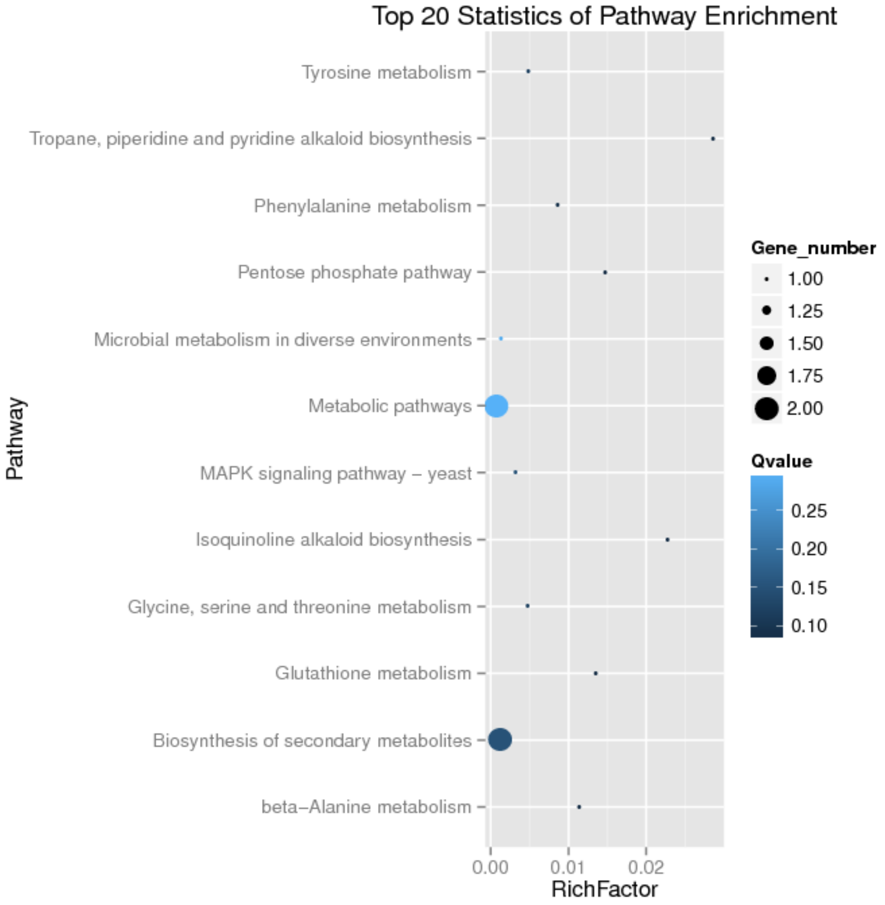

LW-CP/Mock

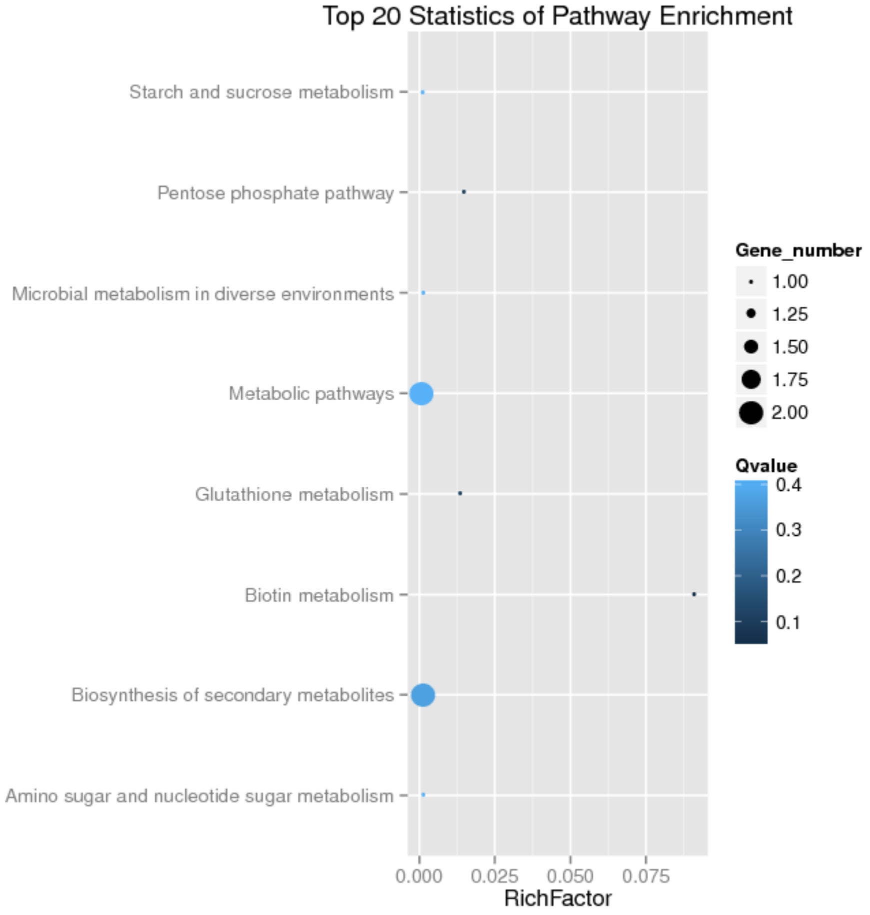

LW-C/Mock
