# Supplementary material for: Comprehensive analysis of full genome sequence and Bd-milRNA/target mRNAs to discover the mechanism of hypovirulence in Botryosphaeria dothidea strains on pear infection with BdCV1 and BdPV1
Source: IMA Fungus. 2019 Jun 7;10:3. doi: 10.1186/s43008-019-0008-4 (PMC7325678; doi:10.1186/s43008-019-0008-4)
Supplement: Supplementary file 17 — Table S1. Sequences of qPCR primers used for analysis of the expression level of RNAi components in Botryosphaeria dothidea strains. (DOCX 14 kb) [file 43008_2019_8_MOESM17_ESM.docx]

Additional file 17: **Table S1** Sequences of qPCR primers used for analysis of the expression level of RNAi components in *Botryosphaeria dothidea* strains.

|  | **Gene name** | **Primer sequence (5′-3′)** | **Size of primers (bp)** |
| --- | --- | --- | --- |
| RNAi components | GME11353_g -F | CACAACAAAGCCCGTCTGGAG | 21 |
|  | GME11353_g -R | GAATCAGAACCTTGCCCGACT | 21 |
|  | GME9732_g -F | CCGCCCTTACCATGTCGTT | 19 |
|  | GME9732_g -R | CCAATGAGTGACCATCCGACCA | 22 |
|  | GME11473_g -F | ATAACTTCCTCCGTGTCCAGT | 21 |
|  | GME11473_g -R | GAAATGCGAAACTATGCCCTCC | 22 |
|  | GME8162_g -F | CAGTGTCCGATTTCCTCTTGC | 21 |
|  | GME8162_g -R | GCTGCTCCTCGTAAATTTCCCT | 22 |
|  | GME9357_g -F | CCAGGCAACCAAGCTATACGAA | 22 |
|  | GME9357_g -R | TTCTTTGCGTAATCCACCAG | 20 |
|  | GME12310_g -F | TGACGACCACTCTATGATGACG | 22 |
|  | GME12310_g -R | GAGGAGTTTAGCAGAGCGGAAT | 22 |
|  | GME553_g -F | GGCTGAGATGGTCGAGGAG | 19 |
|  | GME553_g -R | CTTGCTGATGGCAGAGGGT | 19 |
|  | GME6306_g -F | AGCCCGTATTCTGCCTGTC | 19 |
|  | GME6306_g -R | CAAATCCAAAGCCTGTTCC | 19 |
| Actin | GME8592_g -F | CCGCTCCGTTTCTATGCTCT | 20 |
|  | GME8592_g -R | ACCCTCACCGACATACCAGT | 20 |
