# Supplementary material for: Comprehensive analysis of full genome sequence and Bd-milRNA/target mRNAs to discover the mechanism of hypovirulence in Botryosphaeria dothidea strains on pear infection with BdCV1 and BdPV1
Source: IMA Fungus. 2019 Jun 7;10:3. doi: 10.1186/s43008-019-0008-4 (PMC7325678; doi:10.1186/s43008-019-0008-4)
Supplement: Supplementary file 18 — Table S2. The sequences of primers used for analysis of the expression of putative Bd-milRNAs and target gene mRNAs in Botryosphaeria dothidea strains. (DOCX 16 kb) [file 43008_2019_8_MOESM18_ESM.docx]

Additional file 18: **Table S2** The sequences of primers used for analysis of the expression of putative *Bd*-milRNAs and target gene mRNAs in *Botryosphaeria dothidea* strains.

| **RT-(q)PCR** | **Gene name** | **Primer sequence (5′-3′)** | **Size of primers (bp)** |
| --- | --- | --- | --- |
| Stem-loop RT-PCR for milRNA | *Bd*-milR172-F | GCGAGAATCTTGATTGT | 17 |
|  | *Bd*-milR172-RT | GTCGTATCCAGTGCAGGGTCCGAGGTATTCGCACTGGATACGACGAGC | 48 |
|  | *Bd*-milR8635-F | GGGTAGGTTCAAACG | 15 |
|  | *Bd*-milR8635-RT | GTCGTATCCAGTGCAGGGTCCGAGGTATTCGCACTGGATACGACCTCC | 48 |
|  | *Bd*-milR5636-F | CCCCGTAGTCGCGAGCTT | 18 |
|  | *Bd*-milR5636-RT | GTCGTATCCAGTGCAGGGTCCGAGGTATTCGCACTGGATACGACCCGA | 48 |
|  | Reverse primer | GTGCAGGGTCCGAGGT | 16 |
| poly(A) RT-PCR for milRNA | Bd-milR6 | TTTCGGGACTTCGTACTGACC | 21 |
|  | Bd-milR12 | ACGCTCCCGAAGGAACTGGCT | 21 |
|  | Bd-milR1147.2 | GCTCGGACAGTTGGCAGA | 18 |
|  | RT/ Reverse primer | The kit provides |  |
| RT-qPCR for Target gene | GME3643_g-F | ACCATTCTTTCTGCCATCGAG | 21 |
|  | GME364_3-g-R | TACGAGCACAAGACTCAAAGCA | 22 |
|  | GME1913_g-F | ATCTTCAACGCCAACCTCGAC | 21 |
|  | GME1913_g-R | AGAGCTGAAGTACCACGACCA | 21 |
|  | GME4906_g-F | CAAAGCACCCATCACGGTCA | 20 |
|  | GME4906_g-R | CCTCGATCTGGTCTTGAAGCA | 21 |
|  | GME2439_g-F  GME2439 | GCCGCCATTCCCTCCCAA | 18 |
|  | GME2439_g-R | TTTCTTCGCCCCATCCTCGT | 20 |
|  | GME11323_g-F | ACGCAAACTCCATTTTCCTCC | 21 |
|  | GME11323_g-R | TAACATCACGTAGGCTTCGAT | 21 |
|  | GME5002_g-F | CGTTGATCTCGGTTCTGCTCT | 21 |
|  | GME5002_g-R | CTTGACGCTGAAACCCTCTCG | 21 |
|  | GME2445_g-F | TCACTCAGCATTCGTCCCA | 19 |
|  | GME2445_g-R | TCTGCGGTTCAACATCGAC | 19 |
|  | GME4334_g-F | TGTCAACACTCATAATCGACCC | 22 |
|  | GME4334_g-R | CGAAACCATTGAGCCGACCAC | 21 |
|  | GME4116-g-F | GTCACTACGGCTCTCACCAC | 20 |
|  | GME4116_g-R | GCGTATTGGTCGTCCTCCTC | 20 |
|  | GME4518_g-F | CCAAGTTCTGCACCTACGTT | 20 |
|  | GME4518_g-R | CTGACTCCTGCCCTTCTCG | 19 |
|  | GME2361_g-F | AGACTTGAACACCCACGACT | 20 |
|  | GME2361_g-R | ACGCTTACTGCCCTTTCCCT | 20 |
|  | GME985_g-F | CGACTTCACCTACGGGCAT | 19 |
|  | GME985_g-R | CATCCTTCCACGGGTCGAAC | 20 |
|  | GME11656_g-F | TTCATCGCTTGTGCTGTCC | 19 |
|  | GME11656_g-R | ATCATTGCCACCGTCGCCTT | 20 |
|  | GME 3697_g-F | CCGCCGTACCAATCAGCTT | 19 |
|  | GME 3697_g-R | GCCCATCCAGCTTCTGCAT | 19 |
|  | KF688737-F | CTGTCCTCACGGCTACACT | 19 |
|  | KF688737-R | CATGTGCGATCCTCCCCAG | 19 |
|  | GME10476_ g -F | GTTCTCGCTTTCCACGGCAA | 20 |
|  | GME10476-g-R | TCATACGGTCGAAGTAAACCAG | 22 |
|  | GME12854_g-F | CGCCGTTTTCCTCGCCATC | 19 |
|  | GME12854_g-R | TCCAATGGTGTAACGGCTCT | 20 |
|  | KF688736-F | TCGCAATCAGCATTCGACACC | 21 |
|  | KF688736-R | TCAGACATTTCCACGAGCACT | 21 |
|  | GME1751_g-F | CCGGGACTTACACCACGAAC | 20 |
|  | GME1751_g-R | GCCAATATCCGAGTCTCCCT | 20 |
|  | GME8592_g -F | CCGCTCCGTTTCTATGCTCT | 20 |
| Actin gene | GME8592_g -R | ACCCTCACCGACATACCAGT | 20 |
