# Supplementary material for: Comprehensive analysis of full genome sequence and Bd-milRNA/target mRNAs to discover the mechanism of hypovirulence in Botryosphaeria dothidea strains on pear infection with BdCV1 and BdPV1
Source: IMA Fungus. 2019 Jun 7;10:3. doi: 10.1186/s43008-019-0008-4 (PMC7325678; doi:10.1186/s43008-019-0008-4)
Supplement: Supplementary file 19 — Table S3. The sizes of conidia from Botryosphaeria dothidea strains. (DOCX 13 kb) [file 43008_2019_8_MOESM19_ESM.docx]

Additional file 19: **Table S3** The sizes of conidia from *Botryosphaeria dothidea* strains.

| **Isolates** | **Conidial size ranges (μm)** | **Mean ± SD (μm)** |
| --- | --- | --- |
| **LW-CP`** | 20.646~28.772×4.121~7.147 | 23.907±1.692×5.734±0.513 |
| **LW-C** | 22.504~31.395×4.293~6.736 | 27.595±1.626×5.592±0.456 |
| **LW-P** | 21.542~30.237×5.728~8.570 | 26.282±1.650×7.350±0.618 |
| **Mock** | 20.01~25.436×4.571~6.137 | 22.696±1.374×5.288±0.322 |
