# Supplementary material for: Comprehensive analysis of full genome sequence and Bd-milRNA/target mRNAs to discover the mechanism of hypovirulence in Botryosphaeria dothidea strains on pear infection with BdCV1 and BdPV1
Source: IMA Fungus. 2019 Jun 7;10:3. doi: 10.1186/s43008-019-0008-4 (PMC7325678; doi:10.1186/s43008-019-0008-4)
Supplement: Supplementary file 20 — Table S4. Summary of original sequencing data for Botryosphaeria dothidea LW-Hubei isolate generated using the Illumina platform. (DOCX 13 kb) [file 43008_2019_8_MOESM20_ESM.docx]

Additional file 20: **Table S4** Summary of original sequencing data for *Botryosphaeria dothidea* LW-Hubei isolate from Illumina generated using the Illumina platform.

| Sequencing technology | Insert size (bp) | Read length (bp) | Raw data (Mb) | Total Reads | Filtered Reads (%) | Low Quality Filtered Reads (%) | Trimmed data (Mb) |
| --- | --- | --- | --- | --- | --- | --- | --- |
| Illumina | 270 | 150:150 | 2,171 | 14,477,392 | 8.94 | 2.8 | 1,977 |
|  | 10,000 | 150:150 | 6,071 | 40,476,750 | 19.98 | 4.78 | 4,858 |
