# Supplementary material for: Comprehensive analysis of full genome sequence and Bd-milRNA/target mRNAs to discover the mechanism of hypovirulence in Botryosphaeria dothidea strains on pear infection with BdCV1 and BdPV1
Source: IMA Fungus. 2019 Jun 7;10:3. doi: 10.1186/s43008-019-0008-4 (PMC7325678; doi:10.1186/s43008-019-0008-4)
Supplement: Supplementary file 21 — Table S5. Summary of original sequencing data for Botryosphaeria dothidea LW-Hubei isolate generated using the PacBio platform. (DOCX 14 kb) [file 43008_2019_8_MOESM21_ESM.docx]

Additional file 21: **Table S5** Summary of original sequencing data for *Botryosphaeria dothidea* LW-Hubei isolate generated using the PacBio platform.

| Sample Name (#) | Sequencing technology | Insert size (bp) | ReadsType (#) | Number of Reads (#) | Total of Bases (bp) | Mean Length (bp) | N50 Length (bp) | N90 Length (bp) | Reads Quality (#) | |  |
| --- | --- | --- | --- | --- | --- | --- | --- | --- | --- | --- | --- |
| LW-Hubei | PacBio | 20KB | PolymeraseRead PreFilter | 302,187 | 4,076,018,530 | 13,488 | 20,616 | 7,342 | 0.84 |  | |
|  |  |  | PolymeraseRead PostFilter | 256,380 | 3,721,684,056 | 14,516 | 21,440 | 7,770 | 0.85 |  | |
|  |  |  | Subreads PreFilter | 533,574 | 3,954,831,039 | 7,411 | 9,652 | 4,691 | 0.85 |  | |
|  |  |  | Subreads PostFilter | 469,109 | 3,697,892,241 | 7,882 | 9,673 | 4,839 | 0.85 |  | |
