# Supplementary material for: Comprehensive analysis of full genome sequence and Bd-milRNA/target mRNAs to discover the mechanism of hypovirulence in Botryosphaeria dothidea strains on pear infection with BdCV1 and BdPV1
Source: IMA Fungus. 2019 Jun 7;10:3. doi: 10.1186/s43008-019-0008-4 (PMC7325678; doi:10.1186/s43008-019-0008-4)
Supplement: Supplementary file 22 — Table S6. Statistical analysis of basic RNA-seq and small RNA sequencing information corresponding to the LW-Hubei strain genome used in this study. (DOCX 15 kb) [file 43008_2019_8_MOESM22_ESM.docx]

Additional file 22: **Table S6** Statistical analysis of basic RNA-seq and small RNA sequencing information corresponding to the LW-Hubei strain genome used in this study.

| ***De novo* Sequencing** | **Statistics reads** | | **Strain name** | | | |
| --- | --- | --- | --- | --- | --- | --- |
|  |  |  | **LW-CP** | **LW-C** | **LW-P** | **Mock** |
| RNA-Seq sequencing | Total numbers | Total clean reads | 44,106,760 | 42,932,052 | 46022750 | 45713070 |
|  |  | Total Bases | 4,410,676,000 | 4,293,205,200 | 4602275,000 | 4571307000 |
|  | Mapping to genome | Total mapping Ratio | 90.22% | 90.16% | 90.77% | 91.04% |
|  |  | Uniquely mapping ratio | 86.17% | 86.31% | 86.55% | 86.09% |
|  | Mapping to gene  (percentage) | Total mapped reads (%) | 33297702 (75.49%) | 32622214 (75.99%) | 34752702 (75.51%) | 33933140 (74.23%) |
|  |  | Perfect matched reads (%) | 26999316 (61.21%) | 22852002(53.23%) | 24472689 (53.18%) | 23476579 (51.36%) |
|  |  | Unique matched reads (%) | 27872910 (63.19%) | 27296452 (63.58%) | 29394550 (63.87%) | 28473506 (62.29%) |
| Small RNA sequencing | Total numbers | Total sRNAs | 11221049 | 11837018 | 10949634 | 10,880,651 |
|  |  | Total unique sRNAs | 1223398 | 947838 | 1047920 | 1052904 |
|  | Mapping to genome (percentage) | Mapped total tags (%) | 1510678 (13.46%) | 935665 (7.9%) | 1737258 (15.87%) | 5537885 (50.9%) |
|  |  | Uniquely mapping reads (%) | 213250 (17.43%) | 132437 (13.97%) | 228277(21.87%) | 374422 (35.56%) |
