# Supplementary material for: Comprehensive analysis of full genome sequence and Bd-milRNA/target mRNAs to discover the mechanism of hypovirulence in Botryosphaeria dothidea strains on pear infection with BdCV1 and BdPV1
Source: IMA Fungus. 2019 Jun 7;10:3. doi: 10.1186/s43008-019-0008-4 (PMC7325678; doi:10.1186/s43008-019-0008-4)
Supplement: Supplementary file 23 — Table S7. Statistical analysis of specific motifs for transmethylases. (DOCX 19 kb) [file 43008_2019_8_MOESM23_ESM.docx]

Additional file 23: **Table S7** Statistics analysis of specific motifs for transmethylases.

|  | **Motif** | **Position** | **Type of modification** | **Fraction** | **nDetected** | **nGenome** | **meanScore** |
| --- | --- | --- | --- | --- | --- | --- | --- |
| **1** | DAGGYABTA | 1 | m6A | 0.20710 | 70 | 338 | 63.42857 |
|  | WNAGGCMGCA | 2 | m6A | 0.19651 | 45 | 229 | 58.71111 |
|  | GWDNNRNG | 0 | Modified base | 0.02149 | 2667 | 124131 | 44.16873 |
|  | TNNNDNNH | 0 | Modified base | 0.00677 | 7811 | 1153422 | 43.34720 |
| **2** | GNNNNNNH | 0 | Modified base | 0.08498 | 269156 | 3167308 | 39.38097 |
|  | GSNNNNNG | 0 | Modified base | 0.04034 | 28677 | 710895 | 38.52561 |
|  | CSVNDTBH | 0 | m4C | 0.03468 | 5553 | 160126 | 52.60238 |
|  | CNNNNVNH | 0 | m4C | 0.02926 | 72310 | 2471318 | 52.18070 |
|  | GWRVNVNG | 0 | modified_base | 0.02470 | 4297 | 173998 | 39.72143 |
|  | CSVNNNNG | 0 | m4C | 0.01400 | 7177 | 512608 | 54.95095 |
|  | CNNNNTNH | 0 | m4C | 0.01255 | 9134 | 727548 | 53.81323 |
|  | TNNNDNNH | 0 | Modified base | 0.00648 | 12216 | 1906362 | 40.07425 |
| **3** | DNNNNAGGYAGTA | 5 | m6A | 0.33108 | 49 | 148 | 63.93877 |
|  | AVNAGNTANTANNH | 3 | m6A | 0.19497 | 31 | 159 | 55.74194 |
|  | DAGGCMGCA | 1 | m6A | 0.19101 | 51 | 267 | 58.49020 |
|  | VNAGNTANTNNTNNAS | 2 | m6A | 0.16575 | 30 | 181 | 80.4 |
|  | ANNNNGNNCTTNAGNC | 12 | m6A | 0.16327 | 24 | 147 | 115.45834 |
|  | GWDNNRNG | 0 | Modified base | 0.02706 | 2,471 | 91314 | 45.24929 |
|  | TNNNDNNH | 0 | Modified base | 0.00907 | 7,720 | 850935 | 43.25777 |
| **4** | DAGGYAGYA | 1 | m6A | 0.20621 | 73 | 354 | 65.60274 |
|  | GWBNDNDG | 0 | Modified base | 0.01824 | 1889 | 103587 | 44.13340 |
|  | TNNNDNNH | 0 | Modified base | 0.00821 | 6786 | 826640 | 44.28824 |
| **5** | DAGGYASTA | 1 | m6A | 0.27451 | 56 | 204 | 61.71429 |
|  | ANNNNGNNCTTNAGNC | 12 | m6A | 0.15753 | 23 | 146 | 127.04348 |
|  | GWRVNVNG | 0 | Modified base | 0.02691 | 1896 | 70444 | 43.89768 |
|  | TNNNDNNH | 0 | Modified base | 0.00784 | 6113 | 779634 | 44.42352 |
| **6** | GNNNNNNH | 0 | Modified base | 0.08094 | 199986 | 2470892 | 39.1202 |
|  | HGSVVNNNG | 1 | Modified base | 0.05912 | 15180 | 256773 | 37.87727 |
|  | CNNNNNNH | 0 | m4C | 0.022622 | 56513 | 2498191 | 60.34008 |
|  | TNNNDNNH | 0 | Modified base | 0.00524 | 7716 | 1472042 | 42.46734 |
| **7** | DAGGYMGYA | 1 | m6A | 0.15295 | 145 | 948 | 53.33104 |
|  | GNNNNNNH | 0 | Modified base | 0.08323 | 178079 | 2139635 | 39.90947 |
|  | HGSVVNNNG | 1 | Modified base | 0.06114 | 13,645 | 223183 | 38.46853 |
|  | CNNNNNNH | 0 | m4C | 0.02509 | 54382 | 2167081 | 61.67153 |
|  | TNNNDNNH | 0 | Modified base | 0.00634 | 8058 | 1270217 | 42.72226 |
| **8** | AGGYANTANDNNNNNNNV | 0 | m6A | 0.3 | 54 | 180 | 67.16666 |
|  | DAGGYMRCA | 1 | m6A | 0.16176 | 66 | 408 | 65.16666 |
|  | HGWBNKNRGV | 1 | Modified base | 0.059474 | 1199 | 20160 | 43.31026 |
|  | GWRVNVNG | 0 | Modified base | 0.05270 | 2957 | 56105 | 41.41698 |
|  | TNNNDNNH | 0 | Modified base | 0.012890 | 7824 | 606719 | 41.98492 |
|  | TNNNCVNH | 0 | Modified base | 0.00547 | 1016 | 185893 | 38.66732 |
| **9** | DAGGYAGYA | 1 | m6A | 0.2225 | 89 | 400 | 55.29213 |
|  | TNNNDNNH | 0 | Modified base | 0.00728 | 7175 | 985542 | 43.29477 |

1~9 represent the 12 scaffolds data separated 9 segments data on average in the order 1, 2, 3, 4, 5, 6, 7, 8, 9, each of which possesses at least the whole one scaffold data; Position represent one normal base (A, T, C, G) position on the motif with the first value being 0, following by the order 1, 2, 3, 4 and so on.
