# Supplementary material for: Comprehensive analysis of full genome sequence and Bd-milRNA/target mRNAs to discover the mechanism of hypovirulence in Botryosphaeria dothidea strains on pear infection with BdCV1 and BdPV1
Source: IMA Fungus. 2019 Jun 7;10:3. doi: 10.1186/s43008-019-0008-4 (PMC7325678; doi:10.1186/s43008-019-0008-4)
Supplement: Supplementary file 24 — Table S8. The complete genome features of the eight fungi used as references in the comparative genome analyses. (DOCX 14 kb) [file 43008_2019_8_MOESM24_ESM.docx]

Additional file 24: **Table S8** The complete genome features of the eight fungi as references in the comparative genome analyses.

| Species（strain name） | Genome size (Mb) | | Protein coding genes | GC content (%) | NCBI accessions | Release/Update |
| --- | --- | --- | --- | --- | --- | --- |
| *Cenococcum geophilum* (1.58) | 177.56 | 14709 | | 37.5 | LKKR00000000 | 7/26/2016 |
| *Neofusicoccum parvum* (UCRNP2) | 42.59 | | 10366 | 56.7 | AORE00000000 | 8/4/2014 |
| *Macrophomina phaseolina* (MS6) | 48.88 | | 13806 | 52.3 | AHHD01000000 | 8/11/2014 |
| *Diplodia corticola*  (CBS 112549) | 34.99 | | 10839 | 57.1 | MNUE00000000 | 11/18/2016 |
| *Diplodia seriata* (F98.1) | 37.27 | | 8050 | 56.5 | MSZU01000000 | 1/31/2017 |
| *Dothistroma septosporum* (NZE10) | 30.21 | | 12415 | 53.1 | AIEN01000000 | 8/11/2014 |
| *Setosphaeria.turcica* | 43.01 | | 11698 | 51.4 | AIHT01000000 | 5/05/2014 |
| *Lepidopterella palustris*  (CBS 459.81) | 45.67 | | 13861 | 46.2 | LKAR01000000 | 7/26/2016 |
