# Supplementary material for: Comprehensive analysis of full genome sequence and Bd-milRNA/target mRNAs to discover the mechanism of hypovirulence in Botryosphaeria dothidea strains on pear infection with BdCV1 and BdPV1
Source: IMA Fungus. 2019 Jun 7;10:3. doi: 10.1186/s43008-019-0008-4 (PMC7325678; doi:10.1186/s43008-019-0008-4)
Supplement: Supplementary file 25 — Table S9. Different methods used for prediction of functional annotation of Botryosphaeria dothidea LW-Hubei isolate. (DOCX 13 kb) [file 43008_2019_8_MOESM25_ESM.docx]

Additional file 25: **Table S9** Different methods used for predication of functional annotation of *Botryospheria dothidea* LW-Hubei isolate.

| Utilized Database annotation | No. of genes annotated | Percentage of genes identified |
| --- | --- | --- |
| NCBI Nr database | 12273 | 87.09% |
| Swiss-Prot database | 3633 | 25.78% |
| GO database | 7558 | 53.63% |
| KEEG orthology database | 4523 | 32.09% |
| COG database | 3633 | 25.78% |
| KOG database | 2536 | 18% |
| T3SS database | 3833 | 27.2% |
| IPR database | 10336 | 73.35% |
| TREMBL database | 12280 | 87.14% |
| P450 database | 1454 | 10.31 |
| TF database | 573 | 4.06% |
| CAZY database | 351 | 2.49% |
| PHI database | 1096 | 7.77% |
| Total protein-coding gene numbers (14091) | 13135 | 93.2% |
