# Supplementary material for: Comprehensive analysis of full genome sequence and Bd-milRNA/target mRNAs to discover the mechanism of hypovirulence in Botryosphaeria dothidea strains on pear infection with BdCV1 and BdPV1
Source: IMA Fungus. 2019 Jun 7;10:3. doi: 10.1186/s43008-019-0008-4 (PMC7325678; doi:10.1186/s43008-019-0008-4)
Supplement: Supplementary file 27 — Table S11. Predicted effectors (n = 12) from Botryosphaeria dothidea LW-Hubei can be annotated by PHI. (DOCX 15 kb) [file 43008_2019_8_MOESM27_ESM.docx]

Additional file 27: **Table S11** Predicted effectors (n = 12) from *Botryosphaeria dothidea* LW-Hubei can be annotated by PHI.

| **Gene name** | **Length (nt)** | **Identity** | **E_value** | **PHI_id** | **Gene_name** | **PHI_name** | **Pathogen_Species** | **Mutant_Phenotype (Virulence)** |
| --- | --- | --- | --- | --- | --- | --- | --- | --- |
| GME7274_g | 401 | 49.02 | 1E-32 | PHI:1653 | GzWing026 | I1S2A0 | *Fusarium_graminearum* | unaffected_pathogenicity |
| GME7171_g | 643 | 40.43 | 5.00E-23 | PHI:2644 | thioredoxin_1 | P0AA28 | *Salmonella_enterica* | reduced_virulence |
| GME11462_g | 643 | 43.15 | 3.00E-41 | PHI:4490 | C2H2 | G5EGV6 | *Magnaporthe_oryzae* | increased_virulence_(hypervirulence) |
| GME9424_g | 653 | 50.7 | 2.00E-60 | PHI:1040 | Lmgpi15 | B5DBZ7 | *Leptosphaeria_maculans* | reduced_virulence |
| GME4517_g | 654 | 51.32 | 3.00E-66 | PHI:4925 | Hbr1 | Q8TG40 | *Candida_albicans* | reduced_virulence |
| GME8014_g | 657 | 59.02 | 3.00E-41 | PHI:2079 | Moatg12 | Q51P78 | *Magnaporthe_oryzae* | loss_of_pathogenicity |
| GME7363_g | 731 | 51.81 | 4.00E-46 | PHI:1549__PHI:2385 | MYT2 | I1RTN5 | *Fusarium_graminearum* | unaffected_pathogenicity__reduced_virulence |
| GME11318_g | 788 | 51.42 | 1.00E-80 | PHI:4845 | PL1332 | A0A0E3USI9 | *Alternaria_brassicicola* | reduced_virulence |
| GME3278_g | 789 | 89.44 | 1.00E-105 | PHI:1563 | GzOB003 | I1RAX7 | *Fusarium_graminearum* | unaffected_pathogenicity |
| GME4025_g | 840 | 50.56 | 3.00E-95 | PHI:2322 | SidH | Q4WF54 | *Aspergillus_fumigatus* | reduced_virulence |
| GME11118_g | 873 | 46.1 | 2.00E-85 | PHI:2322 | SidH | Q4WF54 | *Aspergillus_fumigatus* | reduced_virulence |
| GME13153_g | 875 | 40.08 | 1.00E-44 | PHI:3972 | CDA | L2FHG9 | *Colletotrichum_gloeosporioides* | unaffected_pathogenicity |
