# Supplementary material for: Comprehensive analysis of full genome sequence and Bd-milRNA/target mRNAs to discover the mechanism of hypovirulence in Botryosphaeria dothidea strains on pear infection with BdCV1 and BdPV1
Source: IMA Fungus. 2019 Jun 7;10:3. doi: 10.1186/s43008-019-0008-4 (PMC7325678; doi:10.1186/s43008-019-0008-4)
Supplement: Supplementary file 28 — Table S12. Annotated transporters (n = 552) of Botryosphaeria dothidea LW-Hubei isolate. (DOCX 13 kb) [file 43008_2019_8_MOESM28_ESM.docx]

Additional file 28: **Table S12** Annotated transporters (n = 552) of *Botryosphaeria dothidea* LW-Hubei isolate.

| Family ID | Family Description | Gene Number |
| --- | --- | --- |
| 1.A | Type Channels | 40 |
| 1.B | Barrel Porins | 6 |
| 1.C | Pore-Forming Toxins (Proteins and Peptides) | 1 |
| 1.F | Vesicle Fusion Pores | 2 |
| 1.H | Paracellular Channels | 2 |
| 1.I | Membrane-bounded Channels | 19 |
| 1.Q | Fungal Septal Pores | 1 |
| 2.A | Porters (uniporters, symporters, antiporters) | 229 |
| 2.D | Transcompartment Lipid Carrier | 2 |
| 3.A | P-P-bond-hydrolysis-driven transporters | 116 |
| 3.D | Oxidoreduction-driven transporters | 39 |
| 3.E | Light absorption-driven transporters | 2 |
| 4.D | Polysaccharide Synthase/Exporters | 5 |
| 4.E | Vacuolar Polyphosphate Polymerase-catalyzed Group Translocators | 1 |
| 5.B | Transmembrane 1-electron transfer carriers | 1 |
| 8.A | Auxiliary transport proteins | 29 |
| 9.A | Recognized transporters of unknown biochemical mechanism | 21 |
| 9.B | Putative transport proteins | 36 |
