# Supplementary material for: Comprehensive analysis of full genome sequence and Bd-milRNA/target mRNAs to discover the mechanism of hypovirulence in Botryosphaeria dothidea strains on pear infection with BdCV1 and BdPV1
Source: IMA Fungus. 2019 Jun 7;10:3. doi: 10.1186/s43008-019-0008-4 (PMC7325678; doi:10.1186/s43008-019-0008-4)
Supplement: Supplementary file 29 — Table S13. Predicted kinases (n = 128) from Botryosphaeria dothidea LW-Hubei isolate. (DOCX 14 kb) [file 43008_2019_8_MOESM29_ESM.docx]

Additional file 29: **Table S15** Predicted kinases (n = 128) from *Botryosphaeria dothidea* LW-Hubei isolate.

| **Kinase** | **Number** | **Gene list** |
| --- | --- | --- |
| AGC | 13 | GME752_g GME12818_g GME12944_g GME12957_g GME13828_g GME13849_g GME8009_g GME8778_g GME11020_g GME4057_g GME2095_g GME3540_g GME11197_g |
| Atypical | 14 | GME1096_g GME1552_g GME775_g GME13496_g GME13542_g GME13834_g GME8198_g GME6157_g GME10974_g GME12063_g GME7006_g GME4566_g GME5464_g GME2757_g |
| CAMK | 19 | GME1018_g GME1480_g GME933_g GME13378_g GME13906_g GME8418_g GME8865_g GME6393_g GME6716_g GME10632_g GME10639_g GME11845_g GME12198_g GME7387_g GME4237_g GME9407_g GME2231_g GME11230_g GME3073_g |
| CK1 | 2 | GME928_g GME8133_g |
| CMGC | 27 | GME1502_g GME154_g GME826_g GME82_g GME12963_g GME13065_g GME13697_g GME13704_g GME13862_g GME8050_g GME8569_g GME6789_g GME10306_g GME10785_g GME12389_g GME7217_g GME4141_g GME4249_g GME4595_g GME9153_g GME9319_g GME3503_g GME3790_g GME5110_g GME5405_g GME5411_g GME11564_g |
| Others | 24 | GME1046_g GME1205_g GME874_g GME12986_g GME13228_g GME13718_g GME8499_g GME8767_g GME8908_g GME10457_g GME10703_g GME11003_g GME12315_g GME4592_g GME4593_g GME4646_g GME9884_g GME1828_g GME2087_g GME2295_g GME3596_g GME3738_g GME3796_g GME11294_g |
| STE | 18 | GME1435_g GME325_g GME458_g GME13169_g GME8040_g GME8669_g GME6044_g GME6819_g GME10563_g GME4553_g GME9615_g GME9944_g GME1860_g GME2393_g GME3253_g GME5309_g GME12711_g GME12741_g |
| TK | 1 | GME4834_g |
| TKL | 10 | GME178_g GME14015_g GME9038_g GME5989_g GME6540_g GME7072_g GME4691_g GME9353_g GME2122_g GME4950_g |
