# Supplementary material for: Comprehensive analysis of full genome sequence and Bd-milRNA/target mRNAs to discover the mechanism of hypovirulence in Botryosphaeria dothidea strains on pear infection with BdCV1 and BdPV1
Source: IMA Fungus. 2019 Jun 7;10:3. doi: 10.1186/s43008-019-0008-4 (PMC7325678; doi:10.1186/s43008-019-0008-4)
Supplement: Supplementary file 30 — Table S14. Summary of accession numbers for RNAi components from the fungal strains analyzed in this study. (DOCX 15 kb) [file 43008_2019_8_MOESM30_ESM.docx]

Additional file 30: **Table S14** Summary of accession numbers for RNAi components from the fungal strains analyzed in this study.

| **Outline** | **Species (strain)** | **RNA dependent RNA polymerase (Rdrp)** | **Dicer(dcl)** | **Argonaute (ago)** |
| --- | --- | --- | --- | --- |
| ***Sordariomycetes*** | *Colletotrichum higginsianum* (IMI 349063) | CH1Rdrp1: CH063_02767 | CHdcl1: CH063_06582 | CHago1: CH063_04066 |
|  |  | CH2Rdrp2: CH063_05776 | CHdcl2: CH063_02619 | CHago2: CH063_09722 |
|  |  | CH3Rdrp3: CH063_08349 |  |  |
|  | *Colletotrichum graminicola* (M1.001) | CG1: GLRG_09527 | CG2: GLRG_05246 | CG1: GLRG_05439 |
|  |  | CG2: GLRG_02515 | CG1: GLRG_08364 | CG2: GLRG_06683 |
|  |  | CG3: GLRG_06276 |  |  |
|  | *Cryphonectria parasitica* (EP155) | CP (Rdrp1): CCV01471 | CP (dcl1): Q2VF19 | CP (ago1): ACY36939 |
|  |  | CP (Rdrp2): CCV01472 | CP (dcl2): Q2VF18 | CP (ago2): ACY36940 |
|  |  | CP (Rdrp3): CCV01473 |  | CP (ago3): ACY36941 |
|  |  |  |  | CP (ago4): ACY36942 |
|  | *Magnaporthe oryzae* (70-15) | MO1: MGG_13453 | MO (mdl1): MGG_01541 | MO1: MGG_01294 |
|  |  | MO2: MGG_02748 | MO (mdl2): MGG_12357 | MO2: MGG_13617 |
|  |  | MO3: MGG_06205 |  | MO3: MGG_14873 |
|  | *Neurospora crassa* (OR74A) | NC (sad1): NCU02178 | NC (dcl1): NCU08270 | NC (qde2): NCU04730 |
|  |  | NC (qde1): NCU07534 | NC (dcl2): NCU06766 | NC (sms2): NCU09434 |
|  |  | NC (Rdrp3): NCU08435 |  |  |
|  | *Fusarium graminearum* (PH-1) | FG (Rdrp1): FGSG_06504 | FG (dcl1): FGSG_09025 | FG (ago1): FGSG_08752 |
|  |  | FG (Rdrp2): FGSG_08716 | FG (dcl2): FGSG_04408 | FG (ago2): FGSG_00348 |
|  |  | FG (Rdrp3): FGSG_01582 |  |  |
| ***Dothideomycetes*** | *Mycosphaerella graminicola* (IPO323) | MG1: MYCGRDRAFT_117718 |  | MG1: MYCGRDRAFT_38035 |
|  |  | MG2: MYCGRDRAFT_51407 |  | MG2: MYCGRDRAFT_90232 |
|  |  | MG3: MYCGRDRAFT_49833 |  | MG3: MYCGRDRAFT_10621 |
|  | *Pyrenophora tritici-repentis* (Pt-1C-BFP) | PT1: PTRG_10475 | PT1: PTRG_05720 | PT1: PTRG_08652 |
|  |  | PT2: PTRG_07015 | PT2: PTRG_08809 | PT2: PTRG_01413 |
|  |  | PT3: PTRG_06422 |  | PT3: PTRG_06348 |
|  | *Leptosphaeria maculans* (JN3) | LM1: LEMA_P088990.1 | LM1: LEMA_P041660.1 | LM1: LEMA_P015760.1 |
|  |  | LM2: LEMA_P117350.1 | LM2: LEMA_P036310.1 | LM2: LEMA_P079240.1 |
|  |  | LM3: LEMA_P095020.1 |  | LM3: LEMA_P097140.1 |
| **Outgroups** | *Arabidopsis thaliana* (Col-0) | AT1: At4g11130 | AT-dcl1: (At1g01040) | AT(ago1): At1g48410) |
|  |  | AT2: At3g49500 | AT-dcl2: At3g0330 |  |
|  |  |  | AT-dcl3: At3g43920 |  |
|  |  |  | AT-dcl4: AT5G20320 |  |

Accession numbers: Locus tag
