# Supplementary material for: Comprehensive analysis of full genome sequence and Bd-milRNA/target mRNAs to discover the mechanism of hypovirulence in Botryosphaeria dothidea strains on pear infection with BdCV1 and BdPV1
Source: IMA Fungus. 2019 Jun 7;10:3. doi: 10.1186/s43008-019-0008-4 (PMC7325678; doi:10.1186/s43008-019-0008-4)
Supplement: Supplementary file 31 — Table S15. Summary of common and specific sRNA sequences and mean frequencies in the (a) LW-CP and Mock libraries, (b) LW-P and Mock libraries, and (c) LW-C and Mock libraries constructed from Botryosphaeria dothidea strains. (DOCX 15 kb) [file 43008_2019_8_MOESM31_ESM.docx]

| Libraries | Class | Unique sRNAs | Percent (%) | Total sRNAs | Percent (%) | Mean frequency |
| --- | --- | --- | --- | --- | --- | --- |
| (a) LW-CP and Mock | Total sRNAs | 2096857 | 100.00% | 22101700 | 100.00% | 10.54 |
|  | Mock_&_LW-CP | 179445 | 8.56% | 14274078 | 64.58% | 79.54 |
|  | Mock_specific | 873459 | 41.66% | 2202789 | 9.97% | 2.52 |
|  | LW-CP_specific | 1043953 | 49.79% | 5624833 | 25.45% | 5.39 |
| (b) LW-P and Mock | Total sRNAs | 1843817 | 100.00% | 21830302 | 100.00% | 11.84 |
|  | Mock & LW-P | 257007 | 13.94% | 16222169 | 74.31% | 63.12 |
|  | Mock_specific | 795897 | 43.17% | 1337739 | 6.13% | 1.68 |
|  | LW-P_specific | 790913 | 42.90% | 4270394 | 19.56% | 5.4 |
| (c) LW-C and Mock | Total sRNAs | 1900170 | 100.00% | 22717669 | 100.00% | 11.96 |
|  | Mock_&_LW-C | 100572 | 5.29% | 12674908 | 55.79% | 126.03 |
|  | Mock_specific | 952332 | 50.12% | 3509738 | 15.45% | 3.69 |
|  | LW-C_specific | 84726 | 44.59% | 6533023 | 28.76% | 77.11 |

Additional file 31: **Table S15** Summary of common and specific sRNA sequences and mean frequencies in the (a) LW-CP and Mock libraries, (b) LW-P and Mock libraries, and (c) LW-C and Mock libraries constructed from *Botryosphaeria dothidea* strains.
