# Supplementary material for: Comprehensive analysis of full genome sequence and Bd-milRNA/target mRNAs to discover the mechanism of hypovirulence in Botryosphaeria dothidea strains on pear infection with BdCV1 and BdPV1
Source: IMA Fungus. 2019 Jun 7;10:3. doi: 10.1186/s43008-019-0008-4 (PMC7325678; doi:10.1186/s43008-019-0008-4)
Supplement: Supplementary file 32 — Table S16. The 62 candidate novel Bd-milRNAs and six novel Bd-milRNAs among the milRNA*s detected in Botryosphaeria dothidea strains. (DOCX 32 kb) [file 43008_2019_8_MOESM32_ESM.docx]

Additional file 32: **Table S16** The 62 candidate novel *Bd*-milRNAs and six novel *Bd*-milRNAs among the milRNA*s detected in *Botryosphaeria dothidea* strains.

| miRNA name | Length of mature (nt) | Mature sequence(5'-3') | Counts of miRNAs/miRNA*s | | | | Precursor location | Length of Precursor (nt) | Mature sequence locus(Arm) | MFE (kcal/mol) |
| --- | --- | --- | --- | --- | --- | --- | --- | --- | --- | --- |
|  |  |  | Mock | LW-P | LW-C | LW-CP |  |  |  |  |

| *Bd*-milR1 |  | 22 | TAGCCTGTAGGTTCGCCTGATC | 18 | 10 | 5 | / | Scaffold10:1633704:1633782:+ | 79 | 5 | -41.70 |
| --- | --- | --- | --- | --- | --- | --- | --- | --- | --- | --- | --- |
| *Bd*-milR10 |  | 23 | AAGTGTACTCGTAGCCTTCTCCT | / | / | 5 | / | Scaffold5:1165876:1166229:- | 354 | 3 | -136.4 |
| *Bd*-milR11 |  | 23 | CATCTGGGCGTGTGGAAAAGAGT | / | / | 7 | / | Scaffold5:2014394:2014492:- | 99 | 5 | -26.2 |
| *Bd*-milR12 |  | 21 | ACGCTCCCGAAGGAACTGGCT | / | / | 141 | / | Scaffold5:2151747:2152027:- | 281 | 5 | -108 |
| *Bd*-milR13 |  | 21 | ATCGGTATTCGTCTTTCTAGT | / | / | 7 | / | Scaffold6:224674:224859:- | 186 | 3 | -63.8 |
| *Bd*-milR14 |  | 24 | TGGGGCGGCTCTCTGTTTCGGTTG | / | / | 6 | / | Scaffold6:2305024:2305111:- | 88 | 5 | -35.3 |
| *Bd*-milR15 |  | 23 | TACGAGGACCAATTCATAGAATT | / | / | 11 | / | Scaffold7:1816600:1816711:+ | 112 | 5 | -32.4 |
| *Bd*-milR16 |  | 23 | GACGACGACGACTTCACGACAGC | / | / | 10 | / | Scaffold8:2589431:2589572:+ | 142 | 5 | -30.4 |
| *Bd*-milR17 |  | 21 | TGTCCAAGCTCAGAATCCCTT | / | / | 10 | / | Scaffold8:2982983:2983120:+ | 138 | 5 | -52.7 |
| *Bd*-milR18 |  | 21 | GTGGTGTGGTGTAGTGGTAGC | / | 19 | 15 | / | Scaffold8:1823598:1823723:- | 126 | 3 | -27.5 |
| *Bd*-milR19 |  | 21 | TTTCTTGGCTTCCGAAGTAGA | / | / | 9 | / | Scaffold9:1073253:1073536:+ | 284 | 5 | -83 |
| *Bd*-milR2 |  | 22 | TATAATGCTCTGATCCGCACAC | / | / | 5 | / | Scaffold12:1555049:1555238:- | 190 | 5 | -55.7 |
| *Bd*-milR21 |  | 24 | TGGGTTCTGGGGCTTGGGGTTTTG | / | / | 7 | / | Scaffold9:937592:937732:- | 141 | 5 | -41.2 |
| *Bd*-milR22 |  | 21 | TTGACAATTTTACGCTTTAAC | / | / | / | 5 | Scaffold10:2270950:2271202:+ | 253 | 3 | -71.3 |
| *Bd*-milR23 |  | 22 | ATTGACTTTCACTTCGCTGCTC | / | / | / | 8 | Scaffold1:398649:399019:- | 371 | 3 | -151.6 |
| *Bd*-milR24 |  | 22 | TAAGTCAGCTTGGACAAGACGA | / | / | / | 149 | Scaffold1:913528:913636:- | 109 | 5 | -56.5 |
| *Bd*-milR25 |  | 20 | TGGGCGGGCGGGCATGGATA | / | / | / | 8 | Scaffold1:4216847:4217155:- | 309 | 3 | -110 |
| *Bd*-milR26 |  | 22 | GGAGGAGAAGCCCGTCGGCGGC | / | / | / | 5 | Scaffold2:3431508:3431690:+ | 183 | 3 | -58 |
| *Bd*-milR27 |  | 21 | TAGGGAGGATGGGCGGGATCT | / | / | / | 8 | Scaffold2:2838710:2838913:- | 204 | 5 | -54 |
| *Bd*-milR28 |  | 22 | TATTTCGGCGTCACAACTCTTC | / | / | / | 5 | Scaffold2:2885287:2885353:- | 67 | 5 | -24.2 |
| *Bd*-milR3 |  | 21 | TTTGCTCAGTCTGGAAGTATG | / | / | 30 | / | Scaffold16:369863:370000:- | 138 | 5 | -43.4 |
| *Bd*-milR30 |  | 22 | TGTATTCCCGCTCTTGCCATTC | / | / | / | 5 | Scaffold3:609050:609351:- | 302 | 5 | -93.7 |
| *Bd*-milR31 |  | 22 | TATCTATCTAAAAACTCGCTCT | / | / | / | 6 | Scaffold40:14280:14635:+ | 356 | 3 | -43 |
| *Bd*-milR32 |  | 21 | TACACGAAGGTCAAGCTGGGG | / | / | / | 14 | Scaffold4:2149480:2149587:+ | 108 | 3 | -38.1 |
| *Bd*-milR33 |  | 20 | CTGGGTGGGCGGGATTCCTG | / | / | / | 42 | Scaffold5:664418:664498:- | 81 | 5 | -33.2 |
| *Bd*-milR34 |  | 21 | GACACTAATTAGATCTGACAT | / | / | / | 8 | Scaffold6:133074:133168:+ | 95 | 5 | -21.4 |
| *Bd*-milR35 |  | 22 | ACGCCCGCCTGTATCACTGTCC | / | / | / | 5 | Scaffold6:459925:460140:- | 216 | 3 | -85.6 |
| *Bd*-milR36 |  | 21 | TAGGACTAGTGCTGCTTCACC | / | / | / | 7 | Scaffold7:1377467:1377805:+ | 339 | 5 | -121.1 |
| *Bd*-milR37 |  | 24 | TCGAGTACTCTGAGATCAGCGAGG | / | / | / | 7 | Scaffold7:3043534:3043635:+ | 102 | 5 | -40.8 |
| *Bd*-milR38 |  | 22 | TGCGGTTGAGGGACGAGGCAGA | / | / | / | 5 | Scaffold9:2604984:2605171:+ | 188 | 5 | -60 |
| *Bd*-milR39 |  | 20 | TTCGAGTAGGGATTGTAGCT | / | 7 | / | / | Scaffold11:17941:18062:- | 122 | 5 | -33.4 |
| *Bd*-milR39 |  | 20 | TTCGAGTAGGGATTGTAGCT | / | 7 | / | / | Scaffold21:137767:137888:+ | 122 | 5 | -33.4 |
| *Bd*-milR4 |  | 21 | TACCGGTCGCGACAGGCTTGC | / | / | 19 | / | Scaffold1:1715412:1715484:+ | 73 | 5 | -34.4 |
| *Bd*-milR40 |  | 21 | TTGACGTGATGATTAATGACT | / | 9 | / | / | Scaffold12:865558:865704:- | 147 | 5 | -32.5 |
| *Bd*-milR41 |  | 20 | AGATATGTTGTAGAGCGCGG | / | 6 | / | / | Scaffold15:730446:730548:+ | 103 | 5 | -42.4 |
| *Bd*-milR41 |  | 20 | AGATATGTTGTAGAGCGCGG | / | 6 | / | / | Scaffold18:9129:9228:- | 100 | 5 | -44.5 |
| *Bd*-milR41 |  | 20 | AGATATGTTGTAGAGCGCGG | / | 6 | / | / | Scaffold4:1161704:1161803:+ | 100 | 5 | -40.9 |
| *Bd*-milR42 |  | 21 | TAACATATCTCGATCTGACGC | / | 8 | / | / | Scaffold22:56808:56901:+ | 94 | 3 | -26.3 |
| *Bd*-milR43 |  | 21 | TGGCTGCCACTCTGTAGTTTG | / | 5 | / | / | Scaffold2:1699494:1699578:+ | 85 | 3 | -29.8 |
| *Bd*-milR44 |  | 21 | TTCGATCCCGGGTTGGCGCTT | / | 28 | / | / | Scaffold2:3025281:3025568:- | 141 | 3 | -141.3 |
| *Bd*-milR45 |  | 21 | TACACGGGCTGCGAGGGGATC | / | 24 | / | / | Scaffold36:2541:2651:+ | 111 | 5 | -44.7 |
| *Bd*-milR46 |  | 20 | AAGGTCTGAACCCTGTGGTT | 18 | 21 | / | / | Scaffold6:1237884:1238021:+ | 138 | 3 | -45.8 |
| *Bd*-milR47 |  | 21 | TAAGAATTCTATCGCATATCG | / | 26 | / | / | Scaffold6:418265:418350:- | 86 | 5 | -24.4 |
| *Bd*-milR48 |  | 23 | CGCTGGATCGTGTGACGTTGATT | / | 6 | / | / | Scaffold7:206203:206342:+ | 140 | 5 | -51.4 |
| *Bd*-milR5 |  | 23 | TGGACAACTCTGATGGCTTTGAC | / | / | 5 | / | Scaffold1:2992817:2992916:+ | 100 | 5 | -24.9 |
| *Bd*-milR50 |  | 21 | AAGGGCTTCTTTCCTCTCAGG | / | 5 | / | / | Scaffold7:2834615:2834961:+ | 347 | 5 | -97.2 |
| *Bd*-milR51 |  | 22 | TGGATTTGAGGACATGGCTGGA | / | 6 | / | / | Scaffold8:1869994:1870222:- | 229 | 3 | -100.7 |
| *Bd*-milR52 |  | 21 | TACGGTGCATTTAGAACGGTG | 5 | / | / | / | Scaffold11:9095:9199:- | 105 | 3 | -40.3 |
| *Bd*-milR52 |  | 21 | TACGGTGCATTTAGAACGGTG | 5 | / | / | / | Scaffold22:62709:62813:- | 105 | 3 | -40.3 |
| *Bd*-milR52 |  | 21 | TACGGTGCATTTAGAACGGTG | 5 | / | / | / | Scaffold27:36734:36838:+ | 105 | 3 | -40.3 |
| *Bd*-milR52 |  | 21 | TACGGTGCATTTAGAACGGTG | 5 | / | / | / | Scaffold28:164:268:+ | 105 | 3 | -40.3 |
| *Bd*-milR52 |  | 21 | TACGGTGCATTTAGAACGGTG | 5 | / | / | / | Scaffold34:27162:27266:- | 105 | 3 | -38.3 |
| *Bd*-milR52 |  | 21 | TACGGTGCATTTAGAACGGTG | 5 | / | / | / | Scaffold5:4076:4180:- | 105 | 3 | -40.3 |
| *Bd*-milR53 |  | 23 | TCTTCGATGGTCTAGTGGTCATG | 2098 | / | / | / | Scaffold11:2538312:2538497:- | 186 | 5 | -65.8 |
| *Bd*-milR54 |  | 21 | ATGACCACTAGACCATCGAAG | 11 | / | / | / | Scaffold12:2032968:2033050:+ | 83 | 5 | -24.5 |
| *Bd*-milR56 |  | 20 | CTCTTATTGTCAGTCGCAGC | 5 | / | / | / | Scaffold2:3927053:3927208:+ | 156 | 3 | -52.6 |
| *Bd*-milR57 |  | 20 | TGGTACTGTACGTATTGACG | 5 | / | / | / | Scaffold3:1844605:1844958:+ | 354 | 5 | -142.2 |
| *Bd*-milR58 |  | 21 | GCAGGAATGATAGACTCAAGC | 6 | / | / | / | Scaffold4:3098737:3098805:+ | 69 | 3 | -31.7 |
| *Bd*-milR6 |  | 21 | TTTCGGGACTTCGTACTGACC | / | 12 | / | / | Scaffold1:3086995:3087154:- | 160 | 3 | -49.9 |
| *Bd*-milR6 |  | 21 | TTTCGGGACTTCGTACTGACC | / | / | 12 | / | Scaffold1:3086995:3087154:- | 160 | 3 | -49.9 |
| *Bd*-milR60 |  | 21 | AGGCGGCTTGAATGTGTGATG | 14 | / | / | / | Scaffold5:1437294:1437573:- | 280 | 5 | -90.5 |
| *Bd*-milR61 |  | 21 | TCTCCGACGGGGAATCGAACC | 7 | / | / | / | Scaffold5:3158009:3158298:- | 290 | 3 | -92.9 |
| *Bd*-milR62 |  | 20 | CATTTCCGTTTGGCTCGCTT | 5 | / | / | / | Scaffold6:1747509:1747599:+ | 91 | 3 | -21.7 |
| *Bd*-milR63 |  | 21 | TACGAAAGGTGCTGGTTGTGG | 5 | / | / | / | Scaffold6:2991249:2991372:+ | 124 | 5 | -51.2 |
| *Bd*-milR64 |  | 22 | TAAGAATTCTATCGCATATCGC | 46 | / | / | / | Scaffold6:418265:418350:- | 86 | 5 | -24.4 |
| *Bd*-milR66 |  | 21 | TTCGAGGATCTGCATCGCTCC | 5 | / | / | / | Scaffold7:1051890:1052001:- | 112 | 5 | -39.6 |
| *Bd*-milR67 |  | 21 | TGTGTCGGGCTTGCCGCTGTC | 5 | / | / | / | Scaffold8:2847825:2847904:+ | 80 | 5 | -31.6 |
| *Bd*-milR68 |  | 21 | TTAGTCCCTATGGTGTAGATATC | 6 | / | / | / | Scaffold8:2257898:2258140:- | 243 | 3 | -69.9 |
| *Bd*-milR7 |  | 23 | TAGGAATCTTTGAATTGAAAGGA | / | / | 10 | / | Scaffold2:1408902:1409183:- | 282 | 5 | -77.2 |
| *Bd*-milR8 |  | 23 | TTTCTCGGCTGCTTTGCTTACTG | / | / | 6 | 5 | Scaffold2:1804218:1804360:- | 143 | 5 | -51.9 |
| *Bd*-milR9 |  | 21 | TCGAACTGGGGACCTCGGGAT | / | / | 158 | 69 | Scaffold2:3025296:3025551:- | 256 | 5 | -108.7 |
| *Bd*-milR20 |  | 21 | TGGGCAGTCTGTGCGTGAGCT | / | / | 7/2 | / | Scaffold9:1457883:1457971:+ | 89 | 5 | -38.7 |
| *Bd*-milR29 |  | 22 | TTCCTCGCCTCCGCTGCTGTAC | / | / | / | 5/1 | Scaffold3:3289852:3290061:+ | 210 | 5 | -40.5 |
| *Bd*-milR49 |  | 20 | TGCGGTCTAGAGAAAATCCT | / | 5/1 | / | / | Scaffold7:2348416:2348593:+ | 178 | 5 | -34.3 |
| *Bd*-milR55 |  | 23 | TATCTCGCCCGGCTTCTTATTGG | 12/1 | / | / | / | Scaffold1:87072:87213:- | 142 | 5 | -51.8 |
| *Bd*-milR59 |  | 22 | ATTGTGTTTGCAGAGCTGGAGC | 9/3 | / | / | / | Scaffold5:1182585:1182721:+ | 137 | 5 | -56.3 |
| *Bd*-milR65 |  | 20 | AAGAACTTTTGAGACCCACT | 23/17 | / | / | / | Scaffold7:1482111:1482246:+ | 136 | 3 | -68.9 |
