# Supplementary material for: Comprehensive analysis of full genome sequence and Bd-milRNA/target mRNAs to discover the mechanism of hypovirulence in Botryosphaeria dothidea strains on pear infection with BdCV1 and BdPV1
Source: IMA Fungus. 2019 Jun 7;10:3. doi: 10.1186/s43008-019-0008-4 (PMC7325678; doi:10.1186/s43008-019-0008-4)
Supplement: Supplementary file 33 — Table S17. The differentially expressed known Bd-milRNAs in the LW-CP/Mock, LW-C/Mock and LW-P/Mock libraries constructed from Botryosphaeria dothidea strains by sRNA sequencing. (DOCX 29 kb) [file 43008_2019_8_MOESM33_ESM.docx]

Additional file 33: **Table S****17** The differentially expressed known *Bd*-milRNAs in the LW-CP/Mock, LW-C/Mock and LW-P/Mock libraries constructed from *Botryosphaeria dothidea* strains by sRNA sequencing.

| *Bd*-milRNA name | Expressed counts in each library  -expressed  -expressed  -expressed | | | |  | Log2fold change b/p-value |  |
| --- | --- | --- | --- | --- | --- | --- | --- |
|  | LW-CP | LW-C | LW-P | Mock | LW-CP/Mock | LW-C/ Mock | LW-P/ Mock |
| *Bd*-milR1061-3p | 0 | 0 | 0 | 114 | -10.03/8.0795e-36 | -10.03/3.4237e-37 | -10.03/3.3455e-35 |
| *Bd*-milR1077-5p | 0 | 0 | 0 | 1100 | -13.30/0 | -13.30/0 | -13.30/0 |
| *Bd*-milR1086 | 0 | 0 | 0 | 28 | -8.007/2.3750e-09 | -8.007/1.0702e-09 | -8.007/3.3985e-9 |
| *Bd*-milR1122b-3p | 0 | 0 | 0 | 77 | -9.466/1.9720e-24 | -9.466/2.3106e-25 | -9.466/5.1695e-24 |
| *Bd*-milR1134 | 198 | 0 | 914 | 3961 | -4.366/0 | -15.15/0 | -2.124/0 |
| *Bd*-milR1510a-5p | 0 | 0 | 0 | 442 | -11.98/9.0900e-137 | -11.98/4.6772e-142 | -11.98/2.1660e-134 |
| *Bd*-milR1523a | 0 | 0 | 0 | 2173 | -14.28/0 | -14.28/0 | -14.28/0 |
| *Bd*-milR156g | 0 | 0 | 0 | 1071 | -13.26/0 | -13.26/0 | -13.26/0 |
| *Bd*-milR157a-5p | 0 | 0 | 322 | 2736 | -14.61/0 | -14.61/0 | -3.096/0 |
| *Bd*-milR160g | 0 | 0 | 1055 | 3204 | -14.84/0 | -14.84/0 | -1.611/7.0842e-252 |
| *Bd*-milR166c | 0 | 0 | 4124 | 32427 | -18.18/0 | -18.18/0 | -2.984/0 |
| *Bd*-milR167c-5p | 0 | 172 | 1379 | 16116 | -17.17/0 | -6.671/0 | -3.555/0 |
| *Bd*-milR167e-3p | 0 | 0 | 612 | 1491 | -13.74/0 | -13.74/0 | -1.293/1.4570e-85 |
| *Bd*-milR168a | 20724 | 1482 | 16458 | 0 | 17.494/0 | 13.611/0 | 17.197/0 |
| *Bd*-milR169h | 0 | 0 | 0 | 422 | -11.92/1.3001e-130 | -11.92/1.1592e-135 | -11.92/2.4197e-128 |
| *Bd*-milR171e-5p | 0 | 0 | 0 | 28 | -8.007/2.3750e-09 | -8.007/1.0702e-09 | -8.007/3.3985e-9 |
| *Bd*-milR172c-3p | 17008 | 0 | 13822 | 2148 | 2.9407/0 | -14.26/0 | 2.6767/0 |
| *Bd*-milR2094-5p | 541 | 0 | 651 | 6195 | -3.561/0 | -15.79/0 | -3.259/0 |
| *Bd*-milR2654 | 0 | 0 | 0 | 1922 | -14.10/0 | -14.10/0 | -14.10/0 |
| *Bd*-milR3627-5p | 0 | 0 | 0 | 874 | -12.97/1.0036e-269 | -12.97/3.5954e-280 | -12.97/4.9747e-265 |
| *Bd*-milR393d | 102 | 0 | 169 | 348 | -1.814/2.3261e-34 | -11.64/5.2799e-112 | -1.051/1.0873e-15 |
| *Bd*-milR395b | 0 | 0 | 0 | 139 | -10.31/1.6334e-43 | -10.31/3.4816e-45 | -10.31/9.2118e-43 |
| *Bd*-milR399f | 0 | 0 | 83 | 173 | -10.63/5.6093e-54 | -10.63/4.6955e-56 | -1.068/1.0502e-8 |
| *Bd*-milR408a-5p | 0 | 0 | 0 | 3096 | -14.79/0 | -14.79/0 | -14.79/0 |
| *Bd*-milR477d | 0 | 0 | 423 | 1318 | -13.56/0 | -13.56/0 | -1.648/3.5344e-108 |
| *Bd*-milR482d-5p | 0 | 0 | 0 | 341 | -11.61/1.1051e-105 | -11.61/9.1325e-110 | -11.61/7.5607e-104 |
| *Bd*-milR5080 | 0 | 0 | 0 | 50463 | -18.82/0 | -18.82/0 | -18.82/0 |
| *Bd*-milR5182 | 0 | 0 | 0 | 1276 | -13.51/0 | -13.51/0 | -13.51/0 |
| *Bd*-milR533b-5p | 868 | 806 | 0 | 1905 | -1.178/1.7819e-95 | -1.362/6.9009e-123 | -14.09/0 |
| *Bd*-milR535b | 0 | 0 | 0 | 3225 | -14.85/0 | -14.85/0 | -14.85/0 |
| *Bd*-milR5644 | 0 | 0 | 0 | 1304 | -13.54/0 | -13.54/0 | -13.54/0 |
| *Bd*-milR5770a | 0 | 0 | 636 | 1993 | -14.16/0 | -14.16/0 | -1.656/9.5440e-164 |
| *Bd*-milR5820 | 0 | 0 | 0 | 4248 | -15.25/0 | -15.25/0 | -15.25/0 |
| *Bd*-milR6021 | 0 | 0 | 0 | 6088 | -15.77/0 | -15.77/0 | -15.77/0 |
| *Bd*-milR6022 | 0 | 0 | 37 | 185 | -10.73/1.1367e-57 | -10.73/6.8419e-60 | -2.331/2.7220e-25 |
| *Bd*-milR6136a.1 | 0 | 0 | 0 | 1854 | -14.05/0 | -14.05/0 | -14.05/0 |
| *Bd*-milR6145d | 40670 | 4936 | 38784 | 0 | 18.467/0 | 15.347/0 | 18.434/0 |
| *Bd*-milR6149a | 0 | 0 | 0 | 68 | -9.287/1.1610e-21 | -9.287/1.7422e-22 | -9.287/2.7232e-21 |
| *Bd*-milR6196 | 510 | 0 | 308 | 3341 | -2.756/0 | -14.90/0 | -3.448/0 |
| *Bd*-milR7539 | 0 | 0 | 709 | 2514 | -14.49/0 | -14.49/0 | -1.835/1.8647e-237 |
| *Bd*-milR7540a | 0 | 0 | 0 | 20 | -7.522/6.8840e-07 | -7.522/3.8649e-07 | -7.522/8.9233e-7 |
| *Bd*-milR7722-5p | 0 | 0 | 764 | 8481 | -16.25/0 | -16.25/0 | -3.481/0 |
| *Bd*-milR7732-3p | 394 | 0 | 467 | 957 | -1.324/2.3483e-58 | -13.10/1.0469e-306 | -1.044/5.7768e-40 |
| *Bd*-milR7761-3p | 23719 | 610 | 18170 | 0 | 17.689/0 | 12.331/2.0597e-173 | 17.340/0 |
| *Bd*-milR7815 | 0 | 0 | 0 | 1101 | -13.30/0 | -13.30/0 | -13.30/0 |
| *Bd*-milR7816 | 0 | 0 | 0 | 4031 | -15.17/0 | -15.17/0 | -15.17/0 |
| *Bd*-milR7821 | 21 | 0 | 72 | 176 | -3.111/5.4951e-33 | -10.65/5.1588e-57 | -1.298/1.584e-11 |
| *Bd*-milR812k | 0 | 0 | 0 | 115 | -10.04/3.9775e-36 | -10.04/1.6398e-37 | -10.04/1.6675e-35 |
| *Bd*-milR8141 | 0 | 0 | 0 | 34 | -8.287/3.3810e-11 | -8.287/1.2918e-11 | -8.287/5.2102e-11 |
| *Bd*-milR854a | 18 | 0 | 24 | 99 | -2.503/1.5864e-15 | -9.829/2.1386e-32 | -2.053/2.5051e-12 |
| *Bd*-milR8678 | 264 | 0 | 1082 | 2694 | -3.395/0 | -14.59/0 | -1.325/9.1317e-159 |
| *Bd*-milR8775 | 0 | 0 | 0 | 184 | -10.72/2.3090e-57 | -10.72/1.4285e-59 | -10.72/2.2705e-56 |
| *Bd*-milR902a-3p | 0 | 0 | 0 | 315 | -11.49/1.1103e-97 | -11.49/1.8750e-101 | -11.49/5.5092e-96 |
| *Bd*-milR912 | 0 | 0 | 0 | 73 | -9.389/3.3573e-23 | -9.389/4.3910e-24 | -9.389/8.3766e-23 |
| *Bd*-milR9722 | 0 | 0 | 0 | 491 | -12.13/7.5473e-152 | -12.13/1.0098e-157 | -12.13/3.2948e-149 |
| *Bd*-milR9762 | 0 | 0 | 0 | 38288 | -18.42/0 | -18.42/0 | -18.42/0 |
| *Bd*-milR9778 | 0 | 0 | 203 | 2756 | -14.62/0 | -14.62/0 | -3.772/0 |
| *Bd*-milR1023b-3p | 0 | 0 | 229 | 349 | -11.64/3.8128e-108 | -11.64/2.5288e-112 |  |
| *Bd*-milR1535a | 0 | 8837 | 96 | 103 | -9.886/1.9627e-32 | 6.3013/0 | / |
| *Bd*-milR163 | 0 | 0 | 482 | 812 | -12.86/1.2116e-250 | -12.86/2.3861e-260 | / |
| *Bd*-milR164b | 179 | 449 | 0 | 0 | 10.639/2.0437e-53 | 11.889/7.8772e-128 | / |
| *Bd*-milR166a-3p | 14 | 21 | 0 | 0 | 6.9631/0 | 7.4709/1.1812e-06 | / |
| *Bd*-milR172a | 357 | 0 | 1551 | 958 | -1.468/5.1504e-68 | -13.10/5.0142e-307 | / |
| *Bd*-milR2082 | 0 | 0 | 406 | 386 | -11.79/1.5621e-119 | -11.79/3.7470e-124 | / |
| *Bd*-milR2109-3p | 308 | 2115 | 0 | 0 | 11.422/2.1569e-91 | 14.125/0 | / |
| *Bd*-milR2623 | 3658 | 39261 | 0 | 0 | 14.992/0 | 18.339/0 | / |
| *Bd*-milR2624 | 155 | 2268 | 0 | 0 | 10.431/2.3759e-46 | 14.225/0 | / |
| *Bd*-milR2925 | 0 | 0 | 201 | 295 | -11.40/1.5880e-91 | -11.40/4.6472e-95 | / |
| *Bd*-milR3624-5p | 564 | 10620 | 0 | 0 | 12.295/9.3201e-167 | 16.453/0 | / |
| *Bd*-milR390b-5p | 0 | 0 | 15710 | 28478 | -17.99/0 | -17.99/0 | / |
| *Bd*-milR5014b | 0 | 0 | 1020 | 1591 | -13.83/0 | -13.83/0 | / |
| *Bd*-milR5038a | 0 | 0 | 254 | 260 | -11.22/9.3940e-81 | -11.22/7.1946e-84 | / |
| *Bd*-milR5255 | 0 | 0 | 728 | 685 | -12.62/1.4799e-211 | -12.62/9.5660e-220 | / |
| *Bd*-milR5260 | 0 | 0 | 489 | 466 | -12.06/3.7330e-144 | -12.06/9.9306e-150 | / |
| *Bd*-milR529 | 511 | 11788 | 0 | 0 | 12.152/3.7343e-151 | 16.603/0 | / |
| *Bd*-milR5534a | 65 | 0 | 453 | 339 | -2.427/6.6186e-48 | -11.60/3.9811e-109 | / |
| *Bd*-milR6034 | 2906 | 0 | 5481 | 7634 | -1.437/0 | -16.09/0 | / |
| *Bd*-milR6172 | 0 | 0 | 883 | 803 | -12.84/7.1335e-248 | -12.84/1.7992e-257 | / |
| *Bd*-milR6195 | 772 | 2365 | 0 | 0 | 12.748/5.4430e-228 | 14.286/0 | / |
| *Bd*-milR6441 | 0 | 0 | 945 | 1211 | -13.44/0 | -13.44/0 | / |
| *Bd*-milR7807b-3p | 177 | 5302 | 0 | 0 | 10.623/7.9289e-53 | 15.450/0 | / |
| *Bd*-milR8039 | 1814 | 3268 | 0 | 0 | 13.980/0 | 14.752/0 | / |
| *Bd*-milR815a | 196 | 0 | 269 | 440 | -1.211/2.5539e-24 | -11.98/2.0389e-141 | / |
| *Bd*-milR835-3p | 0 | 0 | 226 | 301 | -11.43/2.2607e-93 | -11.43/5.6097e-97 | / |
| *Bd*-milR8584 | 961 | 3413 | 0 | 0 | 13.064/1.2465e-283 | 14.815/0 | / |
| *Bd*-milR8635 | 1488 | 274 | 6507 | 10208 | -2.822/0 | -5.340/0 | / |
| *Bd*-milR9478-3p | 48 | 0 | 519 | 401 | -3.106/4.2723e-73 | -11.84/5.9987e-129 | / |
| *Bd*-milR948 | 0 | 0 | 604 | 555 | -12.31/1.5151e-171 | -12.31/3.4905e-178 | / |
| *Bd*-milR951 | 466 | 2676 | 0 | 0 | 12.019/6.6048e-138 | 14.464/0 | / |
| *Bd*-milR1151a-3p | 1887 | 0 | 1865 | 0 | 14.037/0 | / | 14.056/0 |
| *Bd*-milR1432 | 1104 | 0 | 324 | 0 | 13.264/0 | / | 11.530/8.1602e-98 |
| *Bd*-milR1527 | 482 | 0 | 385 | 0 | 12.068/1.2870e-142 | / | 11.779/4.2901e-116 |
| *Bd*-milR156c | 3532 | 0 | 3975 | 0 | 14.941/0 | / | 15.147/0 |
| *Bd*-milR2118p | 126 | 0 | 237 | 0 | 10.133/8.1887e-38 | / | 11.079/9.5957e-72 |
| *Bd*-milR2275a-5p | 10522 | 0 | 15305 | 0 | 16.516/0 | / | 17.092/0 |
| *Bd*-milR8181 | 120 | 0 | 50 | 0 | 10.062/4.7815e-36 | / | 8.8349/1.0432e-15 |
| *Bd*-milR847-5p | 1670 | 0 | 1269 | 0 | 13.861/0 | / | 13.500/0 |
| *Bd*-milR902i-3p | 304 | 0 | 297 | 0 | 11.403/3.2464e-90 | / | 11.405/1.0057e-89 |
| *Bd*-milR2621 | 10 | 0 | 0 | 17 | / | -7.287/3.5177e-06 | -7.287/7.2067e-6 |
| *Bd*-milR5711 | 0 | 4784 | 1973 | 0 | / | 15.302/0 | 14.137/0 |
| *Bd*-milR156f-3p | 1385 | 0 | 0 | 0 | 13.591/0 | / | / |
| *Bd*-milR1149.2 | 27083 | 0 | 0 | 0 | 17.880/0 | / | / |
| *Bd*-milR167c | 21 | 1197 | 0 | 0 | 10.530/1.3725e-49 | / | / |
| *Bd*-milR2108b | 166 | 0 | 0 | 0 | 10.530/1.3725e-49 | / | / |
| *Bd*-milR395g-5p | 1019 | 0 | 0 | 0 | 13.148/1.0494e-300 | / | / |
| *Bd*-milR5283 | 12341 | 0 | 0 | 0 | 16.746/0 | / | / |
| *Bd*-milR6214 | 33 | 0 | 0 | 0 | 8.2001/1.9574e-10 | / | / |
| *Bd*-milR6232b-5p | 31056 | 0 | 0 | 0 | 18.078/0 | / | / |
| *Bd*-milR7491 | 10518 | 0 | 0 | 0 | 16.516/0 | / | / |
| *Bd*-milR7999-5p | 29586 | 0 | 0 | 0 | 18.008/0 | / | / |
| *Bd*-milR9730 | 7928 | 0 | 0 | 0 | 16.108/0 | / | / |
| *Bd*-milR9742 | 369 | 0 | 0 | 0 | 11.683/2.3763e-109 |  |  |
| *Bd*-milR1041 | 795 | 0 | 689 | 1009 |  | -13.17/2.9643e | / |
| *Bd*-milR1147.2 | 0 | 7617 | 0 | 0 | / | 15.973/0 | / |
| *Bd*-milR160a | 0 | 19403 | 0 | 0 | / | 17.322/0 | / |
| *Bd*-milR171g-5p | 0 | 19605 | 0 | 0 | / | 17.337/0 | / |
| *Bd*-milR2091-5p | 0 | 1093 | 0 | 0 | / | 13.172/3.6825e | / |
| *Bd*-milR399f-5p | 0 | 2595 | 0 | 0 | / | 14.420/0 | / |
| *Bd*-milR4374a | 0 | 8856 | 0 | 0 | / | 16.191/0 | / |
| *Bd*-milR4414-5p | 202 | 0 | 225 | 370 | / | -11.73/4.8868e-119 | / |
| *Bd*-milR482a | 0 | 4379 | 0 | 0 | / | 15.175/0 | / |
| *Bd*-milR4994-3p | 0 | 346 | 0 | 0 | / | 11.513/1.1425e-98 | / |
| *Bd*-milR5072 | 0 | 5089 | 0 | 0 | / | 15.391/0 | / |
| *Bd*-milR5083 | 63 | 0 | 48 | 73 | / | -9.389/4.3910e-24 | / |
| *Bd*-milR5568f-3p | 0 | 2106 | 0 | 0 | / | 14.118/0 | / |
| *Bd*-milR5636 | 0 | 3677 | 0 | 0 | / | 14.922/0 | / |
| *Bd*-milR5663-3p | 0 | 102 | 0 | 0 | / | 9.7510/1.3788e-29 | / |
| *Bd*-milR5715 | 419 | 0 | 186 | 282 | / | -11.33/6.6590e-91 | / |
| *Bd*-milR5809 | 907 | 0 | 943 | 1295 | / | -13.53/0 | / |
| *Bd*-milR6194 | 0 | 130 | 0 | 0 | / | 10.100/1.6297e-37 | / |
| *Bd*-milR6223-5p | 0 | 6646 | 0 | 0 | / | 15.776/0 | / |
| *Bd*-milR6229-5p | 0 | 12694 | 0 | 0 | / | 16.710/0 | / |
| *Bd*-milR6234a-3p | 0 | 13940 | 0 | 0 | / | 16.845/0 | / |
| *Bd*-milR6267c-3p | 0 | 257 | 0 | 0 | / | 11.084/1.8017e-73 | / |
| *Bd*-milR6473 | 0 | 18 | 0 | 0 | / | 7.2485/0 | / |
| *Bd*-milR6474 | 0 | 16726 | 0 | 0 | / | 17.108/0 | / |
| *Bd*-milR7763-5p | 0 | 2020 | 0 | 0 | / | 14.058/0 | / |
| *Bd*-milR8123-3p | 989 | 443 | 723 | 827 | / | -1.022/5.0703e-35 | / |
| *Bd*-milR8130-5p | 0 | 713 | 0 | 0 | / | 12.556/1.4200e-202 | / |
| *Bd*-milR862b | 0 | 473 | 0 | 0 | / | 11.964/1.2631e-134 | / |
| *Bd*-milR8726 | 0 | 726 | 0 | 0 | / | 12.582/2.9629e-206 | / |
| *Bd*-milR1030h | 0 | 0 | 3925 | 0 | / | / | 15.129/0 |
| *Bd*-milR1035 | 0 | 0 | 22 | 0 | / | / | 7.6504/2.5636e-7 |
| *Bd*-milR1127a | 0 | 0 | 594 | 0 | / | / | 12.405/1.0084e-178 |
| *Bd*-milR169k | 0 | 0 | 97 | 0 | / | / | 9.7909/8.5980e-30 |
| *Bd*-milR173-3p | 0 | 0 | 26248 | 0 | / | / | 17.870/0 |
| *Bd*-milR2609a | 0 | 0 | 745 | 0 | / | / | 12.732/5.6894e-224 |
| *Bd*-milR3630-5p | 0 | 0 | 1002 | 0 | / | / | 13.159/5.5282e-301 |
| *Bd*-milR395a | 0 | 0 | 6129 | 0 | / | / | 15.772/0 |
| *Bd*-milR395c-3p | 0 | 0 | 1638 | 0 | / | / | 13.868/0 |
| *Bd*-milR396c | 0 | 0 | 157 | 0 | / | / | 10.485/9.0122e-48 |
| *Bd*-milR4228-3p | 0 | 0 | 9935 | 0 | / | / | 16.469/0 |
| *Bd*-milR482d | 0 | 0 | 41 | 0 | / | / | 8.5485/5.1919e-13 |
| *Bd*-milR5565e | 0 | 0 | 57 | 0 | / | / | 9.0239/8.3326e-18 |
| *Bd*-milR6174 | 0 | 0 | 430 | 0 | / | / | 11.939/1.4053e-129 |
| *Bd*-milR6182 | 0 | 0 | 3102 | 0 | / | / | 14.790/0 |
| *Bd*-milR7738-5p | 0 | 0 | 4091 | 0 | / | / | 15.189/0 |
| *Bd*-milR8051-5p | 0 | 0 | 31 | 0 | / | / | 8.1452/5.1514e-10 |
| *Bd*-milR825-3p | 57 | 32 | 0 | 34 | / | / | -8.287/5.2102e-11 |
| *Bd*-milR827 | 0 | 0 | 49737 | 0 | / | / | 18.793/0 |
| *Bd*-milR9657b-5p | 0 | 0 | 282 | 0 | / | / | 11.330/3.1434e-85 |
