# Supplementary material for: Comprehensive analysis of full genome sequence and Bd-milRNA/target mRNAs to discover the mechanism of hypovirulence in Botryosphaeria dothidea strains on pear infection with BdCV1 and BdPV1
Source: IMA Fungus. 2019 Jun 7;10:3. doi: 10.1186/s43008-019-0008-4 (PMC7325678; doi:10.1186/s43008-019-0008-4)
Supplement: Supplementary file 34 — Table S18. Three known Bd-milRNAs sequences were cloned and sequenced by stem-loop RT-PCR (DOCX 50 kb) [file 43008_2019_8_MOESM34_ESM.docx]

Additional file 34: **Table S18** Three known *Bd*-milRNAs sequences were cloned and sequenced by stem-loop RT-PCR

*Bd-*milR172


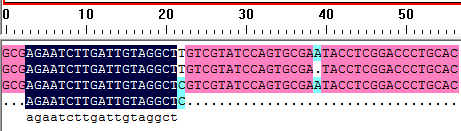

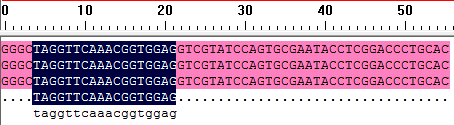

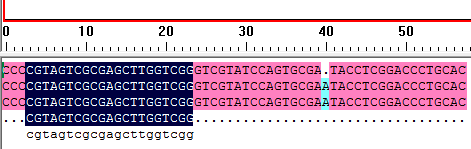


*Bd-*milR8635

*Bd-*milR5636
